# 65,000-years of continuous grinding stone use at Madjedbebe, Northern Australia

**DOI:** 10.1038/s41598-022-15174-x

**Published:** 2022-07-11

**Authors:** Elspeth H. Hayes, Richard Fullagar, Judith H. Field, Adelle C.F. Coster, Carney Matheson, May Nango, Djaykuk Djandjomerr, Ben Marwick, Lynley A. Wallis, Mike A. Smith, Chris Clarkson

**Affiliations:** 1 MicroTrace Archaeology, PO Box 102, Wollongong, NSW 2520 Australia; 2 Centre for Archaeological Science, School of Earth, Atmospheric and Life Sciences, Faculty of Science, Medicine and Health, University of Wollongong, Wollongong, NSW 2522 Australia; 3 School of Social Sciences, University of Western Australia, Perth, WA 6009 Australia; 4 History and Archaeology, College of Humanities, Arts and Social Sciences, Flinders University, Adelaide, SA 5042 Australia; 5 School of Biological, Earth and Environmental Sciences, The University of New South Wales, Sydney, NSW 2052 Australia; 6 School of Mathematics and Statistics, University of New South Wales, Sydney, NSW 2052 Australia; 7 School of Environment and Science, Griffith University, Nathan, QLD 4111 Australia; 8 Gundjeihmi Aboriginal Corporation, Jabiru, NT 0886 Australia; 9 Department of Anthropology, University of Washington, Seattle, WA 98195 USA; 10 Griffith Centre for Social and Cultural Research, Griffith University, Brisbane, QLD 4111 Australia; 11 Centre for Historical Research, National Museum of Australia, Canberra, ACT 2601 Australia; 12 Department of Archaeology, Max Planck Institute for the Science of Human History, Kahlaische Strasse 10, 07745 Jena, Germany; 13 Australian Research Council (ARC) Centre of Excellence for Australian Biodiversity and Heritage, University of Wollongong, Wollongong, NSW 2522 Australia; 14 School of Social Science, University of Queensland, St Lucia, QLD 4072 Australia

**Keywords:** Anthropology, Archaeology

## Abstract

Grinding stones and ground stone implements are important technological innovations in later human evolution, allowing the exploitation and use of new plant foods, novel tools (e.g., bone points and edge ground axes) and ground pigments. Excavations at the site of Madjedbebe recovered Australia’s (if not one of the world’s) largest and longest records of Pleistocene grinding stones, which span the past 65 thousand years (ka). Microscopic and chemical analyses show that the Madjedbebe grinding stone assemblage displays the earliest known evidence for seed grinding and intensive plant use, the earliest known production and use of edge-ground stone hatchets (aka axes), and the earliest intensive use of ground ochre pigments in Sahul (the Pleistocene landmass of Australia and New Guinea). The Madjedbebe grinding stone assemblage reveals economic, technological and symbolic innovations exemplary of the phenotypic plasticity of *Homo sapiens* dispersing out of Africa and into Sahul.

## Introduction

Grinding stones and other ground implements are a fundamental component of the human technological panoply that first emerged in the Levant, Africa, and Europe from at least 780 ka ago^1–13^. These implements allowed nutritious hard-shelled, starchy and fibrous foods to be made comestible, and easily digested. Alongside cooking, grinding stones were particularly important in rendering tough foods more edible for infants and the elderly. Grinding stones are theorised to have played a key role in exploiting the arid and semi-arid zones of Australia, where grass seeds, hard-cased seeds and pulverised animals formed a vital component of the late Holocene Aboriginal diet^14–27^. Grinding stones also played a key role in pigment preparation and in the production and use of ground stone hatchets throughout many parts of Australia and New Guinea^28–31^. Australian site reports rarely document large numbers of grinding stones (including amorphous fragments and formal types of grindstone) except for the late Holocene^32^; and few artefacts have been subjected to usewear and residue analysis. For example, a review of seed grinding implements lists a total of 468 grinding stones from 14 sites, with a range of 1–89 per site^33,34^. Most of the grinding stones and all of the 73 formal artefacts classified as ‘seedgrinders’ are from Holocene levels^34^.

One other site, Nauwalabila, also located in the Kakadu region, may have grinding stones of comparable age (53.4 ± 5.4 ka and 60.3 ± 6.7 ka^35^) to Madjedbebe, but the reported assemblage is small (n = 43^36,37^), the grinding stones have not been analysed and the ages are contested^38^.

**Figure 1:**
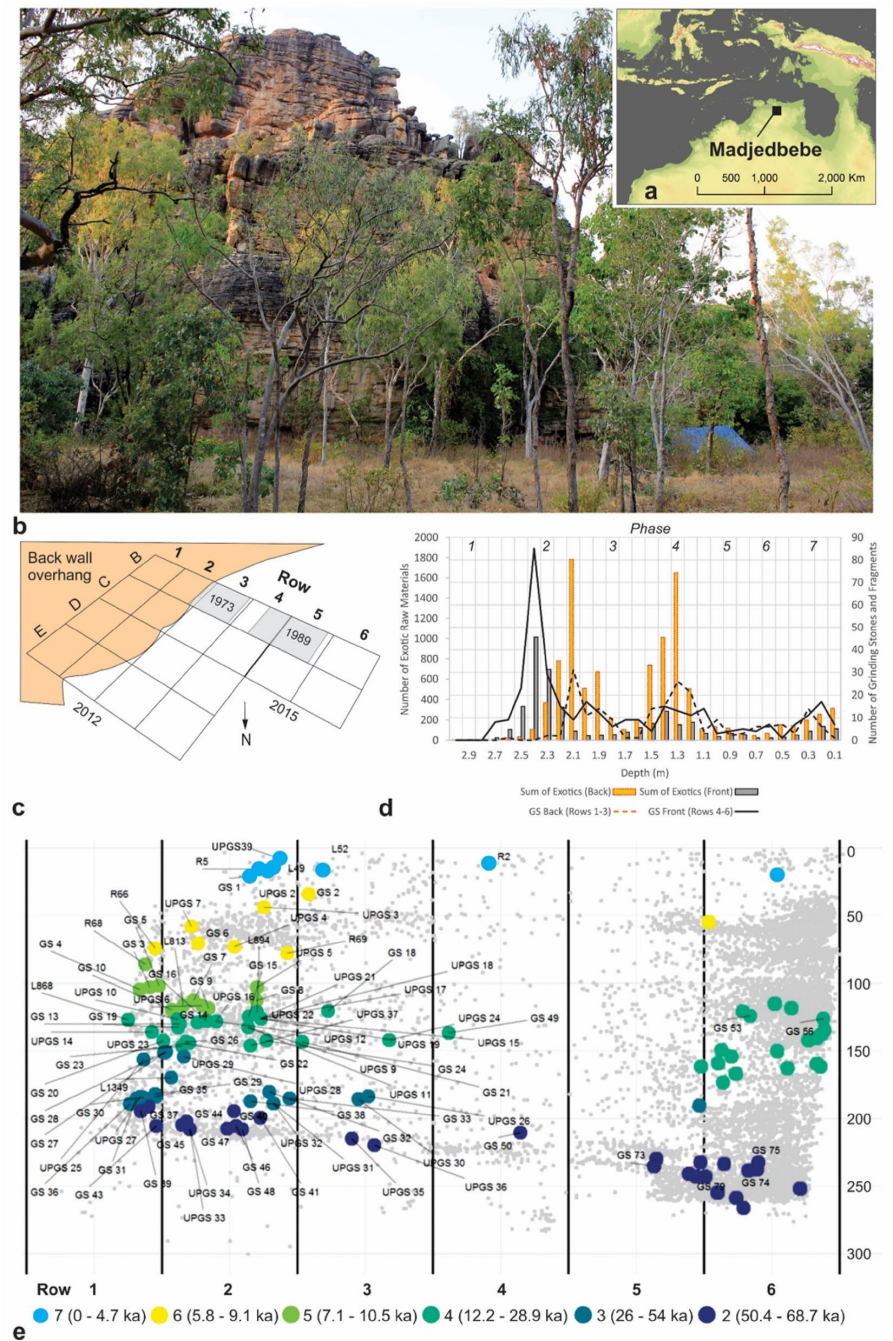
Location of Madjedbebe, site layout and the distribution of grinding stones. (**a**) Location of the site. Sea levels are shown at −80 m bsl equivalent to MIS 3; (b) Photo of the Madjedbebe and Djuwamba massif taken from the north. The blue tarp indicates the location of the excavation against the rockshelter wall (photo courtesy of Tiina Manne); (**c**) Grid layout of site showing 1973 (B3), 1989 (B4-5), 2012 (B1-E4) and 2015 (B5-C6) excavated areas and location of the back wall; (**d**) Frequency distribution of grinding stones and exotic raw materials by depth. The assemblage is divided into front (rows 5–6) and back (rows 1–3) to account for the 5° slope in stratigraphy from the back to the front; (**e**) Location of 3-D plotted grinding stones at Madjedbebe colour-coded by Phase. Grey dots represent lithics, ground ochre and other artefacts. Row 1 is closest to the back wall of the rockshelter and Rows 5 and 6 are located outside the dripline. Rows 4 and 5 show fewer plotted artefacts as B4 and part of B5 they were excavated in 1989 and artefacts were not plotted in situ.

Recent excavations at Madjedbebe (Fig. 1a)^39^, a rockshelter in Mirarr Country in northern Australia, have extended the antiquity of grinding stone use in Australia. Here we report on the function of 104 grinding stones with macroscopic traces of use that were available for microscopic study up until 2020–1. With more recent counts of smaller fragments from bulk and 3-mm-sieve sediments, we estimate a total of 563 grinding stones (including fragments) from the site, spanning its entire period of human occupation (Table 1). Functional analyses (microscopic usewear, residue and biochemical analyses) were carried out on 104 of these grinding stones (18.5% of the complete grinding stone assemblage), including 29 artefacts from the earliest phase of occupation (Phase 2) dated to between 68.7 and 50.4 ka, and two artefacts from uncertain (probably late Holocene) context. These microscopic and chemical analyses yield significant new insights into the diet, technology and symbolism of the first human colonists of Sahul.

**Table 1:** The age range expressed as the 95.4% confidence interval (CI) obtained from the modelled start and end age estimates for the seven phases of the Madjedbebe sequence, corresponding MIS and climatic conditions^1,2,3^, and number of grinding stones (including fragments) recovered and analysed from each phase.

Phase	95.4% CI age range (ka)	Environmental conditions	Number of grinding stones (including fragments)	Number of analysed artefacts
1	> 65.4	Variable glacial and interglacial conditions at the end of MIS 5a and 5b and the beginning of MIS 4. This corresponds to the sediments below Phase to the lowest excavated deposits	0	0
2	68.7–50.4	A period of relative aridity at the end of MIS 4	181	29
3	54.0–26.0	A variable period of increased precipitation during MIS 3/2	112	16
4	28.9–12.2	The Last Glacial Maximum peaking at c.22–18 ka	111	33
5	10.5–7.1	A period of increased precipitation (Early Holocene Optimum) and rising sea levels culminating in the marine transgression c.8 ka at the end of MIS 2 and beginning of MIS 1	53	15
6	9.1–5.8#	A period of increased precipitation and the formation of estuarine environments along the South Alligator River	42	4
7	4.7–0.0#	A period of intensified El Niño Southern Oscillation (ENSO) conditions, more variable and decreased precipitation. The transition from estuarine to freshwater vegetation communities on the Alligators Rivers’ lowlands takes place at this time due to increasing sedimentation from approximately 3.3 ka, culminating in the formation of the Magela Creek floodplain freshwater wetlands at 1333–1062 cal BP^38,73^	64	5
Uncertain				2
			563	104

#Age range for Phases 6 and 7 are based on the oldest and youngest OSL and/or ^14^C ages.

### Madjedbebe

Madjedbebe is a rock shelter located at the foot of an outlying massif adjacent to the western Arnhem Land plateau located within Mirarr Country in the Alligator Rivers region of the Northern Territory (Fig. 1a and b). Twenty 1 × 1 m squares were excavated to a maximum depth of 4 m between 1979 and 2015^32,39,40^ (Fig. 1c), with the three-dimensional coordinates of ~ 11,000 artefacts and other archaeological features (hearths, burials and pits) were recorded. Macrobotanical remains were recovered by flotation, and a series of 26 AMS radiocarbon (^14^C) and 52 optically stimulated luminescence (OSL) ages were obtained for the site (see Supplementary Material Section 1 for a summary of the published site chronology). Seven artefact phases were identified, with Phases 2–7 associated with human occupation (Table 1). Trampling experiments^41^ and geoarchaeological analyses^1^ of the organic and inorganic components of the deposit suggest that there has been only small-scale post-depositional disturbance at the site and there is no evidence of extensive reworking of the deposit by termites^42–44^ (See Supplementary Material Section 1).

Artefacts occur in three dense bands in Phases 2, 4, 6 and 7, with fewer artefacts in intervening phases^39^. Changes in raw material use, stone working technology and climatic conditions occur across the phases. A start and end age were determined for each phase using a Bayesian modelling approach that included all OSL age estimates^39^. Ages and age ranges used throughout are based on these start and end age estimates and their random-only errors at 95.4% probability^39^). Phase 1 (> 65.4 ka) represents accumulation of a sand sheet during marine isotope stage (MIS) 5 that contains a low density of stone artefacts near the top of the Phase. Phase 2 (68.7–50.4 ka, MIS 4 and extending into MIS 3) is associated with a cool dry climate with sea-level at ~ 50 m below modern sea-level (bmsl) when Madjedbebe was ~ 300 km from the nearest shoreline^45^. A large and dense stone artefact assemblage (n = < 10,000), rich in exotic raw materials occurs in Phase 2, including stone points, thinning flakes and centripetal core technology (Fig. 1d). Exotic raw materials include chert, silcrete, dolerite, hornfels and tuff, none of which are known to occur closer than 25 km from the site, which sits in an outlier of Proterozoic Kombolgie sandstone from the Arnhem Land Plateau. Phase 3 (54.0–26.0 ka, MIS 3 and extending into MIS 2) falls within a period marked by a variable and wetter climate with higher sea levels and a stronger monsoon from c.50 ka^46,47^. Flaked stone artefacts made from exotic raw materials are uncommon in this phase. Phase 4 (28.9–12.2 ka, MIS 2) corresponds to dry Last Glacial Maximum (LGM) conditions with sea levels dropping to − 120 m bmsl^48^. During Phase 4, a pronounced increase in stone artefact discard is documented, along with increased importation of exotic raw materials and a peak in bipolar technology. Phases 5–7 are Holocene units. Phase 5 (10.5–7.1 ka, MIS 1) coincides with a period of rapid sea level rise and the establishment of a wetter climate corresponding to the Holocene climatic optimum and is associated with low artefact density and low abundance of exotic flaked stone. The chronology for Phases 6 and 7 are poorly constrained by the OSL Bayesian age model for the site. Age ranges are instead based on the range of calibrated ^14^C and OSL ages for each of the Phases. Phase 6 (9.1–5.8 ka) sees a continuation of wetter conditions with the establishment of estuarine conditions close to the site, reflected in the presence of a large shell midden dominated by mangrove dwelling species. Artefact density again peaks as bifacially flaked stone points and bone point technology appear at this time and thinning flakes associated with invasive retouching reappear. The most recent phase of occupation, Phase 7 (4.7–0.0 ka), corresponds to drier and more variable climate with a period of intensified El Niño–Southern Oscillation climatic conditions and more variable and decreased precipitation. Bifacial point technology is most common at this time. From approximately 3.3 ka, the Alligators Rivers lowlands transition from estuarine to freshwater vegetation communities, culminating in the formation of the Magela Creek floodplain freshwater wetlands within 1 km of the site during the last 1 ka^39,49^.

Grinding stones (including fragments and complete implements) are present in varying abundance throughout Phases 2–7 (Table 1). Grinding stone frequency peaks in the drier Phases 2, 4 and 7 (Table 1, Fig. 1d), presumably as the plant component of the foraging economy shifted to incorporate lower ranked resources during drier times when higher ranked foods were less available^50^. The majority of *in situ* grinding stones were found just outside the dripline (rows 5 and 6 in the excavation grid) or against the rockshelter wall (rows 1 and 2) (Fig. 1e).

## Results

We used both qualitative and quantitative methods to identify the function(s) of 104 grinding stones recovered from Madjedbebe and examine morphology, usewear and residue traces.

### Grinding stone class/morphology

**Figure 2:**
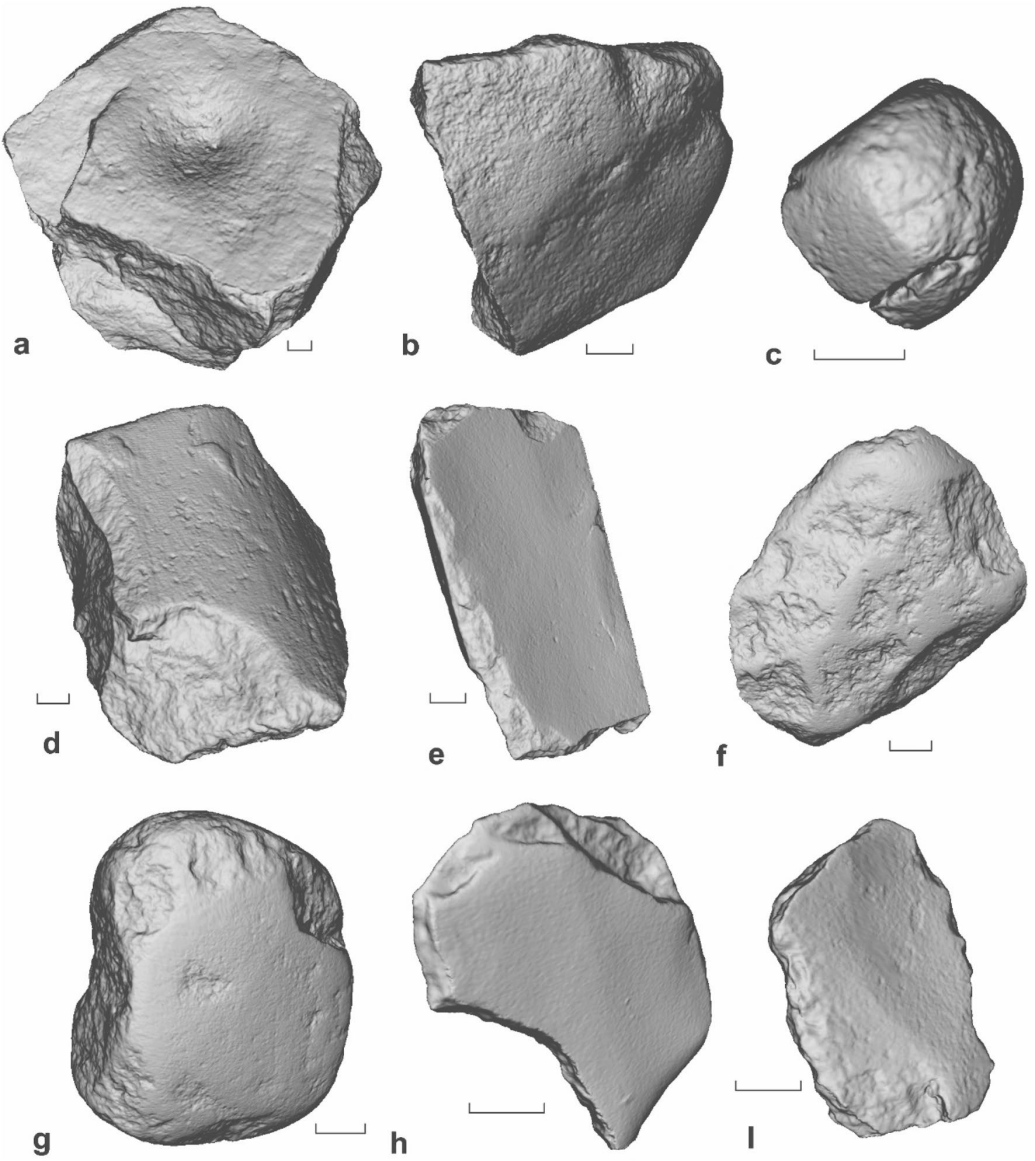
3-D scans of grinding stones from Madjedbebe. (**a**) GS32, C2-C3/37, Phase 2, mortar; (**b**) GS20, E1/27, Phase 4, filing stone; (**c**) UPGS26, C3/35, Phase 3, pounding stone fragment; (**d**) GS73, B5/52, Phase 2, millstone fragment; (**e**) GS79, B6/54, Phase 2, whetstone. (**f**) L49, C2/5, Phase 7, upper hand stone; (**g**) L52, C3/5, Phase 7, upper hand stone; (**h**) GS36, C1/35, Phase 2, tabular fragment; (**i**) GS50, C4/45, Phase 2, tabular fragment. Scale bars are 2 cm.

The sample of 104 grinding stones analysed in this study exhibited a wide range of forms, surface profiles and macroscopic wear indicative of a range of specialised activities (Fig. 2). Although 76.0% of the analysed grinding stones were recovered as small, tabular fragments (n = 79 with a median mass of 143 g), many retained sufficient features in gross morphology to indicate their functional class (i.e., upper stone, lower stone, filing stone, see Supplementary Material Section 2). Formal grinding stone classes were also recognised among the fragments and complete implements, and included mortars, millstones, top stones, pounders and whetstones^51^ (Supplementary Material Section 2). Nearly all of the grinding stones were made from fine to medium grained local or exotic sandstone (n = 94, 90.4%) (Supplementary Material, Table S1).

A single ground surface was identified on the majority of the grinding stones (76 of the 104 analysed stones, 73%), which were generally flat or, less commonly convex, in cross-section. Twenty-eight artefacts (~ 27%) displayed two or more ground surfaces, making a total of 134 ground surfaces amongst the 104 analysed artefacts (Supplementary Material, Table S1). To determine whether the stones were used as upper (active), lower (passive) or filing (passive) stones, we recorded the cross-section of the ground surfaces (e.g., convex, concave, flat, undulating), the size (length, width and height) of the stones, and the locations of grinding wear (Supplementary Material Section 2).

Fifty artefacts (~ 48%) were classified as coupled stones—grinding stones that were used in a pair as either upper or lower stones to process an intermediate material. These included 25 upper stones (48% of the coupled stones, 24% of the analysed grinding stone assemblage), 17 lower stones (~ 34% of the coupled stones, 16% of the analysed grinding stone assemblage) and eight that could not be distinguished as upper or lower stones but still had other traces to indicate they were used as coupled stones (e.g., stone-on-stone grinding wear documented under the microscope). Upper and lower stones were distinguished by their size (upper stones usually being small enough to fit comfortably in the hand), and the cross-section of their ground surfaces.

All of the upper stones displayed at least one or more convex ground surfaces, while the lower stones typically displayed flat or undulating ground surfaces (n = 12) or, less commonly, concave surfaces or depressions (n = 5). These included three mortar stones with macroscopically visible pitted depressions (GS56, GS32, and GS75 from Phases 4 and 2, respectively) (Fig. 2a), and a relatively large millstone fragment (GS73 from Phase 2) with deep, partial grooves (Fig. 2d), similar to those documented on Australian seed grinding implements in the arid interior^52^. Not surprisingly, the upper stones were much smaller than the lower stones, the largest of which had a mass of 539 g (L49, Fig. 2f), while some of the complete lower stones weighed up to 8 kg.

A further 32 artefacts (~ 31% of the total analysed grinding stone assemblage) were classified as filing stones—grinding stones that were used as individual stone “files” to process and shape the worked material and generally displaying flat (n = 25) grinding surfaces. Seven of these artefacts also had wear consistent with their use as coupled stones, indicating that these implements were either multifunctional tools or had been used opportunistically for multiple tasks.

The remaining 29 artefacts could not be assigned a functional class as they did not retain sufficient features to confidently identify them as either coupled or filing stones. Traces of surface rejuvenation (i.e., pitting to roughen the grinding surface) were absent, with only three artefacts displaying macroscopically visible pitting from use and manufacture (see above). Negative flake removals from the surface/perimeter were documented on just two artefacts (L49 and GS 79 from phases 7 and 2, respectively), one of which was deliberately split prior to use (GS 79) (Fig. 2e, f).

### Usewear

**Figure 3:**
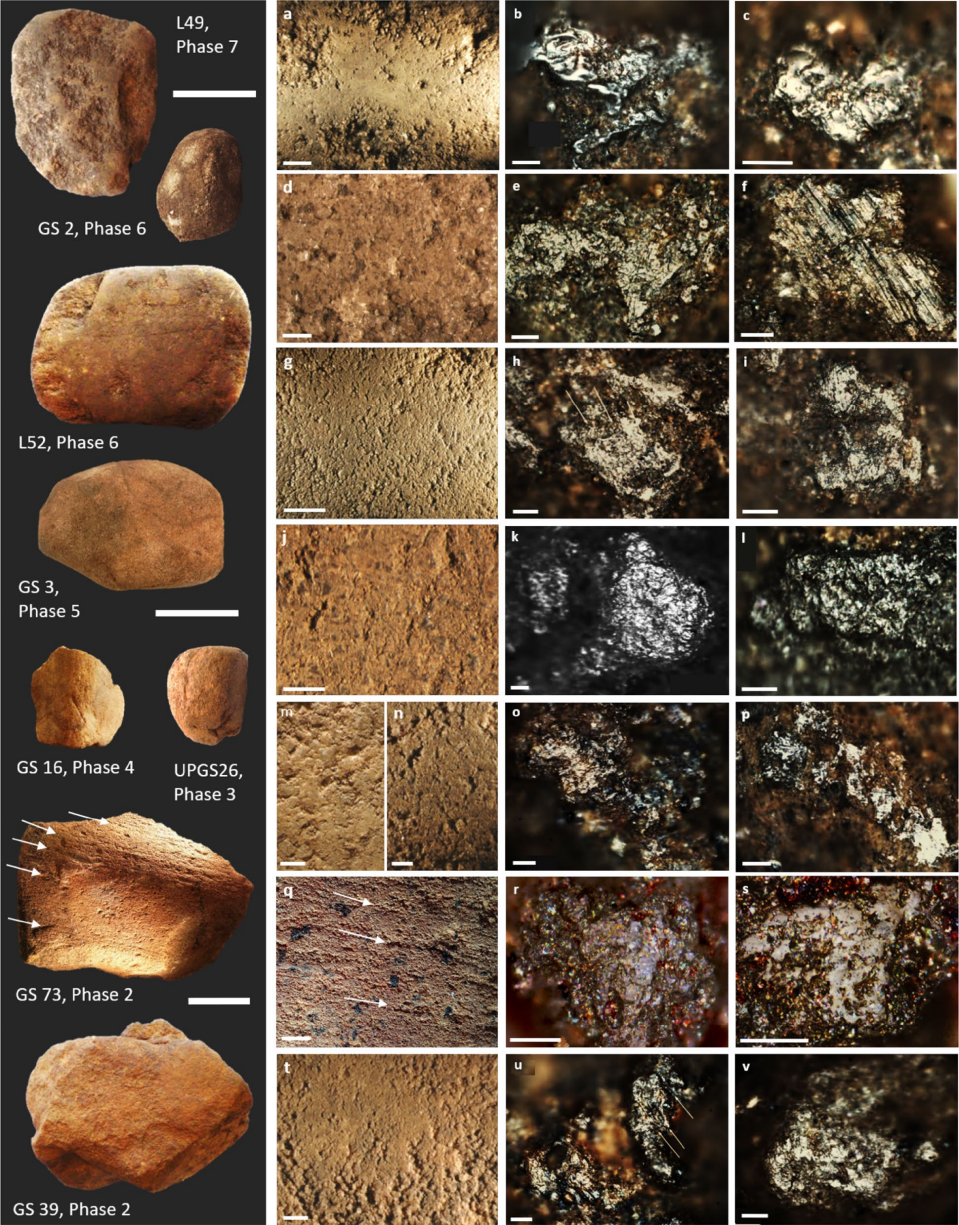
Examples of grinding stones from Madjedbebe with usewear consistent with plant processing/seed grinding from Phases 7–2. Under low magnification, the more elevated quartz grains on the surface of the sandstone are levelled and sometimes striations are visible. Under high magnification, use-polish is very bright and restricted to the more elevated part of the relatively rough quartz grains, creating a reticular or net-like pattern with a distinct boundary between the polished and unpolished zones to indicate the processing of a harder plant material such as seeds. In instances where the use-polish extends into the lower recesses of the grains (e.g., b, c), we infer the processing of softer plant materials. (**a**–**c**) Usewear on L49 from Phase 7; (**d**–**f**) usewear on GS2 from Phase 6; (**g**–**i**) usewear on L52 from Phase 6; (**j**–**l**) usewear on GS3 from Phase 5; (**m**, **o**) usewear on GS16 from Phase 4; (**n**, **p**) usewear on UPGS26 from Phase 3; (**q**–**s**) usewear on GS73 from Phase 2; (**t**–**v**) usewear on GS39, Phase 2. Scale bars for artefact images are 5 cm; scale bars for low magnification images vary: (**a**, **g**) 5 mm; (**m**, **n**, **q**, **t**) 2 mm; (**d**, **j**) 1 mm; scale bars for high magnification images are 0.05 mm.

**Figure 4:**
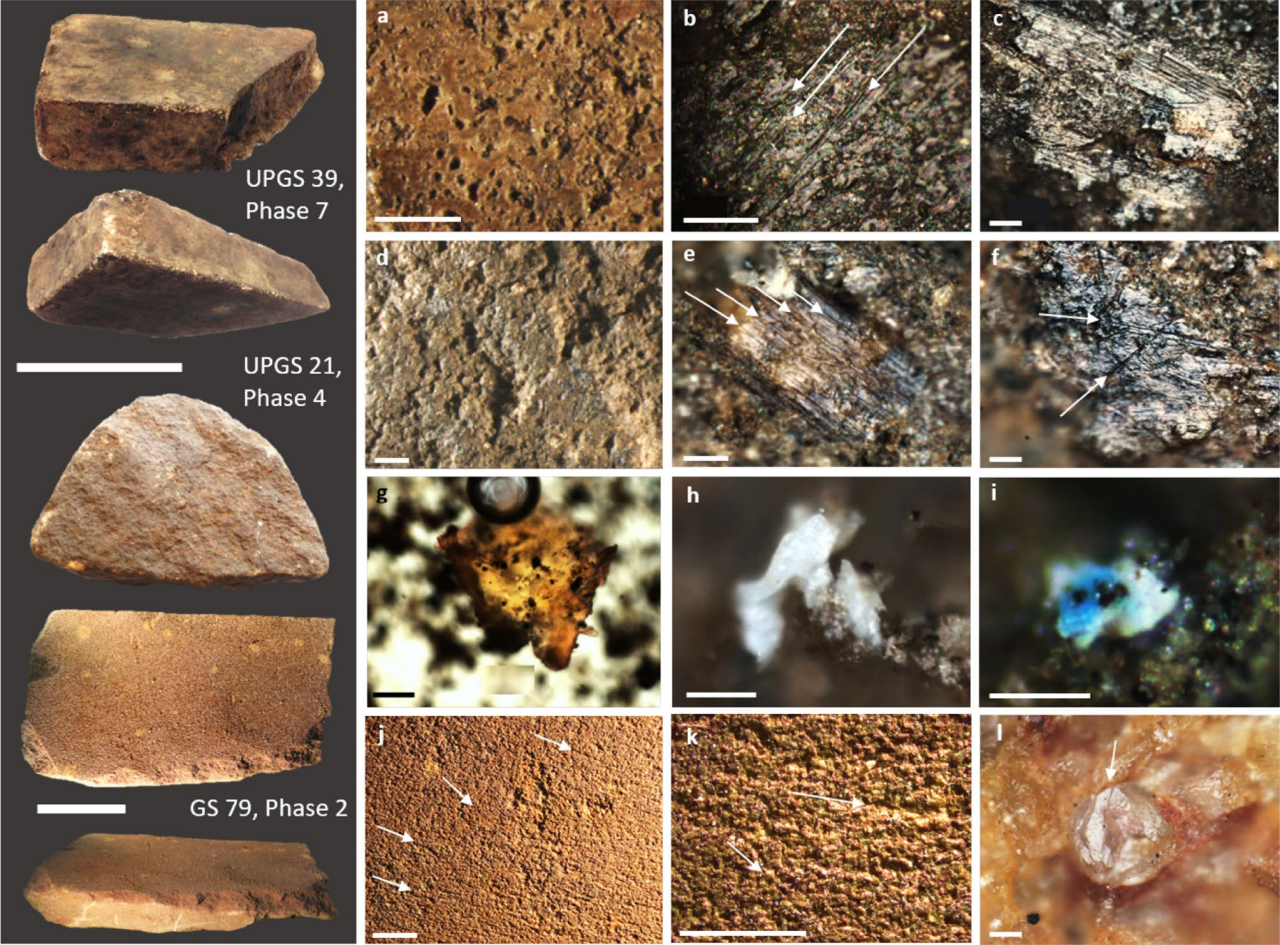
Examples of stone and bone files from Madjedbebe: UPGS39 from Phase 7, UPGS21 from Phase 4 and GS79 from Phase 2. (**a**) levelled surface of UPGS 39; (**b**) use-polish on the more elevated zones of the stone micro-topography with numerous striations on UPGS39; (**c**) metal residues with evident smearing and directionality on UPGS39; (**d**) levelled grains on the surface of UPGS21; (**e**, **f**) striated (arrows) and undulating use-polish on UPGS21; (**g**) collagen tissue stained with Orange G from pipette extraction sampled from UPGS21; (**h**) organic residue cf. bone on UPGS21; (**i**) white residue with blue mineral secretion, cf. bone and vivianite, on UPGS21; (**j**, **k**) surface of GS79 with removed grains and surface striations/scratches; (**l**) quartz grain on the surface of GS79, note that the grain is fractured (arrow) from contact with a hard material (e.g., stone). Scale bars for artefact images are 5 cm; scale bars for low magnification images are 1 mm; scale bars for high magnification images are 0.05 mm image i. is 0.02 mm.

**Figure 5:**
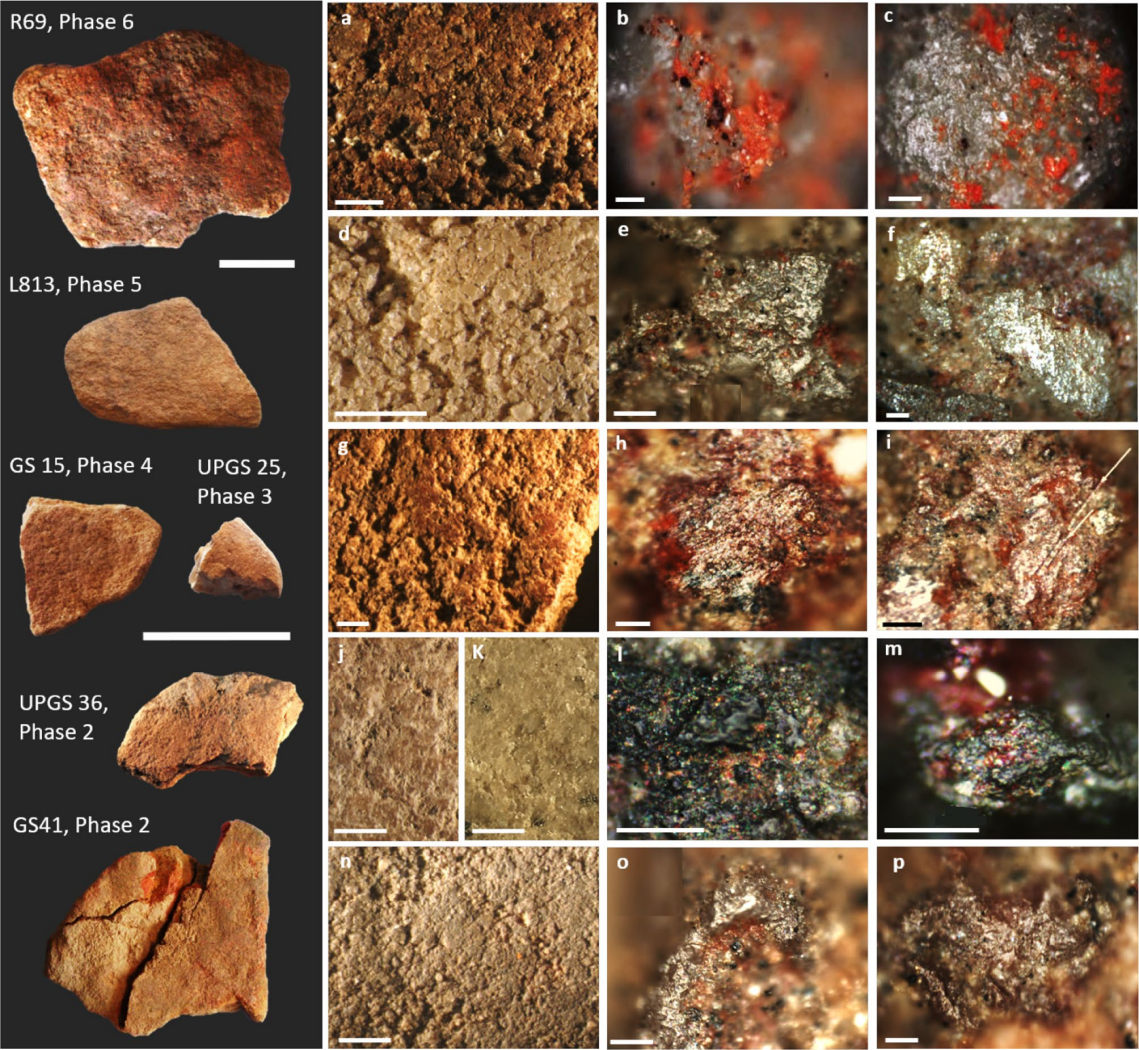
Examples of grinding stones from Madjedbebe with usewear and residues consistent with processing red pigment. Note that the red mineral grains occur in the lower recesses of the stone micro-topography and occur with an undulating use-polish. (**a**–**c**) Usewear and red pigment residues on R66 from Phase 6; (**d**–**f**) usewear and red pigment residues on L813 from Phase 5; (**g**–**i**) usewear and red pigment residues on GS15 from Phase 4; (**j**, **l**) usewear and red pigment residues on UPGS25 from Phase 3; (**k**, **m**) usewear and red pigment residues on UPGS36 from Phase 2; (**n**–**p**) usewear and red pigment residues on GS41 from Phase 2. Scale bars for artefact images are 5 cm; scale bars for low magnification images are 2 mm; scale bars for high magnification images are 0.05 mm.

Macroscopic grinding wear was documented on all analysed grinding stones and included levelled, undulating or pecked surfaces comprised of levelled or well-rounded grains, surface striations and visible pits or depressions (Supplementary Table S3) (Figs 3, 4, 5). Fifty-three of the 104 analysed artefacts possessed usewear traces that were consistent with the processing of one or more kinds of material (e.g., plants, haematite, stone), as indicated by the presence of use-polish, striations and other grain modifications, documented under low and high magnification (Supplementary Material Section 3; Supplementary Tables S2 and S3). The remaining 51 artefacts had usewear traces to indicate they were used for grinding/pounding activities but the usewear was not diagnostic of worked material.

Usewear indicative or suggestive of plant processing was recognised on 40 grinding stones (~ 38%) from all occupation phases (Supplementary Table S3). Twenty-six artefacts had usewear consistent with the grinding of softer plant material (e.g., roots, leaves and underground storage organs), recognised by a reticular (net-like) use-polish that extended into the lower microtopographic regions of the grains^53,54^ (e.g., Fig. 3; Supplementary Tables S2 and S3). A further 16 artefacts had usewear typical of processing harder plant materials (e.g., seeds), characterised by the occurrence of a bright, well-developed reticular use-polish that was restricted to the highest points of the quartz grain micro-topography (e.g., Fig. 3; Supplementary Tables S2 and S3). Less commonly, artefacts displayed usewear consistent with the processing of both hard and soft plant materials (n = 2, GS39 and L49, Fig. 3a–c; t–v).

Usewear consistent with stone filing was documented on five artefacts and included surface levelling with microscopically levelled grains, a high frequency of striations and micro-scarring of quartz grains^53,54^ (Fig. 4; Supplementary Tables S2 and S3). These included two implements (GS3, UPGS4) with manufacture-ground surfaces along both sides of a single edge to form a bevel. A further two grinding stones (both of which also displayed usewear consistent with plant processing) also had evidence for stone working, including battered zones and scratches that indicate they were used for striking and buffing stones during flake manufacture.

Usewear consistent with the processing of pigments was present on 12 grinding stones and included a levelled surface with an undulating use-polish and a high frequency of relatively deep striations throughout^53,54^ (Fig. 5; Supplementary Tables S2 and S3). Usewear consistent with the processing of soft animal tissues (skin, meat) was not documented on grinding stones from Madjedbebe, although possible bone grinding wear was documented on one tool (UPGS21) from Phase 4 (Fig. 4d–f). This usewear is characterised by moderate grain rounding with a smooth-pitted/striated polish, which was documented on one of the four surfaces with diagnostic grinding wear^53,54^ (Supplementary Tables S2 and S3).

Taphonomic and weathering agencies have affected the appearance of usewear traces on some of the grinding stones, particularly on those from earlier deposits (Phases 2–4), this includes most stones recovered during the 2015 excavation due to their location outside the drip zone. Stones with weathered surfaces typically displayed less developed usewear with grain rounding over much of the artefact. Surface reduction causing modification to the usewear presumably results from sub-aerial weathering or subsurface movements of sediment grains (e.g., from bioturbation, trampling, water percolation, etc.). In some cases (e.g., GS5), weathering caused surface grain erosion that removed traces of use-polish.

### Residues

#### Microscopically visible residues

Sampling for residues was undertaken on each of the ground surfaces (n = 140) using a variable pipette. Residues were then documented under a transmitted light microscope with the aid of various stains (Supplementary Material, Section 5). Microscopic organic residues were considered to be use-related if they occurred in high densities and in combination with multiple residues from the same origin (i.e., multiple plant tissues, starch, phytoliths, etc). Residues that could be identified microscopically were most abundant on artefacts from Phases 3–7.

Inorganic mineral crystals (e.g., haematite, clay and quartz) were found on all ground surfaces of 104 grinding stones. Plant material (e.g., cellulose fibres, lignified tissue, starch grains, phytoliths, perforation plates, sieve cells or bordered pits) was the most frequently documented residue and was found on 19 ground surfaces of 88 (84.6%) of the analysed grinding stones (Supplementary Table S4). Residues of animal origin were less common, and included collagen fibres, bone, feather barbules and hair fibres, and were documented on 28 ground surfaces of 21 (20.2%) of the analysed grinding stones (Fig. 4g–i; Supplementary Material, Table S4). Blood cells were not visually confirmed on any of the residue samples analysed. Collagen was identified as singular fibres and without other animal tissues on 19 (67.9%) of the 28 ground surfaces with animal tissue and could not confidently be linked with use. Similarly, individual hair or feather particles that were documented in isolation were generally not considered to be use-related.

Red and yellow pigments were identified on most of the grinding stones analysed for residues (n = 72, ~ 69%). Red pigment was documented on 93 of the 140 ground surfaces (~ 69%) and was observed directly on the ground surface and/or in residue extractions mounted on glass slides (Fig. 5). Four surfaces also displayed small amounts of yellow pigment, which were observed directly on the surface of the stone. The latter may be the result of incidental contact with yellow pigments, which were identified in all occupational phases, or may be the result of a chemical reduction/oxidation of red pigment. Most of the pigment residues were attributed to post-depositional contamination and post-excavation handling and sieving, as pigment clusters occurred with no apparent pattern that could be attributed to deliberate grinding. Artefacts were only associated with pigment processing if the pigment was (1) present in the lower interstices of the grinding surface; (2) covered more than 20% of the artefact surface; and (3) appeared “smeared” or had alignments or scratch marks running through the residues.

#### Starch grain analysis

A sub sample (n = 27) of the grinding stone assemblage (nine artefacts from Phases 3–7 and 18 artefacts from Phase 2) was analysed for the presence of starch. The grinding stones were chosen from those artefacts that had residues identified in the pipette extractions or had distinctive usewear indicative of plant processing. The number and size distribution of the starch grain assemblages is given in Table S5. Only starch grains with complete margins were digitised, and those that were damaged or obscured by detritus in the slide preparations were excluded.

**Figure 6:**
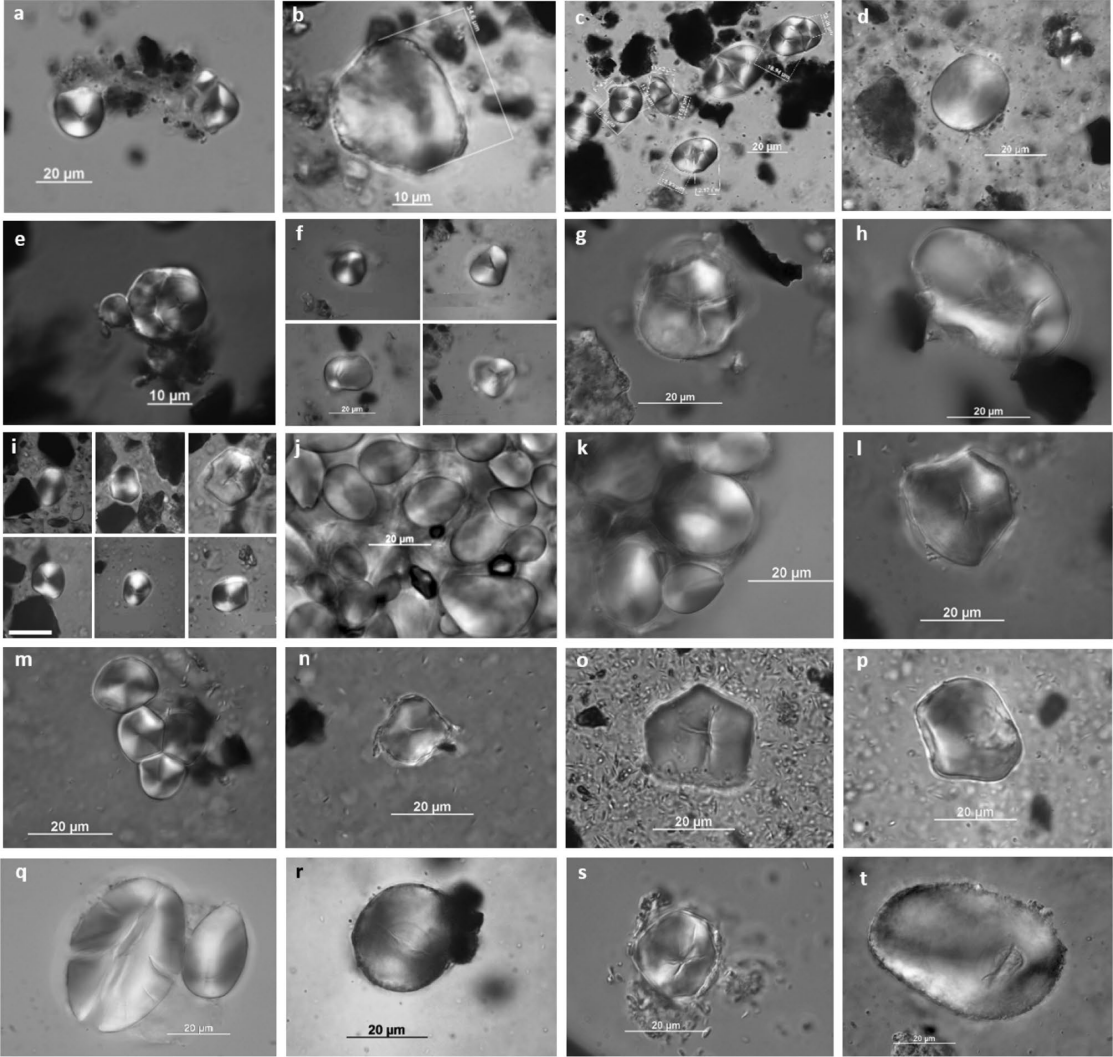
Examples of starch grains recovered from grinding stones from Madjedbebe submitted for starch grain analysis. Phase 7: (**a**, **b**) L49; (**c**) UPGS2. Phase 6: (**d**) UPGS4. Phase 5: (**e**) GS3. Phase 4: (**f**) UPGS14. Phase 3: (**g**, **h**) UPGS32. Phase 2: (**i**) GS9; (**j**–**l**) GS40; (**m**, **n**) GS48; (**o**, **p**) GS73; (**q**, **r**) GS 74; (**s**, **t**) GS86.

A range of starch grain morphologies was observed, and examples are presented in Fig. 6. The number of starch grains identified varied across all the artefacts sampled. One grinding stone (GS40 from Phase 2) yielded substantial numbers of starch grains (n = 143) (Fig. 6j–l). A further five artefacts, also from Phase 2, two with usewear and/or other residues consistent with seed or plant processing, yielded more than 40 starch grains per artefact (GS86, n = 60 grains; GS48, n = 35 grains; GS82, n = 48 grains; GS73, n = 41 grains; and GS74, n = 39 grains; Supplementary Table S5) (Fig. 6m–t).

Three of the grinding stones listed in Table S5 have had analyses published elsewhere (L49, GS3 and UPGS2, Fig. 6a–c, e), and the analyses demonstrated the presence of waterlily (*Nymphaea violacea*), cheeky yam (*Amorphopallus galbra*), long yam (*Dioscorea transversa*) and possibly kapok bush (*Cochlospermum fraseri*)^55^. The remaining artefacts in this study from Phases 2–7 (n = 21) yielded much lower frequencies of starch (Fig. 6d, f, g, h) (Supplementary Table S5), and included artefacts with usewear consistent with seed processing and distinct plant compounds detected with gas chromatography mass spectrometry (GC–MS) (e.g., GS39 and GS47 from Phase 2, see below). The taxonomic analysis of recovered starch from these grinding stones is the subject of a separate study. Taxa identified from ancient starch analysis match specimens in comparative reference collections of plants commonly utilised and collected by Mirarr people today^46^ and supplement data provided by charred plant remains at Madjedbebe^50^.

#### Biochemical testing

Biochemical tests (Supplementary Material, Section 7) were used to screen for the presence of biomolecules such as proteins, carbohydrates and sugars, fatty acids and haem^53,56^ on 94 of the 104 grinding stones. One or more of these biomolecules were detected on 81 of the measured artefacts (~ 86%) (Supplementary Material, Table S6B), indicating that many of the grinding stones were used to process some form of organic material or in contact with organic particles during some stage of their life history (e.g., during manufacture, curation, burial). Carbohydrates/sugars were the most commonly detected biomolecule, present on the ground surfaces of 56 artefacts (~ 60%), followed by fatty acids (n = 47 artefacts, 50%), proteins (n = 24 artefacts, ~ 26%) and haem, a principal component of red blood cells (n = 4, < 5%) (Supplementary Table S6B). As biochemical tests are only specific for molecule aggregates, and are unable to identify individual molecules, they are only suitable for providing an initial screening test for the presence of specific groups of organic compounds (see Supplementary Material, Section 7).

#### Absorbance spectroscopy

The absorbance characteristics of extracted residues (Supplementary Material, Section 7.2) indicated the presence of a range of non-specific organic material on all of the grinding stones that were analysed with this technique (n = 98). More detailed readings that identified other biological compounds were limited to nine artefacts and mostly indicate a plant origin. These compounds included phenolates and carboxyl groups; alcohols and plant sterols; alkaloids and carbon/nitrogen bonds; nucleic acids, phenols, and plant-based proteins and amino acids. Significantly, no animal proteins were detected on any of the measured samples. The lack of detectable absorbance in extractions sampled from most artefacts (n = 82, ~ 84%) is probably due to absence or the low sensitivity levels of the test, which typically requires large concentrations of compounds in order to be detected^53^.

#### Gas chromatography mass spectrometry (GC–MS)

**Figure 7:**
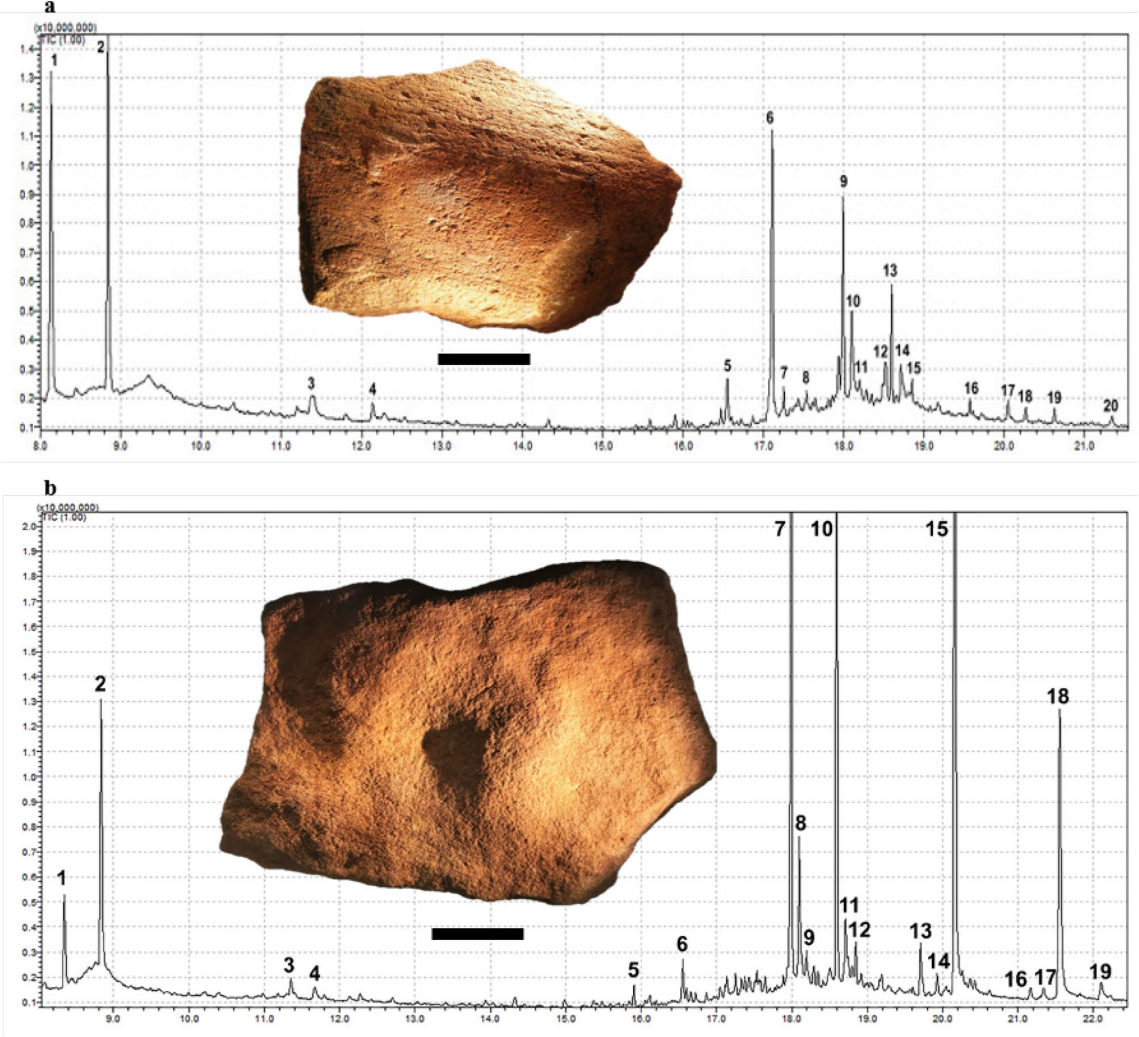
GC–MS chromatographs of seed grinding/pounding tools from Phase 2. (**a**). Detected molecules in extractions sampled from GS73: 1) unidentified carbohydrate; 2) unidentified carbohydrate; 3) degraded amino acid; 4) nonanoic acid; 5) contamination from plastics; 6) methylcyclodecane; 7) dodecanoic acid; 8) pentadecanol; 9) hexadecenoic acid; 10) ascorbic acid; 2,6-dihexadecanoate; 11) 3,7,11-trimethyl-1-dodecanol; 12) pentadecanol; 13) 16-methyl heptadecanoic acid; 14) octadecanoic acid, 2-hydroxy-1,3-propanediyl ester; 15) 8-octadecenal; 16) unidentified terpenoid (similar to farnesan); 17) unidentified terpenoid; 18) contamination from plastics; 19) 10-methyl-nonadecane; 20) 6-methyl-octadecane. (**b**). Detected molecules in extractions sampled from GS75: 1) unidentified carbohydrate; 2) unidentified carbohydrate; 3) 2-methyl-2-phenyl-oxirane (epoxy-Cumene); 4) degraded fatty acid; 5) contamination from plastics; 6) contamination from plastics; 7) hexadecenoic acid (palmitic acid); 8) ascorbic acid, 2,6-dihexadecanoate; 9) unidentified long chain fatty acid; 10) octadecanoic acid; 11) hexadecanoic acid, 1-(hydroxymethyl)-1,2-ethanediyl ester (dipalmitin glycerol); 12) pentadecanol; 13) 2,2'-methylenebis[6-(1,1-dimethylethyl)-4-methyl-phenol (2,2'-methylenebis[6-tert-butyl-)p-cresol); 14) 2,3-dihydroxypropyl hexadecanoate (Monopalmitin); 15) hexadecanoic acid, 2-hydroxy-1-(hydroxymethyl)ethyl ester (2-mono-palmitin); 16) unidentified long chain fatty acid; 17) unidentified long chain fatty acid; 18) octadecanoic acid, 2,3-dihydroxypropyl ester (1-mono-stearin);19) unidentified long chain fatty acid.

GC-MS analysis of extracted residue samples from 97 of the analysed grinding stones identified more than 200 chemical compounds, including fatty acids, aromatic carbons, amino acids, proteins (including porphyrin structures and blood components), carbohydrates and bioactive compounds (Supplementary Material Section 8; Supplementary Table S7). Plant-derived compounds were detected on 47 grinding stones across all occupation phases. These included 26 artefacts with residues attributed to the processing of seeds, nuts, tubers, roots, leaves, wood or fruit, based on the combination of compounds present, including the relative ratios of bioactive compounds, fatty acids and aromatic hydrocarbons (Supplementary Table S7). Compounds such as monoglycerides and certain unsaturated fatty acids are naturally found in seed oils and have been identified on a number of artefacts, including GS73 and GS39 from Phase 2, both of which have usewear consistent with seed processing (Figs. 7a–b, 3q–v). Another artefact from Phase 2, GS75, which has a pitted central depression, had a number of plant-derived compounds detected within its central depression, including a vitamin C fatty ester (ascorbic acid), various antioxidants, sterols, fatty acids and glycerides (Fig. 7b; Supplementary Table S7). The wear on this artefact in addition to the compounds detected from within its central depression are indicative of the pounding of seeds, nuts and/or fruits.

Five grinding stones (GS8, GS16, GS27, GS35 and UPGS6) from Phases 7, 5, 4 and 3, had other plant-derived compounds that were distinctive of bioactive alkaloids and indicate the possible processing of toxic or narcotic plants, possibly with hallucinogenic effects (Supplementary Table S7).

The presence of animal-specific compounds (including amino acids, animal fats and blood protein) were detected on three grinding stones (GS3, UPGS21, UPGS32) from Phases 5–3, respectively (Supplementary Table S8). Other potential animal-derived compounds (e.g., azelaic acid) that were also detected on other grinding stones were not considered to indicate animal processing as these compounds may also derive from plants. Residues from modern handling (including hexadecanoic and octadecanoic acids) and those acquired during storage (phenolic acids from plastics) were detected on 24 artefacts (Supplementary Table S7).

## Discussion

We based our interpretation of tool function on multiple lines of evidence including grinding stone morphology, usewear and residues. Our combined analyses indicates that the grinding stones from Madjedbebe were used for a diverse range of tasks, including the processing of plants (n = 60, including seeds, n = 17), animal tissue (n = 4), red pigments (n = 17) and the direct striking/filing of stone (n = 5) (Table 2).

**Table 2:** Number and percentage of grinding stones from each phase with assigned functions and likely processed materials.

Worked materials	Plant	*Seed*	*Other plant*	Animal	*Bone*	*Meat/skin*	Pigment	Stone	*Hammerstone (stone knapping)*	*Whetstone (axe grinding)*	Metal	Unknown	Multiple	Total no. of analysed grinding stones (including fragments) from each Phase
Phase 7	**4**	3	1	**0**	0	0	**0**	**1**	0	1	**1**	**0**	**1**	**5** (~ 5%)
Phase 6	**3**	2	1	**0**	0	0	**1**	**0**	0	0	**0**	**0**	**0**	**4** (~ 4%)
Phase 5	**8**	0	8	**2**	0	2	**3**	**1**	1	0	**0**	**4**	**3**	**15** (~ 14%)
Phase 4	**24**	8	16	**2**	1	1	**5**	**1**	1	0	**0**	**6**	**5**	**33** (~ 32%)
Phase 3	**9**	2	7	**0**	0	0	**2**	**1**	0	1	**0**	**6**	**2**	**16** (~ 15%)
Phase 2	**11**	2	9	**0**	0	0	**6**	**1**	0	1	**0**	**13**	**2**	**29** (~ 30%)
Phase uncertain	**1**	0	1	**0**	0	0	**0**	**0**	0	0	**0**	**1**	**0**	**2** (~ 2%)
TOTAL	**60**	17	43	**4**	1	3	**17**	**5**	2	3	**1**	**30**	**13**	**104**

Note that individual grinding stones may have multiple uses.

Significant values are in [bold].

Plant processing was the most common task, which was attributed to 60 grinding stones across seven occupation phases, including eight artefacts from Phase 2 (Table 2; Supplementary Table S8). The grinding stones were used to process both soft and hard seeds, starchy plants, and other softer plant materials such as leaves and geophytes. Seventeen of the 60 grinding stones (~ 28%) had usewear and/or residue traces consistent with seed processing (Table 2), including at least two artefacts (GS39 and GS73) from Phase 2, though no starch was identified (Fig. 3). These two artefacts provide the earliest evidence for seed grinding outside of Africa^57^ and are the earliest known seed grinding implements in Australia, predating other Pleistocene examples from Cuddie Springs^58^ and Lake Mungo^56^. Evidence for the use of grinding stones for plant-food processing outside of Africa does not exist elsewhere until about 30 ka ago, as documented in Upper Palaeolithic sites in Europe^1,59–61^, China^62,63^ and the Levant^64^. Consequently, the identification of plant processing at Madjedbebe is of global significance and indicates that such implements were part of the toolkits of early modern human societies^65,66^. Grinding stones used for this purpose are relatively more common during Phases 2 and 4 (Table 2), correlating with drier periods of occupation and reflecting the exploitation of lower ranked resources^50^.

Interestingly, starch grains were not recovered from some grinding stones that had usewear traces diagnostic of plant processing (e.g., GS 16, UPGS26) (Fig. 3g–i, n–p; Supplementary Table S5). The apparent lack of starch grains on these and other grinding stones from Phases 3–7, which have usewear consistent with grinding seeds, is difficult to explain, but may be linked to their location relative to the rock shelter wall and sediment chemistry. It may also indicate that these grinding stones were used to process non-starchy plants with high silica contents or that the residues associated with seed grinding may not have survived the most recent use.

Pigment processing was the second most common task after plant processing, identified on 17 grinding stones, and accounting for just over 16% of the total analysed assemblage. These implements, used to process red haematite to produce a powder, were most common in occupational phases spanning the Pleistocene, with 13 of them identified in Phases 4–2 (Fig. 5) (Supplementary Tables S8). The higher frequency of ground pigments and grinding stones used for processing them during certain phases of occupation could indicate “pulses” of artistic activity (see^67^), which is also reflected in the abundance of ground ochre and haematite pieces from the site^39^. Unfortunately, pulses in pigment extraction in Phases 4–2 have no known associated artistic styles since they far exceed the antiquity of dated artistic styles in western Arnhem Land, although they certainly predate the Holocene-aged Northern Running Figures, Estuarine and X-Ray art styles^68–71^. Changes in artistic style likely reflect broader changes in economy, social life and ideology, as well as occupational intensity at the site^59^. However, the relatively higher proportions of grinding stones used for pigment processing in Phase 2 (compared with other tasks recognised in Phase 2) could be the outcome of residue preservation bias—that is, the inorganic iron oxide minerals resulting from the processing of pigments are more favourable to preservation than organic residues that result from the processing of plants, seeds and animals. Thus, it appears that pigment processing was more common relative to other grinding activities involving the processing of organic materials at this time. Indeed, organic residue preservation was much lower on artefacts from Phase 2 compared with grinding stones from more recent Phases—with lower frequencies of visible organic tissues and fewer detected molecules on Phase 2 artefacts. In some cases, usewear was diagnostic of function but residues were not recovered.

Stone working was recognised on five grinding stones and included three that were used to file stone (two whetstones—UPGS39 from Phase 7—a modern (post-European contact) mudstone brick that was used for sharpening metal and stone axes; and GS79 from Phase 2—a thin, flat, sandstone slab with negative flake removals around the outer edges (Figs. 2e, 4a–c, j–l); and one larger, stationary stone, GS38) and two upper stones (GS18, Phase 4 and GS7, Phase 5) with traces that indicate they were used to buff and knap stone in addition to working plants (Supplementary Table S8). Other evidence for stone grinding included the presence of at least 10 complete or near complete edge-ground hatchets in addition to numerous flakes from their surfaces and edges. Evidence for the manufacture and maintenance of edge ground hatchets during Phase 2 included the presence of a complete axe^39^, a deliberately shaped whetstone with usewear consistent with stone grinding (GS79, Fig. 4j–l) and numerous ground-stone flakes that were removed from their surfaces and edges. These ground stone implements from Madjedbebe provide the earliest evidence for the manufacture and maintenance of edge-ground hatchets in the world^1^, and may predate earlier examples of axe fragments from Carpenter’s Gap 1 and Carpenter’s Gap 3 in Western Australia by up to 20 ky^31,72^.

Animal processing implements were uncommon, with evidence limited to four artefacts, just under 4% of the total analysed grinding stone assemblage. These included three grinding stones that were used to process animal flesh (meat and/or skin) (GS3 and GS9, both Phase 5; and UPGS17, Phase 4) and one that was used to process bone (UPGS21, Phase 4, Fig. 4d–i). Usewear diagnostic of animal processing was rare with only one grinding stone displaying wear indicative of this activity (UPGS21). The evidence for the processing of animal material was mostly reflected by the presence of residues detected via biochemical analyses or visually identified from within residue extractions. Visually identified animal residues include bone, collagen and highly degraded hair fibres. GC–MS analysis also detected animal fats, amino acids and degraded blood molecules on two of the grinding stones (GS3, UPGS21). Usewear and other residues consistent with processing plant material and red pigment were also documented on all four artefacts, indicating they were used to process multiple materials. We suspect that these multifunctional tools primarily functioned in the processing of plants/pigments and that they were used opportunistically to process animal material.

Generally, grinding stones were most common in Pleistocene Phases 2 and 4 and Holocene Phases 6 and 7. Greater abundance mostly corresponds with drier phases (Table 1), although Florin et al.^73^ conclude from a study of charred pandanus (*P. spiralis*) δ13C records from the site that rainfall in the vicinity of Madjedbebe may have remained relatively high, even during the driest phases, consistent with arguments that the region may have seen more intensive occupation during the drier periods of MIS 4 and 2^74^. Grinding stones in Phases 2, 4, 6 and 7 are associated with increases in plant food diet breadth as reflected by the archaeobotanical remains from the site, some of which involved grinding and pounding, including seeds, hard nuts and some fruits^50^. Peaks in grinding stone use also correspond to peaks in occupational intensity and a rise in the discard of exotic raw materials, greater overall lithic reduction, and discard of worked ochre, all of which may be indicative of greater occupational intensity during these drier phases. Greater use of grinding stones during such times may reflect a broadening of the diet as high ranked resources became depleted during longer or more frequent visits. In this way, grinding stones acted as ‘site furniture’ for processing lower ranked foods such as hard nuts and seeds (that require more intensive processing) at a location that was visited more frequently and predictably during drier times^23,75^. Animal materials were also processed on grinding stones for the first time during the LGM (Phase 4), perhaps for reasons of nutritional stress to reduce faunal wastage, mirroring the practice of extensive bone processing for protein extraction documented in arid economies in Central Australia in the recent past^76,77^.

## Conclusion

The grinding stone assemblage at Madjedbebe provides the first extensive evidence of the earliest processed food diets in Sahul, showing a high rate of multifunctionality and a diverse range of tasks, from sharpening edge-ground axes to processing seeds, soft and hard plants, pigment extraction and the pulverising of animal tissues. Residues relating to seed processing were only documented on one grinding stone from Phase 2; however, the usewear on some of the other Phase 2 stones was highly distinctive, and the stone morphology is characteristic of Australian millstones used in sustained back-and-forth grinding. Grinding stone use and abundance also changes through time, with pigment processing best represented in the earliest phase of intense occupation, and plant processing most abundant during the LGM, a second phase of intense occupation, as diets broadened again to include lower ranked plant foods that required processing. Animal tissues were processed on grinding stones for the first time at the site during the LGM. The Madjedbebe grinding stones provide the first glimpse of a heavy investment in rich and varied grinding technologies in the Pleistocene and demonstrates the highly innovative nature of the first Aboriginal inhabitants of Sahul.

### Methods

#### Usewear

Diagnostic usewear features resulting from the grinding of specific materials were documented on experimental grinding implements made from Australian sandstones of varying hardness^38^ and form the basis of our usewear reference library that enabled interpretations of tool function to be made (Supplementary Material, Section 2). Microscopic usewear was documented under low magnification using an Olympus SZ61 stereo-zoom microscope with an external fibre optic, 150-Watt halogen light source (Olympus LG-PS2); and under higher magnifications using an Olympus BX-51 reflected light microscope with vertical incident light (brightfield and darkfield) with objective lenses of ×50, ×100, ×200 and ×500 and polarizing filters. Larger artefacts that could not fit under the microscope were sampled for usewear using PVS (polyvinyl siloxane) compound (President® Light Body) that was subsequently examined under the Olympus BX-51 vertical incident light microscope.

#### Residue extraction and staining

Residue samples were extracted from the used and unused surfaces of each grinding stone using distilled water and/or a tri-solvent mixture of acetonitrile, ethanol and distilled water. Residues samples (other than starch) were extracted using an adjustable pipette and a disposable nylon pipette tip. Extracted residues were prepared by mounting 5–15 µL of the residue mixture on a clean glass slide (cleaned with ethanol or acetone) and secured with a clean glass cover slip. Slides were examined with an Olympus BX-51 metallographic microscope and images were captured with an Olympus DP72 Microscope Camera. A selection of animal-specific (Orange-G, Rhodamine B, Safranin) and plant-specific (Congo Red, Iodine Potassium Iodide, Methylene Blue, Phloroglucinol) stains were selected to confirm the presence of animal or plant material (Supplementary Information, Section 5). Ten to forty microlitres of staining solution was added to selected slides and left for at least 10 Min to ensure adequate development time and then rinsed out with distilled water. Slides where re-examined using the transmitted light microscope to assess any positive colour changes in the constituent residue material.

#### Starch analysis

Selected grinding surfaces and grinding stone fragments were sampled by partial or complete immersion in an ultrasonic bath. Artefacts were partially or completely immersed in distilled water and sonicated for 2 Min. The residue sample was then centrifuged to concentrate the sample. Starch and any phytoliths were isolated with heavy liquid (sodium polytungstate, Specific Gravity 2.35) and mounted in water. Slides were scanned using a Zeiss Axioskop2 brightfield transmitted light microscope fitted with Nomarksi optics. All starch grains were photographed using a Zeiss HrC digital camera and Zeiss Axiovision software. Individual grains were digitally traced and archived. Hilum position, presence/absence of lamellae and fissures, open or closed hilum and presence of faceting were noted.

#### Absorbance spectroscopy

Absorbance spectra of extracted residues were measured from dried residue samples that were subsequently diluted with distilled water as needed. Two microlitres of liquid solution were placed in a Take 3TM plate, ensuring that no particulate material was present within the sample that may cause scatter within the scan. Absorbance spectra were then measured between 200 nanometres (nm) and 900 nm using an EpochTM MultiVolume Spectrophotometer System (Biotek) at 2 nm increments. The data were collected and analysed using Gen 5 software (Supplementary Information Section 7).

#### Biochemical testing

Biochemical tests including the Bradford Assay, Copper triethanolamine diphenyl-carbazide (cf. “Falholt” test of Fullagar et al. 2015); Iodide-Potassium-Iodine; Hemastix® and the Diphenylamine and the Phenol-Sulphuric Acid test, were undertaken on residue mixtures extracted from the ground and unground artefact surfaces using either water or tri-mixture solvent of acetonitrile, ethanol and distilled water (Supplementary Information Section 7). Each test was performed on a small portion of sample (< 5 μL) and observed for a subsequent reaction, indicated by a specific colour change. Positive reactions were identified using the EpochTM Multi-Volume Spectrophotometer System (see above) following a set of standard measurements using blood protein, corn starch, cooking oil and a combination of sucrose and glucose. The readings from these measured standards were considered the minimum value for the detection of proteins, starch, fatty acids and carbohydrates, respectively. To assess the possibility of environmental contamination, accompanying sediment samples were also tested.

#### Gas-chromatography Mass-spectrometry

Desiccated residue samples from the water and tri-mixture solvent extractions sampled from artefacts collected during the 2012 excavations were prepared for GC–MS by adding 500 μL of acetonitrile to sample tubes for 24 h. The acetonitrile was then removed and placed into a separate glass vile ensuring that no particulate material was present. Before sealing, all oxygen was removed from the glass vial by purging the vial with nitrogen gas and sealing it with aluminium caps. Selected artefacts recovered during the 2015 excavations (n = 6) were sampled for residues with a chloroform/methanol (3:1) solution. GC-MS analysis was performed using a Varian model 450 gas chromatograph coupled with a Varian model 300-MS quadrupole mass spectrometer fitted with FactorFourTM capillary column (VF5ms, 30 m × 0.25 mm ID, DF = 0.25 μm), following the methods described by Crowther et al. (2015:380). The chemical compounds recovered from each residue mixture were identified following the characterisation of their ion spectra and the ionisation peaks (e.g., the molecular ion, M+ peak, M+1 peak and the various ionisation peaks M-15 peaks), using Varian MS Workstation Version 6 and the NIST98 Mass Spectral Database (National Institute of Standards and Technology). Compounds were then cross-referenced with published data to enhance taxonomic identification (Supplementary Information Section 8).

## Supplementary Information

Supplementary Information.

## Data Availability

The datasets used and/or analysed during the current study available from the corresponding author on reasonable request.
