# Supplementary material for: 65,000-years of continuous grinding stone use at Madjedbebe, Northern Australia
Source: Sci Rep. 2022 Jul 11;12:11747. doi: 10.1038/s41598-022-15174-x (PMC9273753; doi:10.1038/s41598-022-15174-x)
Supplement: Supplementary file 1 — Supplementary Information. [file 41598_2022_15174_MOESM1_ESM.pdf]

65,000-years of continuous grinding stone use at Madjedbebe, Northern Australia

Elspeth H. Hayes[1,2*], Richard Fullagar[2,3,4], Judith H. Field[5], Adelle C.F. Coster[6], Carney Matheson[7], May Nango[8], Djaykuk Djandjomerr[8], Ben Marwick[9], Lynley A. Wallis[10], Mike A. Smith[11,12], Chris Clarkson[3,13-15*].

1. MicroTrace Archaeology, PO Box 102, Wollongong, NSW 2520, Australia
2. Centre for Archaeological Science, School of Earth, Atmospheric and Life Sciences, Faculty of Science, Medicine and Health, University of Wollongong, NSW 2522, Australia.
3. School of Social Sciences, University of Western Australia.
4. History and Archaeology, College of Humanities, Arts and Social Sciences Flinders University.
5. School of Biological, Earth and Environmental Sciences, The University of New South Wales, NSW 2052 Australia
6. School of Mathematics and Statistics, University of New South Wales, Sydney, NSW 2052, Australia
7. School of Environment and Science, Griffith University, Nathan, QLD 4111, Australia
8. Gundjeihmi Aboriginal Corporation, Jabiru, NT 0886, Australia
9. Department of Anthropology, University of Washington, Seattle, Washington 98195, USA.
10. Griffith Centre for Social and Cultural Research, Griffith University, Brisbane, QLD 4111, Australia.
11. College of Humanities, Arts and Social Sciences, Flinders University, Adelaide SA 5042, Australia.
12. Centre for Historical Research, National Museum of Australia, Canberra, Australian Capital Territory 2601, Australia.
13. Department of Archaeology, Max Planck Institute for the Science of Human History, Kahlaische Strasse 10, Jena, 07745, Germany.
14. Australian Research Council (ARC) Centre of Excellence for Australian Biodiversity and Heritage, University of Wollongong, Wollongong, NSW 2522, Australia.
15. School of Social Science, University of Queensland, St Lucia, Qld 4072, Australia

*Corresponding authors: Elspeth Hayes, ehayes@mtrace.com.au, Chris Clarkson c.clarkson@uq.edu.au

This document includes:

Supplementary Materials

SUPPLEMENTARY TEXT:

Section 1: Published site chronology……………………………………………………......4

Section 2: Grinding stone morphological classes …………………………………………7

Section 3: Functional analysis and usewear/residue reference libraries …………………...12

Section 4: Usewear on the Madjedbebe grinding stones …………………………….……15

Section 5: Microscopically visible residues on the Madjedbebe grinding stones & staining of extracted residues ……………………………….……….……….....................21

Section 6: Starch grain analysis ………………………………………………………….… 30

Section 7: Biochemical tests & Absorbance spectroscopy ……………………………..… 33

Section 8: Gas-Chromatography Mass Spectroscopy (GC-MS) ………………….…….… 49

Section 9: Functional interpretations ………………………………………………….….. 80

References ……………………………………………………………….…………….... 83

SUPPLEMENTARY TABLES:

Table S1: Grinding stone details ……………………………………………..……..…… 9

Table S2: Usewear on experimental tools ……………………………………….…….. 14

Table S3: Usewear on Madjedbebe grinding stones ………………………………….……. 16

Table S4: Residues documented on Madjedbebe grinding stones adhering to the tool surface and in water extractions ………………………………………………………………..…… 25

Table S5: Starch grain counts from ultrasonicated residue samples ………….…….….. 32

Table S6A: Measurement standards for biochemical tests ……………………….....…… 34

Table S6B: Biochemical tests and absorbance spectra ………………………………..… 38

Table S7: Compounds detected on Madjedbebe grinding stones with Gas-Chromatography Mass Spectroscopy …………………………………………………………………….… 50

Table S8: Summary of grinding stone function(s) by Phase ………………..…………… 80

SECTION 1: SUMMARY OF PUBLISHED SITE CHRONOLOGY

In 2017, high-resolution sampling of quartz-rich sediments for single grain OSL dating, combined with advances in OSL measurement technology and dating procedures, resulted in publication (Clarkson et al. 2017) of 50 more accurate and precise age estimates than those previously published for Madjedbebe. OSL dating gives an estimate of the time since mineral grains were last exposed to sunlight. Full details of OSL and radiocarbon dating at Madjedbebe are provided in Clarkson et al. (2017) and Florin et al. (2021).

A ~2 m-thick archaeologically sterile sand unit (4.6–2.6 m below surface) underlies the first certain evidence for human occupation of the site, designated as Phase 1. The deepest samples published in Clarkson et al. 2017 were from ~2.9 m below surface giving a start date for this phase of 80.2 ± (7.2, 9.0) kyr ; the first and second error terms are the modelled age uncertainties at 95.4% probability, excluding and including the total systematic error, respectively. Sedimentation, however, commenced at a depth of ~4.6 m below surface. The revised age estimates for the samples collected for TL dating from the basal deposits by Roberts et al. (1990a) and Roberts and Jones (1994) suggest that the sand apron started forming 121 ± 18 kyr (1σ) ago, around the time of the last interglacial. The end date of this phase was calculated to be 71.0 ± (5.6, 7.3) kyr, which corresponds to a mean sediment accumulation rate of 4.0 ± 0.6 cm/kyr between 4.6 and 2.6 m depth. The uncertainty on this rate estimate is expressed at 68.2% probability and determined from the random errors only. This end date for Phase 1 also coincides with the transition from Marine Isotope Stage (MIS) 5 to 4, which may be significant as a palaeoclimatic control on the sand apron accumulation. Low densities of artefacts occur between about 3 and 2.6 m depth, but the first dense artefact band (Phase 2 in the Bayesian age model) occurs between 2.6 and 2.15 m below surface. For this band, we obtained start and end ages of 65.0 ± (3.7, 5.7) kyr and 52.7 ± (2.4, 4.3) kyr, respectively, giving a mean sediment accumulation rate of 4.1 ± 0.8 cm/kyr over this depth interval. The latter was calculated from the modelled estimate of phase duration and the corresponding total random uncertainty at 68.2% probability; the same procedure was used for all subsequent phases. Phase 3 represents an archaeological unit, but with reduced artefact abundance. The modelled start age for this unit is of 51.6 ± (2.4, 4.2) kyr, which is statistically consistent with the end age of Phase 2, suggests no significant time gap in sediment deposition. Phase 3 ended 28.1 ± (2.1, 2.8) kyr ago, indicating a much slower rate of sedimention over this period (2.6 ± 0.2 cm/kyr). The start of Phase 4 represents an increase in lithic abundance, beginning at 26.7 ± (2.2,

2.8) kyr ago and ended 13.2 ± (1.0, 1.3) kyr ago with a mean accumulation rate of 4.4 ± 0.4 cm/kyr during the lead up to and through the last glacial maximum. There appears to be a hiatus of 3.6 ± 0.9 kyr between Phases 4 and 5, coupled with a noticeable drop in artefact abundance associated with the latter, 25 cm-thick sedimentary unit (0.95–0.70 cm below surface). The modelled start and end ages for Phase 5 are 9.7 ± (0.8, 1.1) and 8.0 ± (1.0, 1.1) kyr, respectively, resulting in a mean sedimention rate of 15.7 ± 7.4 cm/kyr. Phase 6 represents a pulse of high lithic abundance in a 35 cm-thick band, which started 7.1 ± (1.0, 1.1) kyr ago. Phases 6 and 7 are represented by only four relatively imprecise OSL ages, so they are not well constrained by OSL dating. However, a number of ^{14}C ages from Phase 7 suggest that this unit accumulated within the last 600 years. Further radiocarbon ages presented in Florin et al. (2021) confirmed that Phase 7 formed within the last 600 years and that a hiatus exists between Phases subsequently divided into Phase 7a, which formed 600 years ago, and Phase 7b, which formed in the last 150 years.

Many of the single-grain OSL equivalent dose (D_e) distributions include some grains with smaller and higher D_e values than those of the majority of grains, which we interpreted as evidence of small-scale mixing of the deposit. Furthermore, some of the OSL samples were collected in 5-cm-diameter tubes, which will also result in some time-averaging (approximately 1,250 years at an average sedimentation rate of about 4 cm kyr−1). For this reason, OSL age estimates were modelled as 'Phases' within the Bayesian framework, which assumes that the age estimates are unordered and uniformly distributed, so that any mixing within an archaeological phase (e.g., Phase 2) will not influence the model. This assumption was supported by the presence of stone artefact refits that showed horizontal and lateral movement within a phase but not between phases (Clarkson et al., 2017). The age of artefacts within a Phase can, therefore, be interpreted as falling somewhere between the modelled start and end ages of a Phase. For example, for Phase 2, the earliest and latest possible age estimates for human activity are 65.0 ± 3.7 kyr and 52.7 ± 2.4. It is, however, not possible to ascertain whether occupation occurred continuously throughout the entire unit, or whether it was clustered towards one end of the age range or close to the middle. Further confidence in the veracity of the age estimates were obtained from a series of interlaboratory comparisons, consistent age estimates obtained for both the 2017 and 1989 samples and from across several spatially distinct areas, and agreement with ^{14}C ages down to a depth of ~1.5 m presented in Clarkson et al. (2017) and Florin et al. (2021).

Contention around the age of Madjedbebe following publication has centred around whether artefacts could have moved down through sandy sediments over time as a result of bioturbation

from termites, trampling, or seasonal wetting and drying (Allen 2017; Allen & O'Connell 2020; O'Connell et al. 2018; Williams et al. 2021). As pointed out in the original study and subsequently (Clarkson et al. 2017, 2018), mass movement of artefacts through the profile are unlikely due to: (1) the existence of tightly packed rubble in the front several metres of the excavation; (2) the distinctive nature of Phase 2 raw materials, including artefacts that are technologically distinct from the later phases; (3) an absence of termite related features or clay particles associated with termite galleries; (4) low dispersion in the ages of individual grains from Phase 2, indicating little post-depositional mixing; (5) trampling experiments in sediments from the site which indicate small scale movements of artefacts; (6) no size-sorting of artefacts or sediments as expected of termite bioturbation; (7) peaks in magnetic susceptibility indicating heightened human activity and burning associated with pulses in stone artefact discard incompatible with widespread mixing; (8) the presence of intact cultural features in Phase 2 including hearths; (9) artefact refits within but not between Phases 2 and 4; and (10) artefact associations such as ground ochre wrapped in mica sheets (Clarkson et al. 2017, 2018; Marwick et al. 2017; Smith et al. 2020, 2021). O'Connell et al. (2020) most recently speculated that fine particles may have moved through the sequence by termites without altering the position of sand grains, thus preserving low over-dispersion in OSL ages from the site. But if this were true, then the association between artefacts and dated sand grains would also be preserved. Consequently, while all archaeological sites undergo some degree of post-depositional alteration, there is currently no sustainable evidence upon which to dismiss the Madjedbebe chronology and its associated artefactual sequence.

SECTION 2: GRINDING STONES MORPHOLOGICAL CLASSES

2.1 Introduction

Ground-stone artefacts (i.e., stones that possess grinding or abrasive wear) include all stone items that have been either intentionally modified to a specific form through grinding (i.e., manufacture-ground); or used in the grinding, pounding or filing (abrading) of other materials (i.e., use-ground) (Adams 1994: 17; Odell 2004: 74–85). Manufacture-ground tools include both ornamental and utilitarian tools, such as polished stone, vessels and beads, as well as ground-edge axes, adzes and bowls. Use-ground stones include all utilised grinding dishes, portable hand stones, stone "files" and large bedrock grinding patches. Functional analysis of use-ground stones (hereafter referred to as "grinding stones") involving the documentation of tool stone morphology, usewear and residues, can potentially indicate past grinding activities. Thus, the study of these implements from Madjedbebe in northern Australia provides a rich and unique source of archaeological evidence for the utilisation of plant, animal and other resources, since initial colonisation**.**

We distinguish two classes of grinding stone implements: (1) filing stones, which are used to process a material through direct contact (cf. "abraders" or "polishers" as described by Adams 1993: 64, 2002: 143–5; and Hamon 2008: 1504); and (2) coupled stones, which are used in conjunction with another stone to process an intermediate material (Table S1).

2.2 Coupled stones

Coupled stones often include one large, stationary "lower" grinding stone and a smaller, active "upper" stone that is held in the hand(s) (Odell 2004: 78; Wright 1994: 239), which are used together to process an intermediate material. These artefacts may be previously unmodified stones, selected for size and shape, or they may have been manufactured, sometimes by grinding and flaking. The active stone is used in either a back-and-forth, rotary, or pounding motion to grind or otherwise process the material on the stationary basal stone. Hammerstones and other percussion/pounding tools such as mortars, anvils and pestles, may also be classified as coupled stones, as processing involves the contact of two stones during pounding and crushing activities (De la Torre 2013: 313; Odell 2004: 79; Wright 1994: 239). These tools often show traces of crushing, pounding, grinding and battering (Kraybill 1977: 493). Mortars are bowls or flat-bottomed slabs with circular or oval depressions forming the concave receptacle that holds the material to be processed (Kraybill 1977: 491; McCarthy 1976: 63). Pestles are the accompanying

pounding/grinding/crushing tools, which are usually fist-sized and rounded in section but can be elaborately carved. Pestles can simply be specially selected water-worn cobbles, and typically have crushing wear from impact with the mortar. Mortars and pestles are typically used to crack and pound hard materials such as seeds and nuts, which often contain a hard outer shell (e.g., Goren-Inbar et al. 2002; Peterson 1968). These implements may also be used to process other materials such as bone, shell and pigment (e.g., Liu et al. 2010; Peterson 1968; Van Peer et al. 2003).

2.3 Filing stones

Filing stones are used singly to process and shape a variety of materials such as stone, bone, wood, shell, ivory and ochre. Filing stones may exist as portable stone "files" (e.g., whetstones [hand-held implements used for grinding and sharpening stone axe blades, chisels and knives, McCarthy 1976: 60–61]; fish-hook files), as fixed features on rockshelter walls, and as large boulders and as outcropping bedrock, where they often occur in the form of circular or ovate grinding grooves (e.g., axe-grinding grooves). It is important to note that filing and coupled stones are not mutually exclusive; these tools may sometimes be used interchangeably to process multiple materials. For example, hammerstones and mullers can be used to polish wooden artefacts, and to process other materials on different lower stones (McCarthy 1976: 61). In our analysis, we refer to grinding stones as either a polishing, filing or coupled stone with reference to their design, dominant mode of use and most recent use (Table S1).

2.4 Distinguishing classes of grinding stones

Distinguishing between use-ground and manufacture-ground implements involves careful evaluation of the artefact design, manufacture traces, usewear and residues. Manufacture-ground implements such as ground-edge hatchets, axes and knives will typically display a bevelled edge with a finely abraded surface. Grinding wear on use-ground implements, on the other hand, will occur on the contact surface where grains have been altered or removed following contact with the processed material or upper stone. When only small fragments of a broken tool have survived, it may be very difficult to distinguish between use-ground and manufacture-ground traces.

Determining whether a use-ground stone should be classified as a coupled or filing stone depends on the artefact morphology and surface features recognised at various magnifications. For example, stones used for filing wood, shell or bone typically display a flat or concave cross

section with no traces of stone-on-stone working—unless they were also used to sharpen or repair stone axes. In contrast, coupled stones such as mortars and pestles, and other lower and upper stones, will display stone-on-stone wear and will typically be concave and convex in section, respectively.

Broken fragments of lower stones have problematic morphologies with concave-convex portions. Although it is usually possible to distinguish between filing stones and coupled stones found archaeologically, the two tool classes are not necessarily mutually exclusive: it is possible to have a grinding stone which was used for multiple purposes, such as an upper or lower stone used as an impromptu filing stone to sharpen a stone axe. Broken pieces are, again, much more difficult to identify.

Table S1: Details of the grinding stones and fragments from Madjedbebe that were examined for usewear and residue traces.

**Artefact ID**	**Spit/Square**	**Phase**	**Stone material**	**No. ground surfaces**	**Shape of ground surface(s)**	**Grinding stone class**	**Complete-ness**	**Mass (g)**	**Length (mm)**	**Width (mm)**	**Depth (mm)**
GS1	D2/5	7	sandstone	1	F	lower	fragment	700	154	83	27
GS2	C3/8	6	sandstone	2	CV/CV	upper	fragment	143	64	51	26
GS3	E1/21	5	sandstone	2	F/F	filing	complete	408	123	87	28
GS4	E1/21	5	sandstone	1	CV	filing/ upper	fragment	323	60	81	40
GS5	D2/21	5	sandstone	1	F	filing	fragment	100	92	66	8
GS6	C2/21	5	sandstone	1	CV	uncertain	fragment	480	98	61	54
GS7	C2/22A	5	quartzite	1	CV	upper/hammer	fragment	84	55	40	28
GS8	D2/8	7	sandstone	2	CC/CV	upper	complete	530	123	55	42
GS9	D2/24	5	sandstone	1	CV	uncertain	fragment	23	33	27	11
GS10	D2/24	5	sandstone	2	CC/F	uncertain	fragment	69	48	34	24
GS13	D2/25A	4	sandstone	2	F/F	uncertain	fragment	168	79	53	35
GS14	D2/26	4	sandstone	3	F/F/F	coupled	fragment	64	41	47	18
GS15	D2/26	4	sandstone	1	F	filing	fragment	91	56	60	16
GS16	D2/26	4	sandstone	2	F/CV	upper	fragment	150	51	51	39
GS18	D3/26	4	quartzite	2	CV/CV	upper/hammer	complete	286	70	56	39
GS19	C2/26A	4	sandstone	1	CV	coupled	fragment	51	30	30	23
GS20	E1/27	4	sandstone	1	CC-F	lower	fragment	34	59	43	5
GS21	E2/28A	4	sandstone	1	F	filing/coupled	fragment	684	112	78	45
GS22	D2/28A	4	sandstone	1	CV	upper	fragment	86	42	58	25
GS23	D2/28A	4	sandstone	2	F/CV	uncertain	fragment	392	87	62	50
GS24	E2/28A	4	sandstone	2	F/CV	upper	fragment	497	109	68	68
GS26	C2/28A	4	sandstone	1	CV	upper	fragment	174	60	56	36
GS27	C1/28	3	sandstone	1	F	uncertain	fragment	208	54	49	52
GS28	C1/28	3	mudstone	1	CV	upper	fragment	171	47	58	34

Artefact ID	Spit/Square	Phase	Stone material	No. ground surfaces	Shape of ground surface(s)	Grinding stone class	Complete-ness	Mass (g)	Length (mm)	Width (mm)	Depth (mm)
GS29	D1/34	3	sandstone	1	F	lower	fragment	703	106	96	40
GS30	D1/34	3	sandstone	1	F	lower	fragment	137	69	44	22
GS31	D1/34	3	sandstone	1	CV	uncertain	fragment	69	425	29	35
GS32	C2-C3/37	2	sandstone	2	CC/F	anvil/lower	complete	1090	241	230	131
GS33	D2/34	3	sandstone	1	F	filing/coupled	fragment	116	62	39	34
GS35	D1/34	3	sandstone	2	CC/F	uncertain	fragment	113	85	52	16
GS36	C1/35	3	sandstone	2	F/F	coupled	fragment	79	76	60	11
GS37	C1/35	2	sandstone	1	CV	upper	fragment	37	46	32	26
GS38	C2/37A	3	sandstone	2	F/F	filing	fragment	7900	263	171	91
GS39	D1/37	2	sandstone	1	F-CV	lower	complete	2635	189	135	73
GS40	D2/37	2	sandstone	1	F	filing	fragment	805	122	118	46
GS41	D2/38	2	sandstone	1	F	filing	fragments	237	75	67	20
GS43	D1/38	2	sandstone	1	F	Filing/coupled	fragment	69	66	54	12
GS44	D2/39A	2	sandstone	1	F	uncertain	fragment	34	58	37	7
GS45	D2/39A	2	sandstone	1	F	uncertain	fragment	31	35	42	19
GS46	D2/39	2	sandstone	2	F/F	uncertain	fragment	50	62	39	20
GS47	D2/39	2	sandstone	1	F	uncertain	fragment	3	31	17	4
GS48	D2/40A	2	sandstone	1	F	uncertain	fragment	330	92	65	38
GS49	C4/29	4	sandstone	1	CV	upper	fragment	91	65	68	19
GS50	C4/45	2	sandstone	1	F	uncertain	fragment	118	87	57	19
GS53	B6/24	4	sandstone	1	CV	upper	complete	233	82	60	24
GS56	B6/31	4	sandstone	2	CC; CC	anvil	complete	4000	250	155	80
GS72	B6/50	2	sandstone	1	CV/U	lower	fragment	no data	no data	no data	no data
GS73	B5/52	2	sandstone	1	CC	lower	fragment	1717	163	120	54
GS74	C6/53	2	sandstone	1	F-CC	lower	fragment	151	77	63	20
GS75	B6/52	2	sandstone	2	CC	anvil	complete	6200	295	177	60
GS79	B6/54	2	sandstone	1	F	filing	complete	718	155	89	20
GS80	BG/56	2	sandstone	1	F/U	lower	fragment	no data	no data	no data	no data
GS82	B6/55	2	sandstone	2	CV	lower	fragment	no data	no data	no data	no data
GS86	B6/63	2	sandstone	1	F/CV	upper	fragment	no data	no data	no data	no data
GS87	C6/66	2	sandstone	1	CV/U	upper	fragment	no data	no data	no data	no data
UPGS1	No data	-	sandstone	3	F/CV/F	upper	fragment	94	46	40	37
UPGS2	C2/5	7	sandstone	1	CV	upper	fragment	3	31	17	3
UPGS3	D2/10	5	sandstone	1	CV	uncertain	fragment	43	62	25	22
UPGS4	D2/16A	5	sandstone	1	CV	filing	fragment	248	92	73	21
UPGS5	B2/21	5	sandstone	1	CV	filing	fragment	36	33	28	23
UPGS6	E2/23	5	sandstone	1	F	uncertain	fragment	11	39	29	10
UPGS7	C2/24A	5	sandstone	1	CV	filing	fragment	14	29	18	19
UPGS9	D2/25	4	sandstone	1	CV	upper	fragment	7	28	26	7
UPGS10	D2/25A	4	quartzite?	1	F	uncertain	fragment	11	43	22	4
UPGS11	D2/25	4	sandstone	1	CV	coupled	fragment	25	54	50	8
UPGS12	D2/26A	4	sandstone	1	F	uncertain	fragment	283	84	56	39
UPGS14	E1/26	4	quartzite	1	F	uncertain	fragments	15	49	37	5
UPGS15	C2/26	4	sandstone	1	F	filing	complete	98	55	43	33
UPGS16	D2/26	4	sandstone	3	F-CC/F-CV/F	upper	fragment	248	83	71	55

Artefact ID	Spit/Square	Phase	Stone material	No. ground surfaces	Shape of ground surface(s)	Grinding stone class	Complete-ness	Mass (g)	Length (mm)	Width (mm)	Depth (mm)
UPGS17	C2/26	4	sandstone	2	FF	uncertain	fragment	46	47	29	25
UPGS18	C2/26	4	sandstone	1	F	filing	fragment	410	76	64	45
UPGS19	C2/26	4	sandstone	1	CV	upper	fragment	192	58	39	52
UPGS21	C2/26	4	sandstone	4	F/F/F/F	filing/upper	complete	219	68	57	380
UPGS22	C2/26	4	sandstone	2	F/F	coupled	fragment	164	56	60	36
UPGS23	D2/28A	4	sandstone	1	F	filing	fragment	76	62	50	19
UPGS24	E3/28	4	sandstone	1	F	filing	fragment	235	70	43	39
UPGS25	C1/29	3	quartzite	1	CV	filing/upper	fragment	25	37	23	21
UPGS26	C3/35	3	sandstone	1	CV	upper	fragment	152	62	28	58
UPGS27	C1/36	3	sandstone	1	F	filing	fragment	14	31	24	10
UPGS28	C2/29A	3	sandstone	1	CV	filing	fragment	198	78	40	50
UPGS29	C2/29A	3	sandstone	1	F	filing	fragment	353	92	67	41
UPGS30	C3/36	3	sandstone	1	CV-F	uncertain	fragment	301	74	73	32
UPGS31	C2/37A	3	sandstone	1	F	filing	fragment	279	118	55	36
UPGS32	C2/37A	3	sandstone	1	F	uncertain	fragment	244	62	54	36
UPGS33	C2/39A	2	sandstone	1	F	filing	fragment	488	109	84	31
UPGS34	C2/39A	2	sandstone	1	F	uncertain	fragment	231	74	73	20
UPGS35	C3/42	2	sandstone	1	F	uncertain	fragment	303	101	54	46
UPGS36	C3/44	2	quartzite	1	F	filing	fragment	82	74	46	20
UPGS37	E2/28A	4	volcanic	1	CV	uncertain	fragment	148	69	56	26
UPGS38	No data	-	sandstone	1	F	filing	complete	3400	253	129	58
UPGS39	C2/3	7	mudstone	5	F/F/F/F/F	filing	fragment	217	98	50	25
L49	C2/5	7	sandstone	1	CV	upper	fragment	539	154	113	18
L52	C3/5	6	sandstone	2	CV/F	upper	complete	1051	117	99	49
L813	D2/23	5	sandstone	1	F	filing	fragment	383	102	82	26
L868	E1/24	4	sandstone	1	CV	uncertain	fragment	13	33	30	11
L894	C2/25	4	sandstone	1	F	uncertain	fragment	14	41	28	6
L1349	D1/32	3	sandstone	1	F	uncertain	fragment	280	70	52	40
R2	C4/4	6	quartzite	1	F	uncertain	fragment	79	92	29	19
R5	E1/21	4	sandstone	3	CV/CV/F	upper	complete	900	115	77	62
R66	E1/17	5	sandstone	3	CV/F/F	lower	complete	8400	280	198	124
R68	E1/18	5	sandstone	2	CC/F	uncertain	complete	642	151	88	29
R69	C2/18	6	sandstone	1	F	filing	fragment	2792	144	108	85
R299	B6/50	2	sandstone	1	F/U	lower	complete	no data	no data	no data	no data
R305	B6/51	2	sandstone	1	F/U	lower	complete	no data	no data	no data	no data

SECTION 3: FUNCTIONAL ANALYSIS OF GRINDING STONES

3.1 Introduction

A range of methodologies are available for determining the function(s) of prehistoric artefacts, including tool-use experiments, ethnographic analogy, tool design and the documentation of traces of use such as usewear and residues. Determining the past function of archaeological grinding stones therefore involves an integrated methodology that includes examination of morphology as well as usewear and residue traces in the archaeological context of discard. The study of wear and residues is particularly important because these traces potentially provide the most detailed indicators of the contact materials on the worked surfaces of the tools. *Usewear* refers to the wear on the edges and surfaces of implements as a result of use, the specific patterning of which may be diagnostic of tool function. The main forms of usewear on ground-stone tools (e.g., grinding stones and hatchet heads) include surface levelling, abrasive smoothing, use-polish, striations and damage to phenocrysts or rock crystals, such as quartz grains in sandstone. Usewear may be macroscopic (visible with the naked eye) but is more often only visible under a microscope. *Residues* refer to the materials that have been transferred and attached to the artefact surface as a result of cultural and non-cultural processes. Of particular interest is the transfer of residues during use (i.e., use-residues), as these can potentially indicate worked material, assuming they are correctly identified. Microscopically visible residues can be categorised under three main groups according to their origin: plant, animal and inorganic. These may be identified by comparison with reference libraries of particles, microscopic cell structures and features (sometimes made visible with biological stains) and by their molecular, chemical and elemental signatures. Because grinding stones are often formed on coarse grained materials such as sandstone, they are usually porous with potential to preserve archaeological residues within the interstitial spaces of the stone matrix. For this reason, residue analysis on sandstone grinding stone implements, such as the majority of those recovered from Madjedbebe, may be particularly insightful for identifying past tool use.

3.2 Experimental reference libraries of usewear and residue traces

Functional interpretations for particular classes of stone tools can be validated using replicative (actualistic) and/or controlled experiments (Hayes et al. 2018a; Marreiros et al. 2020). Replicative experiments are designed to approximate prehistoric conditions to evaluate whether particular tasks can explain, for example, the design and usewear found on archaeological tools. Controlled

experiments, on the other hand, are usually designed to evaluate the role or relative importance of certain aspects of tool function (e.g., duration of use, angle of the working edge relative to the contact material, the effects of foreign or abrasive particles on usewear formation), which may be isolated in replicative experiments or under laboratory conditions. Subsequent documentation of the resulting wear and residue traces can then be used to construct reference libraries of diagnostic usewear and residue traces that can be compared to traces documented on archaeological tools to validate interpretations of tool function.

Reference libraries of usewear on experimental grinding stones from Australian contexts are uncommon and scattered throughout the literature (e.g., Fullagar et al., 2012, 2015, 2017; Hayes, 2015; Hayes et al., 2018b, 2020; Spry et al., 2019), but have indicated that distinct patterns of usewear result from the working of different materials, modes and durations of use, and the stone material (Table S2). In order to create a usewear/residue reference library relevant to the study region, we conducted our own experiments using sandstone collected from near Madjedbebe and processed a range of materials. This research has been fully published by Hayes et al. (2018a) and forms part of the basis for our functional interpretations on the Madjedbebe grinding stones. In summary, we found that stone hardness greatly influences the formation of usewear and that harder sandstones (such as the stone from near Madjedbebe) typically sustain more developed use-polish during use than soft sandstones from other Australian locations, which wear more rapidly. We also found that microscopic usewear was also influenced by the class of grinding tool (upper, lower or filing stone), mode of use (pounding, grinding or filing) and the class of worked material (bone, stone, haematite, seeds, wood). Supplementary Table S2 provides a summary of the main usewear traces documented on our experimental tools. Detailed descriptions and usewear images for each experimental tool comprising our reference library are available in the Supplementary Material of Hayes et al. (2018a).

Table S2: Synthesis of the range of usewear characteristics identified on experimental grinding stones by activity type. *After* Hayes et al. 2018a.

**Activity**	**Low magnification, stereomicroscopy**			**High magnification, vertical incident light microscopy**					
	**Surface levelling**	**Grain rounding**	**Macro striae**	**Use-polish morphology**	**Use-polish brightness**	**Use-polish coverage**	**Use-polish development**	**Fine striae**	**Grain fractures**
Bone filing	minimal–high	moderate–high	common, parallel	smooth-pitted; striated	bright	extensive	high	common, parallel	present
Wood filing	moderate	moderate	parallel	domed-pitted; reticular	bright	extensive	high	parallel	present
Bone pounding	moderate–high	minimal–high	multi-oriented	rough-pitted	dull–bright	localised	weak	multi-oriented	common
Seed pounding	minimal–moderate	moderate–high	uncommon/absent	undiagnostic; reticular	dull–moderate	localised–moderate	weak–moderate	uncommon/ absent	present
Seed grinding	minimal–high	moderate–high	common	reticular	moderate–bright	localised–extensive	weak–very high	multi-oriented	present
Wheat grinding	high	high	multi-oriented	reticular; striated	dull–moderate	localised–moderate	moderate	multi-oriented	present
Stone grinding	high	moderate	parallel	striated	dull–moderate	extensive	high	parallel	common
Axe grinding	high	moderate	parallel	striated; reticular	bright	extensive	high	common, parallel	present
Pigment filing	High	moderate–high	absent	undulating	dull-moderate	localised–moderate	weak-moderate	parallel	present

SECTION 4: USEWEAR ON THE MADJEDBEBE GRINDING STONES

4.1 Introduction

Table S3 lists the macroscopic and microscopic usewear traces documented on the Madjedbebe grinding stones. Macroscopic grinding wear (visible with the naked eye) included levelled, undulating or pecked surfaces comprised of levelled or well-rounded grains, surface striations and visible pits or depressions. Microscopic usewear included abrasive smoothing, use-polish, striations and damage to phenocrysts or rock crystals (quartz grains) within the sandstone. Microscopic usewear was documented using two optical light microscopes: (1) an Olympus SZ61 stereo-zoom microscope with an external fibre optic, 150-Watt halogen light source (Olympus LG-PS2), allowing low magnification observations; and (2 an Olympus BX-51 reflected light microscope with vertical incident light (brightfield and darkfield) with objective lenses of x50, x100, x200 and x500 and polarizing filters. Based on the usewear traces documented on our experimental sandstone grinding tools (Hayes et al. 2018a; Table S2), we recognised usewear traces consistent with the processing of one or more kinds of material (e.g., plants, haematite, stone) on 53 of the 104 grinding stones (~51%). While the remaining 51 artefacts had usewear traces to indicate they were used for grinding/pounding activities, the usewear was not diagnostic of worked material.

Table S3: Usewear documented on grinding surfaces and fragments from Madjedbebe. Key: Shape of grinding surface: CV, Convex; CC, concave; F, flat; U, undulating. Completeness:C, Complete; F, Fragment. Functional class: C, coupled stone; CL, coupled stone (lower); CU, coupled stone (upper); F, filing stone; H, Hammer; ND, not deteremined/uncertain.

Artefact ID	Ground surface #	Shape of ground surface	Low magnification stereomicroscope					Grinding stone type based on morphology	Completeness	High magnification vertical incident light					
			Iron staining	Pecking/ impact damage	Grain levelling	Grain rounding	Macro striae			Use-polish morphology	Use-polish brightness	Use-polish coverage	Use-polish development	Micro striae	Grain fractures
GS1	1 of 1	F	N	N	mod-high	high	Y	CL	F	reticular	bright	extensive	developed	Y	N
GS2	1 of 2	CV	N	N	high	high	N	CU	F	reticular; striated	bright	extensive	developed	Y	N
GS2	2 of 2	CV	N	N	moderate	moderate	N			reticular	bright	extensive	developed	Y	N
GS3	1 of 2	F	N	N	moderate	moderate	Y	FS	C	smooth-domed	dull - mod	localised	weak	N	N
GS3	2 of 2	F	N	N	moderate	slight-mod	Y			undiagnostic	dull	localised	weak	N	N
GS4	1 of 1	CV	N	N	high	high	N	FS	F	undulating	moderate	localised	weak - mod	Y	N
GS5	1 of 1	F	Y	N	absent	slight	Y	ND	F	undiagnostic	dull	localised	very weak	Y	N
GS6	1 of 1	CV	Y*	N	absent	moderate	N	ND	F	undiagnostic	dull	localised	weak	Y	N
GS7	1 of 1	CV	N	N	minimal	moderate	N	CU, H	F	undiagnostic	moderate	localised	weak - mod	N	Y
GS8	1 of 2	CC	Y	N	mod-high	high	Y	CU	C	reticular	bright	extensive	developed	Y	N
GS8	2 of 2	CV	Y	N	mod-high	high	Y			reticular	bright	extensive	developed	Y	N
GS9	1 of 1	CV	Y	N	minimal	mod-high	N	ND	F	undiagnostic	bright	localised	weak	N	Y
GS10	1 of 2	CC	Y	N	minimal	slight-mod	Y	F	F	absent	n/a	absent	n/a	N	Y
GS10	2 of 2	F	Y	N	minimal	slight-mod	Y			absent	n/a	absent	n/a	N	Y
GS13	1 of 2	F	N	N	absent	slight-mod	Y	ND	F	undiagnostic	moderate	localised	weak	N	N
GS13	2 of 2	F	N	N	absent	slight-mod	Y			undiagnostic	moderate	localised	weak	N	N
GS14	1 of 3	F	Y	N	mod-high	moderate	Y	F	F	undulating /reticular	moderate	localised	moderate	Y	N
GS14	2 of 3	F	Y	N	mod-high	moderate	Y			undulating /reticular	moderate	localised	moderate	Y	N
GS14	3 of 3	F	Y	N	mod-high	moderate	Y			undulating /reticular	moderate	localised	moderate	Y	N
GS15	1 of 1	F	N	N	high	high	Y	F	F	undulating	moderate	moderate	moderate	Y	N
GS16	1 of 2	F	N	N	high	mod-high	Y	CU	F	reticular	bright	moderate	moderate	Y	N
GS16	2 of 2	CV	N	N	high	mod-high	Y			reticular	bright	moderate	moderate	Y	N
GS18	1 of 2	CV	Y	N	mod-high	high	Y	CU, H	C	reticular	bright	extensive	w. developed	Y	N
GS18	2 of 2	CV	Y	N	mod-high	high	Y			reticular	bright	extensive	w. developed	Y	N
GS19	1 of 1	CV	N	N	mod-high	moderate	Y	F	F	undulating /reticular	moderate	moderate	moderate	Y	N
GS20	1 of 1	CC-F	Y	N	moderate	moderate	Y	F	F	undulating /reticular	moderate	moderate	moderate	Y	N
GS21	1 of 1	F	N	N	high	high	Y	F	F	undulating /reticular	v. bright	extensive	developed	Y	N
GS22	1 of 1	CV	N	N	high	high	Y	CU	F	undiagnostic	dull	localised	weak	Y	N
GS23	1 of 2	F	N	N	mod-high	moderate	Y	F	F	undulating	moderate	moderate	moderate	Y	Y

Artefact ID	Ground surface #	Shape of ground surface	Low magnification stereomicroscope					Grinding stone type based on morphology	Completeness	High magnification vertical incident light					
			Iron staining	Pecking/ impact damage	Grain levelling	Grain rounding	Macro striae			Use-polish morphology	Use-polish brightness	Use-polish coverage	Use-polish development	Micro striae	Grain fractures
GS23	2 of 2	CV	N	N	mod-high	mod-high	Y			undulating	moderate	moderate	Moderate	Y	Y
GS24	1 of 2	F	Y	N	moderate	moderate	Y	CU, F	F	reticular	bright	moderate	Moderate	Y	N
GS24	2 of 2	CV	Y	N	moderate	moderate	Y			reticular	bright	moderate	Moderate	Y	N
GS26	1 of 1	CV	Y	N	moderate	high	Y	CU	F	undiagnostic	bright	localised	Weak	N	N
GS27	1 of 1	F	Y	N	high	moderate	N	ND	F	undiagnostic	dull	localised	weak	N	Y
GS28	1 of 1	CV	N	N	high	high	Y	F	F	undulating /reticular	bright	moderate	developed	Y	N
GS29	1 of 1	F	N	N	high	high	N	CL	F	reticular	bright	extensive	developed	Y	N
GS30	1 of 1	F	N	N	high	high	Y	CL	F	reticular	bright	extensive	developed	Y	N
GS31	1 of 1	CV	N	N	minimal	high	N	F	F	slightly undulating/ undiagnostic	bright	localised	weak - mod	Y	N
GS32	1 of 2	CC	Y	N	high	high	Y	CL	C	reticular	bright	moderate	moderate	Y	Y
GS32	2 of 2	F	Y	N	high	high	Y			undiagnostic	moderate	localised	weak - mod	N	Y
GS33	1 of 1	F	N	N	high	high	Y	F	F	undulating /reticular	moderate	mod-extensive	moderate	Y	N
GS35	1 of 2	CC	Y	N	minimal	moderate	N	F	F	undulating	bright	moderate	moderate	Y	N
GS35	2 of 2	F	Y	N	minimal	moderate	Y			undulating, slightly reticular	bright	moderate	moderate	Y	N
GS36	1 of 2	F	Y	N	high	high	Y	F	F	slightly undulating/ undiagnostic	moderate	moderate	weak - mod	Y	N
GS36	2 of 2	F	Y	N	minimal	slight	N			undulating, slightly reticular	moderate	localised	weak - mod	Y	N
GS37	1 of 1	CV	N	N	mod-high	high	Y	ND	F	undulating	bright	moderate	mod - developed	Y	N
GS38	1 of 2	F	N	N	minimal	moderate	Y	F	F	undiagnostic	dull	localised	weak	N	Y
GS38	2 of 2	F	N	N	minimal	moderate	Y			undiagnostic	dull	localised	weak	N	Y
GS39	1 of 1	F-CV	Y	N	high	high	Y	CU	C	reticular	bright	extensive	developed	Y	N
GS40	1 of 1	F	Y	N	moderate	moderate	Y	F	F	undulating	moderate	moderate	moderate	N	N
GS41	1 of 1	F	Y	N	moderate	high	Y	F	F	undulating	moderate	moderate	weak	N	N
GS43	1 of 1	F	N	N	high	high	Y	F	F	undulating /reticular	moderate	moderate	weak - mod	Y	Y
GS44	1 of 1	F	Y	N	minimal	slight-mod	N	ND	F	rough-domed	bright	extensive	developed	Y	N
GS45	1 of 1	F	N	N	high	high	Y	ND	F	undiagnostic	moderate	moderate	weak - mod	Y	N
GS46	1 of 2	F	N	N	minimal	moderate	Y	F	F	slightly undulating/ undiagnostic	moderate	localised	weak	Y	N
GS46	2 of 2	F	N	N	minimal	moderate	N			undiagnostic	moderate	moderate	weak	Y	N
GS47	1 of 1	F	Y	N	high	high	Y	ND	F	undiagnostic	bright	localised	weak	Y	N
GS48	1 of 1	F	N	N	absent	slight	Y	ND	F	absent	n/a	absent	n/a	N	N

Artefact ID	Ground surface #	Shape of ground surface	Low magnification stereomicroscope					Grinding stone type based on morphology	Completeness	High magnification vertical incident light					
			Iron staining	Pecking/ impact damage	Grain levelling	Grain rounding	Macro striae			Use-polish morphology	Use-polish brightness	Use-polish coverage	Use-polish development	Micro striae	Grain fractures
GS49	1 of 1	CV	N	N	high	high	Y	CU	F	slightly undulating/ undiagnostic	moderate	localised	moderate	Y	N
GS50	1 of 1	F	Y	N	absent	moderate	Y	ND	F	absent	n/a	absent	n/a	Y	N
GS53	1 of 1	CV	N	N	high		Y	CU							
GS56	1 of 2	CC	N	Y				CL							
GS56	2 of 2	CC	N	Y											
GS72	1 of 1	CV/U	N	N	minimal	slight	N	CL		absent	n/a	absent	n/a	Y	N
GS73	1 of 1	CC	N	N	moderate	mod-high	Y	F, CL		reticular	bright	extensive	moderate	Y	Y
GS74	1 of 1	F-CC	N	Y	high	mod-high	Y	CL		abrasive smoothing	dull	localised	weak	N	Y
GS75	1 of 1	CC	N	Y	absent	slight	Y	CL		undiagnostic	dull -mod	localised	weak	N	Y
GS79	1 of 1	F	N	N	minimal (weathered)	slight	Y	F		undulating	moderate	moderate	weak	Y	Y
GS80	1 of 1	F/U	N	N	absent	moderate	Y	CL		abrasive smoothing; undiagnostic	dull	localised	weak	N	Y
GS82	1 of 2	CV	N	N	minimal	slight	N	CL		undiagnostic	dull	localised	weak	N	Y
GS82	2 of 2	CV	N	N	minimal	slight	N			undiagnostic	dull	localised	weak	N	Y
GS86	1 of 1	F/CV	N	N	moderate	moderate	Y	CU		undiagnostic	dull	localised	weak	N	Y
GS87	1 of 1	CV/U	N	N	moderate	moderate	Y	CU		undiagnostic	dull	localised	weak	N	Y
UP GS1	1 of 3	F	Y	N	high	high	Y			reticular	bright	moderate	developed	Y	N
UP GS1	2 of 3	CV	Y	N	high	high	Y	CU	F	reticular	bright	moderate	developed	Y	N
UP GS1	3 of 3	F	Y	N	high	high	Y			reticular	bright	moderate	developed	Y	N
UP GS2	1 of 1	CV	N	N	high	high	Y	CU	F	reticular	bright	extensive	developed	Y	N
UP GS3	1 of 1	CV	N	N	high	high	N	CU, F	F	absent	n/a	absent	n/a	Y	Y
UP GS4	1 of 1	CV	Y	N	high	high	Y	F	F	reticular; striated	bright	extensive	w. developed	Y	N
UP GS5	1 of 1	CV	N	N	moderate	high	Y	F	F	undulating/ reticular	bright	moderate	developed	Y	N
UP GS6	1 of 1	F	N	N	moderate	moderate	Y	F	F	slightly undulating/ undiagnostic	moderate	localised	weak	N	N
UP GS7	1 of 1	CV	Y	N	moderate	high	Y	F	F	undiagnostic	moderate	localised	weak	N	N
UP GS9	1 of 1	CV	N	N	high	high	Y	CU	F	reticular	bright	moderate	developed	Y	N
UP GS10	1 of 1	F	N	N	moderate	high	Y	ND	F	absent	n/a	absent	n/a	N	N
UP GS11	1 of 1	CV	N	N	moderate	high	Y	ND	F	reticular	bright	moderate	developed	Y	Y
UP GS12	1 of 1	F	N	N	moderate	slight	N	ND	F	undiagnostic	moderate	localised	weak	N	N
UP GS14	1 of 1	F	N	N	high	high	N	ND	F	reticular	bright	extensive	developed	Y	Y
UP GS15	1 of 1	F	Y	N	minimal	high	Y	F	C	undulating	bright	extensive	w. developed	Y	N

Artefact ID	Ground surface #	Shape of ground surface	Low magnification stereomicroscope					Grinding stone type based on morphology	Completeness	High magnification vertical incident light					
			Iron staining	Pecking/ impact damage	Grain levelling	Grain rounding	Macro striae			Use-polish morphology	Use-polish brightness	Use-polish coverage	Use-polish development	Micro striae	Grain fractures
UP GS16	1 of 3	F-CC	Y	N	high	high	Y	CU	F	reticular	bright	extensive	w. developed	Y	N
UP GS16	2 of 3	F-CC	Y	N	high	high	Y			reticular	bright	extensive	developed	Y	N
UP GS16	3 of 3	F	Y	N	minimal	slight	Y			undiagnostic	bright	moderate	weak	Y	N
UP GS17	1 of 2	F	Y	N	moderate	mod-high	Y	ND	F	reticular	bright	extensive	w. developed	Y	N
UP GS17	2 of 2	F	Y	N	minimal	moderate	Y			undiagnostic	bright	moderate	moderate	Y	N
UP GS18	1 of 1	F	N	N	minimal	moderate	Y	F	F	undiagnostic	moderate	localised	weak	Y	N
UP GS19	1 of 1	CV	N	N	high	mod-high	Y	F	F	slightly undulating, heavily striated/undiagnostic	moderate	localised	moderate	Y	N
UP GS21	1 of 4	F	Y	N	moderate	high	Y	CU, F	C	reticular	bright	extensive	developed	Y	N
UP GS21	2 of 4	F	Y	N	moderate	high	Y			reticular; striated	bright	extensive	developed	Y	N
UP GS21	3 of 4	F	Y	N	minimal	high	N			reticular	bright	extensive	developed	Y	N
UP GS21	4 of 4	F	Y	N	minimal	high	N			reticular	bright	extensive	developed	Y	N
UP GS22	1 of 2	F	N	N	mod-high	high	Y	F	F	undiagnostic	moderate	localised	weak	N	N
UP GS22	2 of 2	F	N	N	mod-high	high	Y			reticular; striated	bright	extensive	developed	Y	N
UP GS23	1 of 1	F	N	N	moderate	moderate	Y	F	F	undulating	mod- bright	moderate	moderate	Y	N
UP GS24	1 of 1	F	N	N	minimal	slight	Y	F	F	undiagnostic	dull	not present	weak	Y	N
UP GS25	1 of 1	CV	N	N	high	high	Y	F	F	undulating	bright	moderate	moderate	Y	N
UP GS26	1 of 1	CV	Y	N	high	high	Y	CU	F	reticular	bright	extensive	w. developed	Y	N
UP GS27	1 of 1	F	Y	N	high	high	Y	F	F	undiagnostic	bright	localised	weak	N	N
UP GS28	1 of 1	CV	Y	N	mod-high	moderate	Y	F	F	undiagnostic	moderate	localised	weak - mod	N	N
UP GS29	1 of 1	F	N	N	high	moderate	Y	F	F	slightly undulating/ undiagnostic	moderate	localised	moderate	Y	Y
UP GS30	1 of 1	CV-F	N	N	moderate	high	Y	ND	F	undiagnostic	dull-moderate	moderate	weak	Y	N
UP GS31	1 of 1	F	N	N	minimal	slight	Y	F	F	slightly undulating/ undiagnostic	bright	localised	weak	Y	N
UP GS32	1 of 1	F	N	N	high	high	Y	ND	F	undiagnostic	dull	localised	weak	Y	N
UP GS33	1 of 1	F	N	N	moderate	slight-mod	N	F	F	undiagnostic	bright	localised	weak	Y	N
UP GS34	1 of 1	F	N	N	moderate	mod-high	Y	F	F	undiagnostic	dull-moderate	localised	weak	N	N
UP GS35	1 of 1	F	Y	N	minimal	slight-mod	Y	F	F	undiagnostic	dull	localised	weak	N	N
UP GS36	1 of 1	F	N	N	high	moderate	Y	F	F	undiagnostic	dull	localised	weak	N	N
UP GS37	1 of 1	CV	N	N	absent	absent	Y	CU	F	undulating	bright	moderate	moderate	Y	N
UP GS38	1 of 1	F	Y	N	moderate	mod-high	Y	CL	C	undiagnostic	moderate	localised	weak	N	N

Artefact ID	Ground surface #	Shape of ground surface	Low magnification stereomicroscope					Grinding stone type based on morphology	Completeness	High magnification vertical incident light					
			Iron staining	Pecking/ impact damage	Grain levelling	Grain rounding	Macro striae			Use-polish morphology	Use-polish brightness	Use-polish coverage	Use-polish development	Micro striae	Grain fractures
UP GS39	1 of 5	F	N	N	high	absent	Y			striated	bright	extensive	w. developed	Y	N
UP GS39	2 of 5	F	N	N	high	absent	Y			undulating; striated	bright	extensive	developed	N	N
UP GS39	3 of 5	F	N	N	high	absent	Y	F	F	undulating; striated	bright	extensive	developed	N	N
UP GS39	4 of 5	F	N	N	high	absent	Y			striated	bright	extensive	developed	Y	N
UP GS39	5 of 5	F	N	N	high	absent	Y			striated	bright	extensive	developed	Y	N
L49	1 of 1	CV	N	N	high	high	Y	CU		reticular	bright	extensive	developed	Y	N
L52	1 of 2	CV	Y	N	high	high	Y	CU		reticular	bright	extensive	developed	Y	Y
L52	2 of 2	F	Y	N	high	high	Y			reticular	bright	moderate	moderate	Y	Y
L813	1 of 1	F	Y	N	moderate	moderate	Y	F		undulating	bright	extensive	developed	Y	N
L868	1 of 1	CV	Y	N	high	high	Y	ND		undiagnostic	dull-mod	localised	weak - mod	Y	N
L894	1 of 1	F	Y	N	high	high	Y	ND		undulating	bright	moderate	moderate	Y	N
L1349	1 of 1	F	Y	N	minimal	moderate	Y	ND		reticular	bright	moderate	moderate	Y	N
R2	1 of 1	F	Y*	N	high	mod-high	N	ND		reticular	bright	extensive	developed	Y	N
R5	1 of 3	CV	Y	N	high	high	Y			reticular	bright	extensive	developed	Y	N
R5	2 of 3	CV	Y	N	high	high	Y	CL		reticular	bright	localised	weak	Y	N
R5	3 of 3	F	Y	N	minimal	high	Y			undiagnostic	moderate	localised	weak	N	N
R66	1 of 3	CV	Y	N	moderate	moderate	N			reticular	bright	extensive	developed	N	N
R66	2 of 3	F	Y	N	moderate	moderate	N	CL		reticular	bright	extensive	developed	N	Y
R66	3 of 3	F	Y	N	moderate	moderate	N			reticular	bright	moderate	moderate	N	N
R68	1 of 2	CC	Y	N	minimal	slight	N	ND		absent	n/a	absent	n/a	N	Y
R68	2 of 2	F	Y	N	high	high	Y			undulating	moderate	moderate	moderate	Y	Y
R69	1 of 1	F	Y	N	high	high	Y	F		undulating	moderate	moderate	moderate	Y	N
R299	1 of 1	F/U	N	N	moderate	moderate	Y	CL		abrasive smoothing w reticular zones	dull-mod	moderate	weak - mod	Y	Y
R305	1 of 1	F/U	N	N	moderate	moderate	N	CL		undiagnostic	dull	localised	weak	Y	Y

SECTION 5: MICROSCOPICALLY VISIBLE RESIDUES ON THE MADJEDBEBE GRINDING STONES & STAINING OF EXTRACTED RESIDUES

5.1 Introduction

Residues are the remnants of material that have been transferred and attached to the artefact surface as a result of cultural and non-cultural processes. Residues from the surfaces of grinding stones are typically identified following extraction (usually with variable pipettes and distilled water or other solvents) and examination under high magnification using a transmitted light microscope. Biological staining agents may be applied to extracted materials to aid identification of unknown particles to confirm their origin (e.g., plant, animal and/or inorganic, see below).

Residue extractions were taken from the used and unused surfaces of each of the Madjedbebe grinding stones using distilled water and/or a tri-solvent mixture of acetonitrile, ethanol and distilled water and examined under an Olympus BX-51 metallographic microscope with objective lenses of x50, x100, x200 and x500 and polarizing filters. Identified particles are listed in Table S4.

5.2 Staining of extracted residues

A common method for identifying residues in an extracted solution is with biological stains, a process that involves the application of various solutions to the extracted material and observing any subsequent related changes in appearance, typically under microscopic conditions. The applied solution, or "stain", will react with a certain component of the residue, turning the designated material a distinctive colour while leaving other constituent materials unaffected.

We selectively used seven different stains to confirm the identity of organic residues documented microscopically in water extractions (viewed under the transmitted light microscope) that had been sampled from the ground (and sometimes unground) surfaces of each grinding stone. Our selection of stains included those that work to indicate the presence of cellulose, lignin, damaged and undamaged starch, protein, collagen and keratin (e.g., hair and feathers), each of which are described alphabetically below. Stains were chosen on the basis of availability, cost and the procedure of application.

5.2.1 Congo Red

Congo Red ($C_{32}H_{22}N_6O_6S_2$Not measured$_2$) is a water-soluble dye that may be used as a general contrast stain for cellulose, amyloid fibrils, and damaged or gelatinised starches (Conn & Lillie 1969; Lamb & Loy 2005: 1439). The stain causes starch and cellulose to stain red while amyloid fibrils will stain green. The latter material is typically only stained in alkaline and acid buffer conditions (i.e., pH 2-4) where the stain is able to bind to carbohydrates, specifically amyloid (Chou *et al.* 2001: 218; Lamb & Loy 2005: 1439; Ramesh & Tharanathan 1999: 347). At a neutral pH, cellulose fibres and damaged or gelatinised starch may be stained in isolation. Both cellulose and starch are composed of the same monosaccharide molecule (glucose), however, differences between the bonds linking the glucose units of both cellulose and starch account for their separate structures and properties (Lamb & Loy 2005: 1434). Undamaged or unaltered starch grains will not become stained with Congo Red as they are hydrophobic and, therefore, will not take up the stain. Alternatively, any alteration of the compact and regular arrangement of the starch layers following heating (e.g., cooking) or mechanical damage (e.g., grinding and pounding) will cause the stain to penetrate into the grain and stain the amylose content within the damaged/altered grains, turning them red. The Congo Red solution was tested prior to application on archaeological artefacts by applying a small amount of stain (up to 5 µL) on heated corn starch in which a positive colour change (red) was identified.

5.2.2 Iodine Potassium Iodide

Iodine Potassium Iodide (IKI) was used to stain intact undamaged starch granules because it is known to bind to the amylose polymers (made up of glucose units) within the starch (Banks & Greenwood 1975: 67; Yeung 1998: 132). The stain provides an immediate colour change causing undamaged starch grains to turn yellow (Plate 5.1b). In time, the starch will turn a permanent dark-blue/black colour (Banks & Greenwood 1975: 67; Evert 2006: 53). The IKI solution was tested prior to application on archaeological artefacts by placing ~5 µL of stain onto a prepared slide containing potato starch in which a positive colour change was identified. The IKI stain also displayed a positive colour change with cellulose material mounted on other experimental slides (instantly staining purple).

5.2.3 Methylene Blue

Methylene Blue ($C_{16}H_{18}N_3SCI$) is a water-soluble dye that may be used to highlight non-lignified cell walls such as cellulose fibres within plant material (Cutler *et al.* 2008: 180; Wilson 1907:

647). The stain binds to the acidic pectins on the cellulose cell wall that are stained various shades of blue (Plate 5.1c) (Lillie 1976: 425; Stadelmann & Kinzel 1972). The colour and intensity of the highlighted cellulose fibres is related to the purity of the material: the darker the blue, the purer the cellulose. Methylene Blue solution was tested prior to application on archaeological artefacts by placing ~5 µL of stain onto a prepared slide containing tissue paper in which a positive colour change was identified. A colour change was not identified on prepared collagen slides.

5.2.4 Orange G

Orange G ($C_{16}H_{10}N_2$Not measured$_2O_7S_2$) is an acidophilic dye used to stain protein and highlight various animal fibres including collagen and keratin, which typically stain orange (Plate 5.1d). The associated change in colour occurs as the stain binds with proteins within the target materials. When used in conjunction with other stains, such as acid fuchsin and malachite green, pollen granules may be stained red (Alexander 1969; Lillie 1976: 121). The Orange G solution was tested prior to application on archaeological artefacts by placing up to 5 µL of stain onto a prepared slide containing a thin section of turkey meat in which a positive colour change was identified. No colour change was observed on slides containing plant materials.

5.2.5 Phloroglucinol

Phloroglucinol ($C_6H_6O_3$) is a water-soluble dye used as a general contrast stain for lignin. The stain reacts with structures within the xylem and sclerenchyma of plant cells to turn the substance red (Plate 5.1e) (Cutler *et al.* 2008: 180; Jensen 1962). However, experimental staining of plant cells using Phloroglucinol of an altered pH, caused lignified tissues to turn a yellow-brown colour. Subsequent analyses of archaeological residues were confirmed for lignin if the latter colour change was observed.

5.2.6 Rhodamine B

Rhodamine B ($C_{28}H_{31}CIN_2O_3$) is a basic dye used to highlight the presence of animal fibres such as hair, feathers and collagen, binding with proteins to allow the target material to turn a pink/purple (Plate 5.1f) (Liisberg 1968; Wessley *et al.* 1981). In general, Rhodamine dyes are water-soluble and are most commonly used in applications of fluorescence microscopy, flow cytometry, fluorescence correlation spectroscopy and ELISA. The Rhodamine B solution was tested prior to application on archaeological artefacts by placing ~5 µL of stain onto a prepared

slide containing highly degraded hair removed from an ancient, Native American leather artefact in which a positive colour change was identified.

5.2.7 Safranin

Safranin ($C_{20}H_{19}ClN_4$) is a staining solution used as a contrast stain to highlight chromosomes, nuclei, lignin, and cell walls. A positive colour change will occur in the presence of the latter two features as the stain reacts with the xylem and sclerenchyma, causing lignified cell walls to turn pink while lignified fibres turn red (Plate 5.1g) (Srebotnik & Messner 1994). The Safranin staining solution was tested prior to application on archaeological residues by placing up to 5 μL of stain onto a prepared slide containing plant cells from a plant stem in which a positive colour change was identified. A colour change was not identified on prepared collagen slides. Because the Safranin stain will highlight other materials such as pollen grains, mitochondria and various animal cells, Phlorogluconol was the preferred stain used for the identification of lignin. The Safranin stain was used only when Phlorogluconol was not available.

Table S4: Residues documented adhering to the tool surface and in pipette extractions. Grey shading indicates artefacts with no plant residues on 19 ground surfaces (of 16 artefacts). Orange shading indicates animal residues on 28 ground surfaces (of 21 artefacts).

Artefact ID	Ground surface #	*In situ* residues (adhering to stone surface), documented with reflected light microscopes	Extracted residues (removed in pipette water extractions), documented with transmitted light microscopes			
			Plant residues	Animal residues	Other organics	Inorganic residues
GS1	1 of 1	red pigment	cellulose, lignin, starch, phytoliths, raphides	-	rootlets, charcoal	red pigment, yellow pigment, sediment
GS2	1 of 2	plant exudate	cellulose, resin	-	-	red pigment, sediment
GS2	2 of 2	plant exudate	cellulose		-	sediment only
GS3	1 of 2	red pigment, amorph. organic material	cellulose, lignin, starch, phytoliths	collagen	-	red pigment, sediment
GS3	2 of 2	none documented	cellulose, lignin, starch, phytoliths, plant sheath	-	-	red pigment, sediment
GS4	1 of 1	red pigment	cellulose, lignin, phytoliths, resin, sieve cell, perforation plate	-	rootlets	red pigment, sediment
GS5	1 of 1	red pigment	cellulose	-	-	sediment only
GS6	1 of 1	red pigment	cellulose	-	-	red pigment, sediment
GS7	1 of 1	red pigment	cellulose, trichome	collagen	-	red pigment, sediment
GS8	1 of 2	red pigment	cellulose	-	-	red pigment, sediment
GS8	2 of 2	none documented	cellulose, resin	-	-	red pigment, sediment
GS9	1 of 1	red pigment	cellulose	collagen, hair	-	sediment only
GS10	1 of 2	hyphae	cellulose	-	hyphae	sediment only
GS10	2 of 2	none documented	cellulose	-	hyphae	sediment only
GS13	1 of 2	rootlets, termite contamination	cellulose	-	hyphae, charcoal	sediment only
GS13	2 of 2	none documented	-	-	-	sediment only
GS14	1 of 3	red pigment	cellulose, lignin	collagen	-	red pigment, sediment
GS14	2 of 3	red pigment	cellulose	-	-	red pigment, sediment
GS14	3 of 3	red pigment	cellulose	-	-	red pigment, sediment
GS15	1 of 1	red pigment	-	-	-	red pigment, sediment
GS16	1 of 2	none documented	cellulose, lignin	-	-	sediment only
GS16	2 of 2	none documented	cellulose, lignin	-	charcoal	sediment only
GS18	1 of 2	none documented	cellulose, lignin	-	-	red pigment, sediment
GS18	2 of 2	none documented	cellulose, lignin	-	-	red pigment, sediment
GS19	1 of 1	red pigment	cellulose	-	-	red pigment, sediment
GS20	1 of 1	cellulose	cellulose	-	-	sediment only
GS21	1 of 1	red pigment	cellulose	-	-	red pigment, sediment
GS22	1 of 1	cellulose	cellulose	-	bacterial spores	sediment only
GS23	1 of 2	none documented	cellulose, lignin	-	-	red pigment, sediment
GS23	2 of 2	none documented	cellulose, lignin	-	-	red pigment, sediment
GS24	1 of 2	red pigment	lignin	-	-	sediment only

Artefact ID	Ground surface #	*In situ* residues (adhering to stone surface), documented with reflected light microscopes	Extracted residues (removed in pipette water extractions), documented with transmitted light microscopes			
			Plant residues	Animal residues	Other organics	Inorganic residues
GS24	2 of 2	none documented	lignin	-	-	red pigment, sediment
GS26	1 of 1	none documented	cellulose, lignin	-	-	sediment only
GS27	1 of 1	red pigment	cellulose, lignin	-	-	red pigment, sediment
GS28	1 of 1	red pigment	cellulose, lignin	collagen	hyphae	sediment only
GS29	1 of 1	red pigment	cellulose, lignin, starch, phytoliths	-	hyphae	sediment only
GS30	1 of 1	red pigment	cellulose, lignin	-	-	red pigment, sediment
GS31	1 of 1	red pigment	cellulose, lignin	-	-	red pigment, sediment
GS32	1 of 2	red pigment	cellulose, lignin, starch, resin	-	-	red pigment, sediment, purple synthetic fibre
GS32	2 of 2	red pigment	cellulose, lignin, phytoliths	-	-	red pigment, sediment
GS33	1 of 1	red pigment	-	-	-	sediment only
GS35	1 of 2	red pigment	cellulose, lignin	collagen	-	red pigment, sediment
GS35	2 of 2	red pigment	cellulose, lignin	collagen, fat*, blood?	-	sediment only
GS36	1 of 2	none documented	cellulose	-	-	sediment only
GS36	2 of 2	red pigment	cellulose	-	charcoal	sediment only
GS37	1 of 1	red pigment	cellulose, lignin	collagen	-	sediment only
GS38	1 of 2	none documented	cellulose, phytoliths	-	lichen spores	sediment only
GS38	2 of 2	none documented	cellulose	-	-	sediment only
GS39	1 of 1	charcoal, cellulose, termite contamination	cellulose, lignin, starch	-	hyphae, charcoal	sediment only
GS40	1 of 1	red pigment	cellulose	-	-	red pigment, sediment
GS41	1 of 1	cellulose	cellulose	-	-	red pigment, sediment
GS43	1 of 1	cellulose	cellulose	-	hyphae, charcoal	sediment only
GS44	1 of 1	none documented	cellulose, lignin	collagen, feather	-	sediment only
GS45	1 of 1	red pigment	cellulose, lignin	-	-	red pigment, sediment
GS46	1 of 2	red pigment	-	-	-	red pigment, sediment, synthetic fibre
GS46	2 of 2	red pigment	-	-	-	red pigment, sediment
GS47	1 of 1	red pigment	starch	-	-	red pigment, sediment
GS48	1 of 1	red pigment	cellulose	-	-	red pigment, sediment
GS49	1 of 1	red pigment	cellulose	-	-	sediment only
GS50	1 of 1	possible	cellulose	-	-	red pigment, sediment
GS53	1 of 1	sediment only	-	-	-	
GS56	1 of 2	sediment only	-	-	-	
GS56	2 of 2	sediment only	-	-	-	
GS72	1 of 1	sediment only	-	-	-	
GS73	1 of 1	red pigment	cellulose, starch	-	-	sediment only

Artefact ID	Ground surface #	*In situ* residues (adhering to stone surface), documented with reflected light microscopes	Extracted residues (removed in pipette water extractions), documented with transmitted light microscopes			
			Plant residues	Animal residues	Other organics	Inorganic residues
GS74	1 of 1	crushed rock	starch	-	-	
GS75	1 of 1	sediment only	-	-	-	
GS79	1 of 1	sediment only	starch	-	-	
GS80	1 of 1	rootlets, termite contamination	-	-	-	
GS82	1 of 2	sediment only	-	-	-	
GS82	2 of 2	sediment only	-	-	-	
GS86	1 of 1	cellulose	-	-	-	
GS87	1 of 1	sediment only	-	-	-	
UPGS1	1 of 3	none documented	cellulose, lignin, phytoliths	collagen	-	sediment only
UPGS1	2 of 3	none documented	cellulose, lignin	collagen	-	sediment only
UPGS1	3 of 3	none documented	cellulose, lignin	collagen	-	sediment only
UPGS2	1 of 1	red pigment	cellulose, starch, phytoliths, raphides, sieve cells	-	-	red pigment, sediment
UPGS3	1 of 1	red pigment, possible bone	cellulose, lignin	collagen	-	red pigment, sediment
UPGS4	1 of 1	red pigment	cellulose, starch, resin	-	-	red pigment, yellow pigment, sediment
UPGS5	1 of 1	none documented	cellulose	-	hyphae	red pigment, sediment
UPGS6	1 of 1	red pigment	cellulose	-	-	red pigment, sediment
UPGS7	1 of 1	red pigment	-	-	-	red pigment, sediment, calcite mineral crust
UPGS9	1 of 1	red pigment	cellulose	collagen	charcoal	red pigment, sediment
UPGS10	1 of 1	none documented	cellulose	-	-	sediment only
UPGS11	1 of 1	red pigment	cellulose	-	-	sediment only
UPGS12	1 of 1	red pigment	cellulose, lignin, starch	-	hyphae	red pigment, sediment
UPGS14	1 of 1	red pigment	cellulose, starch	-	-	red pigment, sediment
UPGS15	1 of 1	charcoal/termite contamination	cellulose	-	-	red pigment, sediment
UPGS16	1 of 3	charcoal, termite contamination	cellulose, lignin	-	hyphae, charcoal	red pigment, sediment
UPGS16	2 of 3	none documented	cellulose, starch	-	-	red pigment, sediment
UPGS16	3 of 3	red and yellow pigment	cellulose, lignin	-	-	red pigment, yellow pigment, sediment
UPGS17	1 of 2	bone?	cellulose	collagen	-	sediment only
UPGS17	2 of 2	red pigment	cellulose, resin?	collagen, feather, blood?	-	red pigment, sediment
UPGS18	1 of 1	red pigment	cellulose, resin?	-	hyphae	red pigment, sediment
UPGS19	1 of 1	red pigment	-	-	-	sediment only
UPGS21	1 of 4	bone, vivianite	cellulose, lignin, phytoliths	bone, vivianite?	-	red pigment, sediment
UPGS21	2 of 4	none documented	cellulose	collagen, bone	-	sediment only
UPGS21	3 of 4	possible bone	cellulose	bone, vivianite?	-	red pigment, sediment

Artefact ID	Ground surface #	*In situ* residues (adhering to stone surface), documented with reflected light microscopes	Extracted residues (removed in pipette water extractions), documented with transmitted light microscopes			
			Plant residues	Animal residues	Other organics	Inorganic residues
UPGS21	4 of 4	none documented	cellulose	-	rootlet	red pigment, sediment
UPGS22	1 of 2	none documented	cellulose	-	-	sediment only
UPGS22	2 of 2	red and yellow pigment	cellulose	-	-	red pigment, yellow pigment, sediment
UPGS23	1 of 1	red pigment, hyphae	-	-	-	red pigment, sediment
UPGS24	1 of 1	red pigment, termite contamination	cellulose	-	charcoal	red pigment, sediment, white mineral
UPGS25	1 of 1	red pigment	cellulose, lignin	-	-	red pigment, sediment
UPGS26	1 of 1	red pigment	cellulose, lignin, starch, phytoliths	collagen	hyphae	red pigment, sediment
UPGS27	1 of 1	charcoal	cellulose	-	charcoal	metal, sediment
UPGS28	1 of 1	red pigment	cellulose, lignin, sieve cell	-	-	sediment only
UPGS29	1 of 1	sediment only	-	-	-	sediment only
UPGS30	1 of 1	waxy coating	cellulose	-	-	sediment only
UPGS31	1 of 1	red pigment	cellulose	-	charcoal	red pigment, sediment
UPGS32	1 of 1	red pigment	cellulose	-	-	red pigment, sediment
UPGS33	1 of 1	sediment only	cellulose, lignin, plant cells	-	-	sediment only
UPGS34	1 of 1	red pigment	cellulose	collagen	-	red pigment, sediment
UPGS35	1 of 1	red pigment, metal	cellulose	-	-	red pigment, metal, sediment
UPGS36	1 of 1	red pigment, metal	-	-	-	red pigment, sediment
UPGS37	1 of 1	sediment only	cellulose	-	hyphae	sediment only
UPGS38	1 of 1	sediment, rootlets, metal, red pigment	cellulose, phytoliths	-	rootlets	red pigment, metal, sediment
UPGS39	1 of 5	red pigment	cellulose, pollen?	collagen, bone	bacterial spores	red pigment, sediment
UPGS39	2 of 5	red pigment	cellulose	collagen	-	red pigment, sediment
UPGS39	3 of 5	red pigment	cellulose	-	-	red pigment, sediment
UPGS39	4 of 5	red pigment	cellulose	-	-	red pigment, sediment
UPGS39	5 of 5	red pigment	cellulose	-	-	red pigment, sediment
L49	1 of 1	red and yellow pigment, cellulose	cellulose, lignin, starch, phytoliths	-	-	red pigment, yellow pigment, sediment
L52	1 of 2	red pigment	cellulose, lignin, starch, perforation plate	-	-	red pigment, yellow pigment, sediment
L52	2 of 2	red and yellow pigment	cellulose	-	-	red pigment, sediment
L813	1 of 1	red mineral pigment	cellulose, lignin, starch, phytoliths, raphides, resin	-	hyphae	red pigment, sediment
L868	1 of 1	red mineral pigment	starch, phytoliths	collagen		red pigment, sediment
L894	1 of 1	sediment only	cellulose	-	-	red pigment, sediment
L1349	1 of 1	sediment only	cellulose, lignin, starch	collagen	decaying root; charcoal	sediment only
R2	1 of 1	red pigment	cellulose, starch, phytoliths	collagen		sediment only

Artefact ID	Ground surface #	*In situ* residues (adhering to stone surface), documented with reflected light microscopes	Extracted residues (removed in pipette water extractions), documented with transmitted light microscopes			
			**Plant residues**	**Animal residues**	**Other organics**	**Inorganic residues**
R5	1 of 3	red mineral pigment	cellulose, lignin, phytoliths, resin, perforation plate		charcoal	red pigment, sediment
R5	2 of 3	red mineral pigment	cellulose	-	-	sediment only
R5	3 of 3	sediment only	cellulose, resin	-	charcoal	sediment only
R66	1 of 3	sediment only	cellulose, lignin, starch	hair	hyphae	sediment only
R66	2 of 3	sediment only	cellulose	-	-	sediment only
R66	3 of 3	sediment only	cellulose	-	-	sediment only
R68	1 of 2	red pigment	cellulose, resin	collagen	hyphae	red pigment, yellow pigment, sediment
R68	2 of 2	red pigment	cellulose, starch, phytoliths	-	hyphae	red pigment, sediment
R69	1 of 1	red pigment	cellulose, lignin	-	hyphae	red pigment, sediment
R299	1 of 1	cellulose	-	-	-	-
R305	1 of 1	sediment only	-	-	-	-

SECTION 6: STARCH GRAIN ANALYSIS

6.1 Introduction

Starch grains are insoluble carbohydrates that form within plant cells consisting of an initial growth point (called the hilum) that is surrounded by layers of amylase and amylopectin (forms of carbohydrate) giving them their characteristic granular form, the layers appearing as 'lamellae' under brightfield microscopy (Evert, 2006). Reichert (1913) was first to explore whether plant taxa could be identified on the basis of starch grains and noted that the size and shape of different starches can often be (broadly) correlated with the source plant taxa. Over the past two decades there has been an increasing number of reports attributing starch grains to specific plant taxa based on features such as grain shape, maximum length through hilum, and the presence or absence of some morphological features such as fissures, surface 'folds', or lamellae and/or vacuoles (e.g., Liu et al., 2010; 2014; Louderback et al., 2017; Musaubach et al., 2013; Piperno et al., 2000). One of the most common effective measures for discriminating between starch grains in comparative reference collections descriptions ishas been the measure of maximum length through the hilum, with a population sample of at least 100 for >100 grains, and the minimum and maximum measurements presented as the size range for that species. More recently, Coster and Field (2015, 2018) and Field et al., (2016), developed a technique methodology based on geometric morphometric analyses, to record and evaluate specific starch grain features attributes for to enable a more reliable and accurate taxonomic identification. In this method, eachEach starch grain is photographed and is manually traced via a digitizing tablet, and with the hilum position, presence of absence of lamellae, fissures, vacuoles and grain facetis was noted located via a digitizing tablet using micrographs of individual grains. A range of geometric attributes can then be extracted from the tracedunkown starch grains shape that are then be compared statistically across large populations of grains derived from the reference species to allow for more reliable taxonomic identifications (see Hayes et al., 2021).

5.2 Extraction and analysis of recovered starch grains

Following pipette extractions from the ground surfaces of grinding stones, residue sampling for ancient starch analsyis was undertaken on a selection of 27 grinding stones (Table S5). The grinding stones were either partially or completely immersed in a beaker of distilled water that was placed in an ultrasonic bath for 2 min. The samples were then transferred to individual 50 ml Falcon tubes and centrifuged for 3 min at 3000 RPM. Starch (and phytoliths) were isolated from the residue sample using heavy liquid separation (Sodium polytungstate, Specific Gravity 2.35) by centrifugation for 15 min at 1000 RPM. Following rinsing in water and centrifugation,

samples were slide-mounted in distilled water and complete slide scans were undertaken using a Zeiss Axioskop II brightfield transmitted light microscope under Differential Interference Contrast (DIC). Images were captured using a Zeiss HRc camera, and archived using Zeiss Axiovision software. Starch grain images were subsequently traced with a WACOM Intuos Pen Tablet (CTH-480) using a graphical user interface (GUI) developed in MATLAB by Adelle Coster (MATLAB Release 2014b, The MathWorks, Inc., Natick, MA, USA) and the size of the grains determined. Raw counts of starch and associated size ranges are shown in Table S5.

5.3 Extraction and analysis of recovered starch grains

A reference collection of well-curated starches of known taxonomic origin and documented as being economically important, was compiled from for the relevant geographic region where Madjedbebe is found was constructed. There are many starch yielding plant species from northern Australia that were exploited by Aboriginal people. Some were eaten raw, others lightly roasted while others were processed by pounding or grinding before cooking (e.g., Thomson, 1939; Wightman and Smith, 1991; Russell Smith et al., 1997, 1985; Fox and Garde, 2018). Some starchy species not generally reported as pounded or ground were also considered as it is well documented that plant foods were prepared for the very young and the infirm. A range of ethnographic sources were consulted to compile a list of relevant plant species (e.g., Smith, 1991; Russell Smith et al., 1997; Meehan et al., 1978; Chaloupka and Giuliani, 1984; Fox and Garde, 2018; Jones and Meehan, 1989; Wightman and Smith, 1991; Purdie et al., 2018; see Hayes et al., 2021). The reference material 2021). We then prepared comparative starch reference material of the relevant plant species usingwas sourced from various field collections from Arnhem Land and the Kimberley region of Western Australia (WA). The starch reference included material from the 1972 Ian Crawford collection (from the Kimberley), which was accessioned at the WA Museum and curated by Dr Moya Smith. The East Kimberley collection was curated by Dr Jenny Atchison and accessioned at the University of Wollongong (UOW), The Kimberley field collections compiled by Matt Barrett, India Dilkes-Hall, Richard Cosgrove and Judith Field were accessioned at the University of New South Wales (UNSW)) John Waterhouse Herbarium. The 2015–2018 Arnhem Land reference collection compiled by S. Anna Florin, May Nango, Djaykuk Djandjomerr and Elspeth Hayes was also sampled. The final species list included 21 samples from 18 genera, and have been described previously in Hayes et al. (2021). Identification of unknown starch grains to plant taxa is not presented here and will be published in detail shortly.

Table S5: Starch grains numbers in extractions from selected grinding stones. The results of a taxonomic analysis of grinding stones L49, UPGS2 and GS3 have been published elsewhere[48].

Artefact ID	Phase	No. of grains	Size Range (mm)	Median Size (mm)
UPGS2	7	133	5.94–25.00	15.60
L49	7	204	5.38–43.6	18.76
UPGS4	6	3	15.52–38.69	-
GS3	5	228	4.59–49.81	10.53
UPGS14	4	3	10.09–25.92	19.48
UPGS26	3	no starch	-	-
UPGS28	3	no starch	-	-
UPGS31	3	no starch	-	-
UPGS32	3	14	12.00–40.71	21.94
GS39	2	7	14.74–29.15	17.13
GS40	2	143	6.42–52.71	17.40
GS44	2	18	6.69–29.98	17.70
GS45	2	15	6.65–24.09	18.22
GS46	2	11	13.15–73.19	19.94
GS47	2	1	18.29	-
GS48	2	35	7.34–36.04	16.82
GS50	2	16	10.54–28.49	18.20
GS73	2	41	10.40–29.17	19.98
GS74	2	39	7.49–51.45	18.43
GS75	2	7	19.18–55.33	32.96
GS79	2	18	9.82–37.09	22.03
GS82	2	Frag 1: 8<br>Frag 2: 40	7.87–61.47	18.95
GS86	2	60	6.45–61.90	16.35
R299	2	2	14.39–17.19	-
R305	2	5	17.11–34.66	-
UPGS34	2	no starch	-	-
UPGS35	2	no starch	-	-

SECTION 7: BIOCHEMICAL TESTS & ABSORBANCE SPECTROSCOPY

7.1 Biochemical tests

Biochemical tests include a range of colorimetric tests in which a reagent is added to a solution to determine whether a certain material or group of compounds is present in the solution. A positive reaction (to indicate the presence of a specific constituent material) is indicated by a colour change in the solution. A suite of biochemical tests was included as part of our residue analysis as a pilot investigation to assess their applicability to potentially very old residue mixtures and to assess the presence of organic compounds. Six tests were included in our analysis: (1) the Bradford Assay test for the detection of proteins; (2) Diphenylamine and Phenol-Sulphuric Acid (PSA) tests for the detection of carbohydrates; (3) Copper triethanolamine diphenyl-carbazide (hereafter referred to as the "Falholt" test) for the detection of fatty acids; (4) Iodide-potassium-iodine (IKI) test for the detection of intact starch; and (5) the Hemastix® test, for the detection of ferrous iron (haem and haemoglobin). These tests were selected for application as they allow for the detection of a wide range of organic materials (in addition to inorganic iron-rich mineral crystals), including various plant and animal tissues. Importantly, the selected tests were also able to be modified so that they may be performed as micro-biochemical tests (requiring less residue material) and analysed using a spectrophotometer. However, the selected tests are only specific for a group of compounds (e.g., protein, carbohydrates), and are unable to identify individual compounds (e.g., collagen, myoglobin). These tests are therefore less sensitive than other methods of residue characterisation such as GC-MS, and have only been used here as an initial screening test for the presence of these specific groups of organic compounds.

Five of the biochemical tests (all but the Hemastix test®) required the application of a colour reagent, which is added to a small portion of the extracted residue solution (see below). The intensity of a subsequent colour change can indicate the concentration of a given compound within the solution. Importantly, all six biochemical tests were able to be modified so that they could be performed as micro-biochemical tests (requiring less solution from the extracted residue mixtures) with subsequent colour changes detected using a spectrophotometer, an instrument that can measure the intensity of light (the number of photons) absorbed after it passes through a very small volume of solution (<5 μL).

The biochemical test reagents (described individually below) were added to individual portions (<5 μL) of solution extracted from the ground surfaces of each grinding stone using either distilled water or a solvent mixture of acetonitrile, ethanol and water in equal parts. To assess the possibility of environmental or other contamination, all accompanying sediment samples (made into solution by mixing with distilled water) and many extractions from the unground surfaces were also tested. Following the application of the biochemical reagents to the residue mixtures, the new solution was observed for a subsequent reaction using an Epoch^{TM} Multi-Volume Spectrophotometer System (described in the main text). Positive readings were determined from a set of standard measurements made on low concentrations of blood protein, corn starch, cooking oil and a combination of sucrose and glucose. The readings from these measured standards (Table S6A) were considered the minimum value for the detection of proteins, starch, fatty acids and carbohydrates, respectively. The specific methods of each test are described below; the measured values for each residue sample are listed in Table S6B.

Table S6A: Measurement standards for biochemical tests

TEST NAME	STANDARDS	neg reading	trace reading	pos. reading
Bradford Assay	blood protein	<0.094	0.094−0.121	>0.121
Iodine Potassium Iodide (IKI)	corn starch	<0.068	0.068−0.079	>0.079
Falholt	cooking oil	<0.767	0.767−0.856	>0.856
Diphenylamine	sucrose/glucose	<0.072;	0.072−0.113;	>0.113
Phenol Sulfuric Acid (PSA)	sucrose/glucose	<0.072	0.072−0.094	>0.094
Hemastix® with EDTA	heamaglobin	0	1−2	3−4

7.1.1 *Biochemical test for protein*

7.1.1.1 *Bradford Assay test*

Protein was detected with the Bradford Assay test following the procedures described by Jones *et al.* (1989) and Kruger (1994). Five micro-litres of water-extracted material was added to 25 μL of Bradford Assay reagent (100 mg of Coomassie Blue G250, 50 mL of 95% ethanol and 100 mL of 85% phosphoric acid; made to 1 L with distilled water) and mixed for 20 min at 1,000 RPM at 25°C. Absorbance was then read for 2 μL of this solution at 595 nm.

7.1.2 *Biochemical tests for carbohydrates*

7.1.2.1 *Diphenylamine test*

Carbohydrates were detected using the Diphenylamine test (Kanzaki & Berger 1959). Five micro-litres of water-extracted sample was mixed with 10 μL of Diphenylamine solution (0.05 g

Diphenylamine (MW 169.22), 5 mL Glacial Acetic Acid and 0.125 mL sulphuric acid) and heated for 10 min at 80°C. Following heating, 2 µL of solution was measured for absorbance at 595 nm.

7.1.2.2 *Phenol-Sulphuric Acid test*

The Phenol-Sulphuric Acid (PSA) test is credited as the easiest and most reliable method of carbohydrate detection (Masuko *et al.* 2005: 69). The test is often used to measure the neutral sugars present within oligosaccharides, proteoglycans, glycoproteins and glycolipids, and was selected as an additional method of carbohydrate detection in the analysis of the MJB and Lake Mungo grinding stones. Five micro-litres of the water-extracted residue solution was mixed with a PSA solution (5 µL 4% Phenol and 25 µL Sulphuric acid). The mixture was left for 10 min at room temperature to ensure adequate binding of the PSA solution to any potential carbohydrates. Following resting, 2 µL of solution were read for absorbance at 490 nm.

7.1.3 *Biochemical test for starch*

7.1.3.1 *Iodine potassium iodine (IKI) test*

The presence of starch (intact and gelatinised) was assessed using the IKI biochemical test (McCready & Hassid 1943). This test was selected owing to the high probability that some of these artefacts were used in the processing of plant materials, and such a test will indicate the presence of starch even if they are unable to be visually identified. Five micro-litres of the water-extracted material removed from each of the used surfaces were mixed to a solution of 5 µL potassium iodide (KI) (0.12M) and 5 µL of iodine (I) (0.01 M). Samples with <5 µL of extraction available were added to smaller portions of KI and I, ensuring that the ratio remained at 1:1:1. Two micro-litres of solution were read for absorbance at 595 nm.

7.1.4 *Biochemical test for fatty acids*

7.1.4.1 *Falholt test*

Fatty acid compounds were detected following the application of the Falholt test (Falholt 1973). This test was considered highly useful for the analyses of grinding stones that may have been used to process materials with a high fatty acid content, such as seeds. Because fatty acids are also present in animal tissues and oily excretions of the hands, further characterisation of specific fatty acid compounds is required to determine the residue source. Residue extractions were freeze-dried for 48 hours so that any additional liquid was removed, and then resuspended in

10 μL of acetonitrile and left for at least 24 hours. Five micro-litres of sample were added to 20 μL of copper triethanolamine (Cu-TEA) [0.05 mol-1 Cu (No3)2 and 0.1 mol-1 thiethanolamine pH 8.1] and 5 μL of diphenyl-carbazide (DPC) (500 μL of 4% 1.5 diphenyl-carbazide and 50 μL of triethanolamine). After 15 min, 2 μL of the mixture were read for absorbance at 550 nm.

7.1.5 *Biochemical test for ferrous iron*

Hemastix®. The presence of haemoglobin (and other iron containing materials) was assessed using the presumptive haemoglobin specific chemical reagent test strip (Hb-CRTS): Seimens Hemastix® test strips. Although no colour reagent is used for this test, this is a colorimetric test whereby a Hemastix® test strip will change colour when haem is detected in a solution. Five micro-litres of solution from the water-extracted residue sample was placed on the Hemastix® test pad and left for 1 min to see if a colour change occurred. If no colour change had occurred after 1 min, the sample was deemed negative for haemoglobin. Evaluations of colour change would not be made after 1 min as the pad can auto-oxidise and change colour, creating a false-positive result. Colour change was ranked on a scale of 0–5 as recommended on the Hemastix package: 0 representing no change in colour; 1 for a speckled colour change and 2–5 for a broad colour change ranked on increasing darkness. These correspond to negative, slight trace, trace, small, moderate, and large traces of haemoglobin, respectively. Any sample that displayed a positive reaction (i.e., colour change ranking from 1–5) was then assessed for contamination by testing the corresponding sediment sample - sediments were submerged in distilled water and the suspended solution was assessed for potential residues causing a positive Hemastix® reaction. Because several other materials found within the burial environment are known to react with Hemastix, for example plant material and metal ions present within the soil (see Section 4.3.2.1), testing the soil sample will indicate whether the positive reaction was instigated by other factors. Those samples from the used surface that provided an initial positive reaction were retested following the addition of 1.0 M ethylenediaminetetraacetic acid (EDTA) solution, which increases the specificity of the test and eliminates the reaction of metal ions within the tested sample (Matheson & Veall 2014). This mixture aims to eliminate any environmental or metal (including haematite) residues that cause a positive reaction in the Hemastix®. A sample that tests positive following the addition of the EDTA solution is likely to contain haemoglobin. In this way, we can determine which artefacts are likely to contain blood and haematite residues.

7.2 Absorbance spectroscopy

Absorbance spectroscopy is a method that can be used in residue analysis to measure and record the spectra of absorption within a solution, allowing the chemical groups of the major constituents within a mixture to be recognised. The method involves the collection of spectral data from a sample that is exposed to a wide spectral range (e.g., 200–900 nm) to produce a "fingerprint" spectrum. The patterning of the spectra permits identification of the various residue components. One advantage of this technique is that analysis requires only a small portion of the residue solution (approximately 2 ɥL) and the majority of residue components can be quantified. However, because this method is less specific (identifying groups of compounds only), and less sensitive than other methods of residue characterisation such as GC-MS, it is more suitable as an initial screening test to confirm the presence of organic materials. In our analysis of the grinding stones from Madjedbebe, we generated absorbance spectra for residue samples extracted from both the ground and unground surfaces using mostly distilled water (Table S6B).

7.2.1 Spectral peaks

Nine peaks within the measured spectra range of 200 and 900 nm were of particular interest; these included: (1) the initial reading between 200 and 205 nm in which a peak height in the graph will indicate the amount of organic material (plant and animal) present within the measured sample); (2) 230 ± 5 nm, indicating the presence of pheolates and carboxyl groups to support fatty acid identification; (3) 240 ± 5 nm, indicating the presence of alcohols, including plant sterols; (4) 250 nm, indicating alkaloids and carbon/nitrogen bonding; (5) 260 nm, indicating the presence of nucleic acids; (6) 270 nm, indicating the presence of phenols; (7) 280 nm, indicating the presence of protein derived from plant material; (8) 410 ± 5 nm, indicating the presence of haemoglobin, myoglobin and animal proteins; and (9) 560 nm, indicating the presence of plant components such as chloroforms and keratins (Hayes, 2015). The identification of distinctive "shoulders" or peaks in the resulting spectra indicated a positive reading.

Table S6B: Biochemical test readings and absorbance spectra data. Dark shaded squares indicate positive readings**.**

Artefact ID	Sample location	Bradford Assay *(proteins)*	Diphenylamine *(carbohydrates)*	Falholt *(fatty acids)*	PSA *(carbohydrates)*	IKI *(starch)*	Hemastix® *(haemoglobin)*	Hemastix® + EDTA *(haemoglobin)*	Absorbance spectra peaks
GS1	ground surface 1 of 1	0.109	0.14	0.504	0.105	0.156	0	not measured	200 nm: generic organic material
GS1	sediment	0.125	0.112	0.496	na	0.098	not measured	not measured	not measured
GS1	unground surface	not measured	not measured	not measured	not measured	not measured	not measured	not measured	not measured
GS2	ground surface 1 of 2	0.122	0.121	0.722	0.145	0.133	0	not measured	200 nm: generic organic material<br>230 ± 5 nm: pheolates & carboxyl groups<br>240 ± 5 nm: alcohols, inc. plant sterols<br>270 nm: phenols<br>280 nm: protein from plants/amino acids
GS2	ground surface 2 of 2	not measured	not measured	0.759	not measured	not measured	not measured	not measured	not measured
GS2	sediment	0.114	0.099	0.624	not measured	0.115	not measured	not measured	not measured
GS2	unground surface	not measured	not measured	not measured	not measured	not measured	not measured	not measured	260 nm: nucleic acids
GS3	ground surface 1 of 2	0.109	0.128	0.9	0.11	0.117	0	not measured	200 nm: generic organic material<br>230 ± 5 nm: pheolates & carboxyl groups<br>250 nm: alkaloids & carbon/nitrogen bonds<br>260 nm: nucleic acids<br>270 nm: phenols
GS3	ground surface 2 of 2	not measured	0.135	0.412	0.097	0.154	0	not measured	200 nm: generic organic material<br>230 ± 5 nm: pheolates & carboxyl groups<br>250 nm: alkaloids & carbon/nitrogen bonds<br>260 nm: nucleic acids<br>270 nm: phenols
GS3	sediment	0.132	0.108	0.516	not measured	0.117	not measured	not measured	not measured
GS3	unground surface	not measured	not measured	not measured	not measured	not measured	not measured	not measured	200 nm: generic organic material
GS4	ground surface 1 of 1	0.117	0.171	0.844	0.134	0.137	3	2	200 nm: generic organic material
GS4	sediment	0.106	0.097	0.361	not measured	0.1	not measured	not measured	not measured
GS4	unground surface	not measured	not measured	not measured	not measured	not measured	0	not measured	200 nm: generic organic material
GS5	ground surface 1 of 1	0.107	0.112	0.793	0.086	0.096	0	not measured	200 nm: generic organic material
GS5	sediment	0.066	0.057	0.532	not measured	0.084	not measured	not measured	not measured
GS5	unground surface	not measured	not measured	not measured	not measured	not measured	not measured	not measured	not measured
GS6	ground surface 1 of 1	0.091	0.097	1.115	0.074	0.074	0	not measured	200 nm: generic organic material
GS6	sediment	0.1	0.085	1.121	not measured	0.094	not measured	not measured	not measured
GS7	ground surface 1 of 1	0.089	0.101	0.863	0.078	0.072	1	0	200 nm: generic organic material
GS7	sediment	0.104	0.088	0.548	not measured	0.077	not measured	not measured	not measured
GS7	unground surface	not measured	not measured	not measured	not measured	not measured	0	not measured	not measured
GS8	ground surface 1 of 2	0.111	0.099	1.007	0.08	0.072	0	not measured	200 nm: generic organic material
GS8	ground surface 2 of 2	not measured	not measured	0.695	not measured	not measured	not measured	not measured	not measured

Artefact ID	Sample location	Bradford Assay *(proteins)*	Diphenylamine *(carbohydrates)*	Falholt *(fatty acids)*	PSA *(carbohydrates)*	IKI *(starch)*	Hemastix® *(haemoglobin)*	Hemastix® + EDTA *(haemoglobin)*	Absorbance spectra peaks
GS8	sediment	0.113	0.075	0.458	not measured	0.079	not measured	not measured	not measured
GS9	ground surface 1 of 1	0.145	0.093	0.989	0.074	0.064	1	0	200 nm: generic organic material
GS9	sediment	0.09	0.076	0.461	not measured	0.071	not measured	not measured	200 nm: generic organic material
GS9	unground surface	not measured	not measured	not measured	not measured	not measured	1	not measured	200 nm: generic organic material
GS10	ground surface 1 of 1	0.101	0.105	0.957	0.091	0.084	3	2	200 nm: generic organic material<br>250 nm: alkaloids & carbon/nitrogen bonds<br>270 nm: phenols
GS10	sediment	0.078	0.065	0.339	not measured	0.06	not measured	not measured	not measured
GS10	unground surface	not measured	not measured	not measured	not measured	not measured	3	0	200 nm: generic organic material
GS13	ground surface 1 of 1	0.143	0.102	0.809	0.08	0.076	3	0	200 nm: generic organic material<br>260 nm: nucleic acids
GS13	sediment	0.1	0.088	0.474	not measured	0.081	not measured	not measured	not measured
GS13	unground surface	not measured	not measured	not measured	not measured	not measured	4	0	200 nm: generic organic material
GS14	ground surface 1 of 3	0.099	0.103	0.796	0.086	0.076	4	2	200 nm: generic organic material<br>250 nm: alkaloids & carbon/nitrogen bonds<br>260 nm: nucleic acids
GS14	ground surface 2 of 3	not measured	not measured	not measured	not measured	not measured	not measured	not measured	not measured
GS14	ground surface 3 of 3	not measured	not measured	not measured	not measured	not measured	not measured	not measured	not measured
GS14	sediment	0.092	0.08	0.361	not measured	0.103	not measured	not measured	not measured
GS14	unground surface	not measured	not measured	not measured	not measured	not measured	3	0	200 nm: generic organic material
GS15	ground surface 1 of 1	0.114	0.102	0.433	0.075	0.076	0	not measured	200 nm: generic organic material<br>260 nm: nucleic acids
GS15	sediment	0.095	0.076	0.394	not measured	0.074	not measured	not measured	not measured
GS15	unground surface	not measured	not measured	not measured	not measured	not measured	not measured	not measured	not measured
GS16	ground surface 1 of 2	0.223	0.141	0.76	0.091	0.089	1	0	200 nm: generic organic material<br>240 ± 5 nm: alcohols, inc. plant sterols
GS16	ground surface 2 of 2	not measured	not measured	0.627	not measured	not measured	not measured	not measured	not measured
GS16	sediment	0.128	0.079	0.37	not measured	0.075	0	not measured	not measured
GS16	unground surface	not measured	not measured	not measured	not measured	not measured	1	0	200 nm: generic organic material<br>250 nm: alkaloids & carbon/nitrogen bonds<br>260 nm: nucleic acids<br>270 nm: phenols
GS18	ground surface 1 of 2	0.122	0.131	0.401	0.104	0.099	0	not measured	not measured
GS18	ground surface 2 of 2	0.108	0.113	0.333	0.098	0.092	0	not measured	not measured
GS18	sediment	not measured	not measured	not measured	not measured	not measured	not measured	not measured	not measured
GS18	unground surface	0.118	0.107	1.144	0.108	0.104	0	not measured	not measured

Artefact ID	Sample location	Bradford Assay *(proteins)*	Diphenylamine *(carbohydrates)*	Falholt *(fatty acids)*	PSA *(carbohydrates)*	IKI *(starch)*	Hemastix® *(haemoglobin)*	Hemastix® + EDTA *(haemoglobin)*	Absorbance spectra peaks
GS19	ground surface 1 of 1	0.117	0.091	0.404	0.717	0.078	3	0	200 nm: generic organic material
GS19	sediment	0.104	0.086	0.54	not measured	0.082	not measured	not measured	not measured
GS19	unground surface	not measured	not measured	not measured	not measured	not measured	4	0	200 nm: generic organic material
GS20	ground surface 1 of 1	0.13	0.145	0.85	0.102	0.105	3	0	200 nm: generic organic material
GS20	sediment	0.097	0.082	0.404	not measured	0.081	not measured	not measured	not measured
GS20	unground surface	not measured	not measured	not measured	not measured	not measured	not measured	not measured	not measured
GS21	ground surface 1 of 1	0.122	0.159	0.993	0.109	0.126	0	not measured	200 nm: generic organic material
GS21	sediment	0.132	0.11	0.387	not measured	0.114	0	not measured	not measured
GS21	unground surface	not measured	not measured	not measured	not measured	not measured	not measured	not measured	200 nm: generic organic material
GS22	ground surface 1 of 1	0.154	0.129	1.113	0.115	0.106	2	0	200 nm: generic organic material
GS22	sediment	0.109	0.099	0.361	not measured	0.122	not measured	not measured	not measured
GS22	unground surface	not measured	not measured	not measured	not measured	not measured	2	0	200 nm: generic organic material
GS23	ground surface 1 of 2	0.129	0.125	1.036	0.108	0.1	3	0	200 nm: generic organic material
GS23	ground surface 2 of 2	not measured	not measured	0.946	not measured	not measured	not measured	not measured	200 nm: generic organic material
GS23	sediment	0.118	0.145	0.424	not measured	0.119	not measured	not measured	not measured
GS23	unground surface	not measured	not measured	not measured	not measured	not measured	3	0	200 nm: generic organic material
GS24	ground surface 1 of 2	not measured	not measured	0.641	not measured	not measured	not measured	not measured	not measured
GS24	ground surface 2 of 2	0.18	0.119	0.347	0.131	0.134	0	not measured	200 nm: generic organic material
GS24	sediment	0.111	0.098	0.393	not measured	0.108	not measured	not measured	not measured
GS24	unground surface	not measured	not measured	not measured	not measured	not measured	not measured	not measured	200 nm: generic organic material
GS26	ground surface 1 of 1	0.113	0.108	0.67	0.095	0.081	0	not measured	200 nm: generic organic material
GS26	sediment	0.065	0.055	0.347	not measured	0.053	not measured	not measured	not measured
GS26	unground surface	not measured	not measured	not measured	not measured	not measured	not measured	not measured	not measured
GS27	ground surface 1 of 1	0.11	0.099	0.688	0.096	0.11	0	not measured	200 nm: generic organic material
GS27	sediment	0.097	0.085	0.404	not measured	0.079	not measured	not measured	not measured
GS27	unground surface	not measured	not measured	not measured	not measured	not measured	not measured	not measured	not measured
GS28	ground surface 1 of 1	0.09	0.096	0.91	0.076	0.073	0	not measured	200 nm: generic organic material
GS28	sediment	0.091	0.078	0.269	not measured	0.074	not measured	not measured	not measured
GS28	unground surface	not measured	not measured	not measured	not measured	not measured	not measured	not measured	not measured
GS29	ground surface 1 of 1	0.127	0.119	0.966	0.071	0.093	0	not measured	200 nm: generic organic material
GS29	sediment	0.091	0.112	0.599	not measured	0.069	not measured	not measured	not measured
GS29	unground surface	not measured	not measured	not measured	not measured	not measured	not measured	not measured	not measured
GS30	ground surface 1 of 1	0.172	0.156	1.218	0.103	0.075	0	not measured	200 nm: generic organic material
GS30	sediment	0.087	0.075	0.442	not measured	0.073	not measured	not measured	not measured
GS30	unground surface	not measured	not measured	not measured	not measured	not measured	not measured	not measured	not measured

Artefact ID	Sample location	Bradford Assay *(proteins)*	Diphenylamine *(carbohydrates)*	Falholt *(fatty acids)*	PSA *(carbohydrates)*	IKI *(starch)*	Hemastix® *(haemoglobin)*	Hemastix® + EDTA *(haemoglobin)*	Absorbance spectra peaks
GS31	ground surface 1 of 1	0.11	0.102	1.17	0.075	0.06	3	0	200 nm: generic organic material
GS31	sediment	0.127	0.066	0.479	not measured	0.061	not measured	not measured	not measured
GS31	unground surface	not measured	not measured	not measured	not measured	not measured	not measured	not measured	not measured
GS32	ground surface 1 of 2	0.115	0.214	1.251	0.152	0.127	0	not measured	200 nm: generic organic material
GS32	ground surface 2 of 2	0.116	0.136	0.848	0.121	0.155	0	not measured	200 nm: generic organic material
GS32	sediment	0.09	not measured	0.479	not measured	0.075	not measured	not measured	not measured
GS32	unground surface	not measured	0.081	not measured	not measured	not measured	not measured	not measured	200 nm: generic organic material
GS33	ground surface 1 of 1	0.115	0.107	1.31	0.091	0.086	1	0	200 nm: generic organic material
GS33	sediment	0.099	0.088	0.494	not measured	0.082	not measured	not measured	not measured
GS33	unground surface	not measured	not measured	not measured	not measured	not measured	0	not measured	200 nm: generic organic material
GS35	ground surface 1 of 2	0.107	0.102	1.17	0.079	0.076	3	0	200 nm: generic organic material
GS35	ground surface 2 of 2	0.115	0.123	1.113	0.09	0.089	3	0	200 nm: generic organic material
GS35	sediment	0.074	0.078	0.426	not measured	0.094	not measured	not measured	not measured
GS35	unground surface	not measured	not measured	not measured	not measured	not measured	1	0	200 nm: generic organic material<br>250 nm: alkaloids & carbon/nitrogen bonds<br>260 nm: nucleic acids<br>270 nm: phenols
GS36	ground surface 1 of 2	0.121	0.093	1.194	0.09	0.079	3	0	200 nm: generic organic material
GS36	ground surface 2 of 2	0.149	0.129	0.658	0.11	0.11	3	0	200 nm: generic organic material
GS36	sediment	0.094	0.089	0.505	not measured	0.071	not measured	not measured	not measured
GS36	unground surface	not measured	not measured	not measured	not measured	not measured	3	0	200 nm: generic organic material
GS37	ground surface 1 of 1	0.067	0.059	0.525	0.055	0.05	0	not measured	200 nm: generic organic material
GS37	sediment	0.1	0.086	0.472	not measured	0.082	not measured	not measured	not measured
GS37	unground surface	not measured	not measured	not measured	not measured	not measured	not measured	not measured	not measured
GS38	ground surface 1 of 2	0.144	0.137	0.529	0.152	0.133	3	0	200 nm: generic organic material<br>240 ± 5 nm: alcohols, inc. plant sterols<br>340 nm: particulate material<br>250 nm: alkaloids & carbon/nitrogen bonds<br>260 nm: nucleic acids<br>270 nm: phenols
GS38	ground surface 2 of 2	0.219	0.135	1.482	0.107	0.107	3	0	200 nm: generic organic material<br>240 ± 5 nm: alcohols, including plant sterols<br>250 nm: alkaloids & carbon/nitrogen bonds<br>260 nm: nucleic acids<br>270 nm: phenols
GS38	sediment	0.19	0.129	0.522	not measured	0.082	not measured	not measured	not measured

Artefact ID	Sample location	Bradford Assay *(proteins)*	Diphenylamine *(carbohydrates)*	Falholt *(fatty acids)*	PSA *(carbohydrates)*	IKI *(starch)*	Hemastix® *(haemoglobin)*	Hemastix® + EDTA *(haemoglobin)*	Absorbance spectra peaks
GS38	unground surface	not measured	not measured	not measured	not measured	not measured	1	0	200 nm: generic organic material
GS39	ground surface 1 of 1	0.126	0.131	1.253	0.114	0.109	3	0	200 nm: generic organic material
GS39	sediment	0.118	0.118	0.349	not measured	0.119	not measured	not measured	not measured
GS39	unground surface	not measured	not measured	not measured	not measured	not measured	3	0	200 nm: generic organic material
GS40	ground surface 1 of 1	0.131	0.113	0.86	0.138	0.133	2	0	200 nm: generic organic material
GS40	sediment	0.245	0.102	0.371	not measured	0.098	not measured	not measured	not measured
GS40	unground surface	not measured	not measured	not measured	not measured	not measured	1	0	200 nm: generic organic material
GS41	ground surface 1 of 1	0.098	0.084	1.032	0.083	0.079	0	not measured	200 nm: generic organic material
GS41	sediment	0.116	0.137	0.384	not measured	0.105	not measured	not measured	not measured
GS41	unground surface	not measured	not measured	not measured	not measured	not measured	not measured	not measured	not measured
GS42	ground surface 1 of 1	0.083	0.083	0.634	0.077	0.072	0	not measured	200 nm: generic organic material
GS42	sediment	0.108	0.104	0.411	not measured	0.114	not measured	not measured	not measured
GS42	unground surface	not measured	not measured	not measured	not measured	not measured	not measured	not measured	200 nm: generic organic material
GS43	ground surface 1 of 1	0.113	0.094	0.596	0.095	0.084	3	0	200 nm: generic organic material
GS43	sediment	0.068	0.107	0.461	not measured	0.055	not measured	not measured	not measured
GS43	unground surface	not measured	not measured	not measured	not measured	not measured	4	0	200 nm: generic organic material
GS44	ground surface 1 of 1	0.105	0.091	0.627	0.081	0.071	3	1	200 nm: generic organic material
GS44	sediment	not measured	0.077	0.485	not measured	0.081	not measured	not measured	not measured
GS44	unground surface	not measured	not measured	not measured	not measured	not measured	3	0	200 nm: generic organic material
GS45	ground surface 1 of 1	0.118	0.099	0.963	0.079	0.073	3	0	200 nm: generic organic material
GS45	sediment	not measured	0.1	0.472	not measured	0.095	not measured	not measured	not measured
GS45	unground surface	not measured	not measured	not measured	not measured	not measured	3	0	200 nm: generic organic material
GS46	ground surface 1 of 2	0.085	0.076	0.815	0.08	0.071	0	not measured	200 nm: generic organic material
GS46	ground surface 2 of 2	not measured	not measured	0.662	not measured	not measured	not measured	not measured	not measured
GS46	sediment	0.103	0.08	0.392	not measured	0.08	not measured	not measured	not measured
GS46	unground surface	not measured	not measured	not measured	not measured	not measured	not measured	not measured	not measured
GS47	ground surface 1 of 1	0.076	0.074	0.839	0.083	0.069	0	not measured	200 nm: generic organic material
GS47	unground surface	0.135	0.122	0.212	not measured	0.101	0	not measured	200 nm: generic organic material
GS48	ground surface 1 of 1	0.111	0.141	1.581	0.076	0.071	0	not measured	200 nm: generic organic material
GS48	sediment	0.104	0.117	0.576	not measured	0.088	not measured	not measured	not measured
GS48	unground surface	not measured	not measured	not measured	not measured	not measured	not measured	not measured	200 nm: generic organic material
GS49	ground surface 1 of 1	0.095	0.093	1.09	0.069	0.065	0	not measured	200 nm: generic organic material
GS49	sediment	not measured	0.105	0.375	not measured	0.073	not measured	not measured	not measured
GS49	unground surface	not measured	not measured	not measured	not measured	not measured	not measured	not measured	200 nm: generic organic material
GS50	ground surface 1 of 1	0.081	0.063	1.087	0.101	0.061	0	not measured	200 nm: generic organic material

Artefact ID	Sample location	**Bradford Assay** *(proteins)*	**Diphenylamine** *(carbohydrates)*	**Falholt** *(fatty acids)*	**PSA** *(carbohydrates)*	**IKI** *(starch)*	**Hemastix®** *(haemoglobin)*	**Hemastix® + EDTA** *(haemoglobin)*	Absorbance spectra peaks
GS50	sediment	0.101	0.099	0.393	not measured	0.061	not measured	not measured	not measured
GS50	unground surface	not measured	not measured	not measured	not measured	not measured	not measured	not measured	not measured
GS53*	ground surface 1 of 1	not measured	not measured	not measured	not measured	not measured	not measured	not measured	not measured
GS53*	sediment	not measured	not measured	not measured	not measured	not measured	not measured	not measured	not measured
GS53*	unground surface	not measured	not measured	not measured	not measured	not measured	not measured	not measured	not measured
GS56*	ground surface 1 of 2	not measured	not measured	not measured	not measured	not measured	not measured	not measured	not measured
GS56*	ground surface 2 of 2	not measured	not measured	not measured	not measured	not measured	not measured	not measured	not measured
GS56*	sediment	not measured	not measured	not measured	not measured	not measured	not measured	not measured	not measured
GS56*	unground surface	not measured	not measured	not measured	not measured	not measured	not measured	not measured	not measured
GS73*	ground surface 1 of 1	not measured	not measured	not measured	not measured	not measured	not measured	not measured	not measured
GS73*	sediment	not measured	not measured	not measured	not measured	not measured	not measured	not measured	not measured
GS73*	unground surface	not measured	not measured	not measured	not measured	not measured	not measured	not measured	not measured
GS74*	ground surface 1 of 1	not measured	not measured	not measured	not measured	not measured	not measured	not measured	not measured
GS74*	sediment	not measured	not measured	not measured	not measured	not measured	not measured	not measured	not measured
GS74*	unground surface	not measured	not measured	not measured	not measured	not measured	not measured	not measured	not measured
GS75*	ground surface 1 of 2	not measured	not measured	not measured	not measured	not measured	not measured	not measured	not measured
GS75*	ground surface 2 of 2	not measured	not measured	not measured	not measured	not measured	not measured	not measured	not measured
GS75*	sediment	not measured	not measured	not measured	not measured	not measured	not measured	not measured	not measured
GS75*	unground surface	not measured	not measured	not measured	not measured	not measured	not measured	not measured	not measured
GS79*	ground surface 1 of 1	not measured	not measured	not measured	not measured	not measured	not measured	not measured	not measured
GS79*	sediment	not measured	not measured	not measured	not measured	not measured	not measured	not measured	not measured
GS79*	unground surface	not measured	not measured	not measured	not measured	not measured	not measured	not measured	not measured
GS80*	ground surface 1 of 1	not measured	not measured	not measured	not measured	not measured	not measured	not measured	not measured
GS80*	sediment	not measured	not measured	not measured	not measured	not measured	not measured	not measured	not measured
GS80*	unground surface	not measured	not measured	not measured	not measured	not measured	not measured	not measured	not measured
GS82*	ground surface 1 of 2	not measured	not measured	not measured	not measured	not measured	not measured	not measured	not measured
GS82*	ground surface 2 of 2	not measured	not measured	not measured	not measured	not measured	not measured	not measured	not measured
GS82*	sediment	not measured	not measured	not measured	not measured	not measured	not measured	not measured	not measured
GS82*	unground surface	not measured	not measured	not measured	not measured	not measured	not measured	not measured	not measured
GS86*	ground surface 1 of 1	not measured	not measured	not measured	not measured	not measured	not measured	not measured	not measured
GS86*	sediment	not measured	not measured	not measured	not measured	not measured	not measured	not measured	not measured
GS86*	unground surface	not measured	not measured	not measured	not measured	not measured	not measured	not measured	not measured
GS87*	ground surface 1 of 1	not measured	not measured	not measured	not measured	not measured	not measured	not measured	not measured
GS87*	sediment	not measured	not measured	not measured	not measured	not measured	not measured	not measured	not measured
GS87*	unground surface	not measured	not measured	not measured	not measured	not measured	not measured	not measured	not measured

Artefact ID	Sample location	Bradford Assay *(proteins)*	Diphenylamine *(carbohydrates)*	Falholt *(fatty acids)*	PSA *(carbohydrates)*	IKI *(starch)*	Hemastix® *(haemoglobin)*	Hemastix® + EDTA *(haemoglobin)*	Absorbance spectra peaks
UPGS1	ground surface 1 of 3	not measured	not measured	0.828	not measured	not measured	not measured	not measured	200 nm: generic organic material
UPGS1	ground surface 2 of 3	0.116	0.133	not measured	0.091	0.085	0	not measured	200 nm: generic organic material
UPGS1	ground surface 3 of 3	not measured	not measured	0.867	not measured	not measured	not measured	not measured	200 nm: generic organic material
UPGS1	sediment	0.093	0.123	0.43	not measured	0.116	0	not measured	not measured
UPGS1	unground surface	not measured	not measured	not measured	not measured	not measured	not measured	not measured	not measured
UPGS2	ground surface 1 of 1	0.087	0.086	1.412	0.089	0.083	0	not measured	200 nm: generic organic material
UPGS2	sediment	not measured	not measured	not measured	not measured	not measured	not measured	not measured	not measured
UPGS2	unground surface	0.111	0.105	0.213	not measured	0.093	not measured	not measured	200 nm: generic organic material
UPGS3	ground surface 1 of 1	0.113	0.102	1.012	0.083	0.079	2	0	200 nm: generic organic material
UPGS3	sediment	not measured	not measured	not measured	not measured	not measured	not measured	not measured	not measured
UPGS3	unground surface	0.114	0.121	0.186	not measured	0.104	0	not measured	200 nm: generic organic material
UPGS4	ground surface 1 of 1	0.137	0.124	0.951	0.092	0.076	0	not measured	200 nm: generic organic material
UPGS4	sediment	0.095	0.088	0.391	not measured	0.077	not measured	not measured	not measured
UPGS4	unground surface	not measured	not measured	not measured	not measured	not measured	not measured	not measured	not measured
UPGS5	ground surface 1 of 1	0.121	0.141	1.081	0.103	0.087	2	0	200 nm: generic organic material
UPGS5	sediment	0.114	0.079	0.397	not measured	0.075	not measured	not measured	not measured
UPGS5	unground surface	not measured	not measured	not measured	not measured	not measured	2	0	200 nm: generic organic material
UPGS6	ground surface 1 of 1	0.143	0.095	1.538	0.078	0.096	3	0	200 nm: generic organic material
UPGS6	sediment	0.106	0.098	0.37	not measured	0.071	not measured	not measured	not measured
UPGS6	unground surface	not measured	not measured	not measured	not measured	not measured	0	not measured	not measured
UPGS7	ground surface 1 of 1	0.093	0.074	1.317	0.081	0.076	0	not measured	200 nm: generic organic material
UPGS7	sediment	0.102	0.08	0.427	not measured	0.083	not measured	not measured	not measured
UPGS7	unground surface	not measured	not measured	not measured	not measured	not measured	not measured	not measured	not measured
UPGS9	ground surface 1 of 1	0.145	0.155	1.053	0.11	0.112	0	not measured	200 nm: generic organic material
UPGS9	sediment	not measured	not measured	not measured	not measured	not measured	not measured	not measured	not measured
UPGS9	unground surface	0.129	0.13	0.231	not measured	0.102	not measured	not measured	200 nm: generic organic material
UPGS10	ground surface 1 of 1	0.085	0.077	0.819	0.077	0.077	0	not measured	200 nm: generic organic material
UPGS10	sediment	0.123	0.107	0.211	not measured	0.096	not measured	not measured	not measured
UPGS10	unground surface	not measured	not measured	not measured	not measured	not measured	not measured	not measured	200 nm: generic organic material
UPGS11	ground surface 1 of 1	0.092	0.084	1.499	0.089	0.085	0	not measured	200 nm: generic organic material
UPGS11	sediment	0.111	0.097	0.266	not measured	0.148	not measured	not measured	not measured
UPGS11	unground surface	not measured	not measured	not measured	not measured	not measured	not measured	not measured	not measured
UPGS12	ground surface 1 of 1	0.125	0.148	0.574	0.121	0.135	0	not measured	200 nm: generic organic material
UPGS12	sediment	0.113	0.104	0.247	not measured	0.121	not measured	not measured	not measured
UPGS12	unground surface	not measured	not measured	not measured	not measured	not measured	not measured	not measured	not measured

Artefact ID	Sample location	Bradford Assay *(proteins)*	Diphenylamine *(carbohydrates)*	Falholt *(fatty acids)*	PSA *(carbohydrates)*	IKI *(starch)*	Hemastix® *(haemoglobin)*	Hemastix® + EDTA *(haemoglobin)*	Absorbance spectra peaks
UPGS14	ground surface 1 of 1	0.136	0.136	0.816	0.11	0.124	3	0	200 nm: generic organic material
UPGS14	sediment	0.108	0.097	0.243	not measured	0.112	not measured	not measured	not measured
UPGS14	unground surface	not measured	not measured	not measured	not measured	not measured	not measured	not measured	200 nm: generic organic material
UPGS15	ground surface 1 of 1	0.125	0.11	0.899	0.102	0.099	0	not measured	200 nm: generic organic material
UPGS15	sediment	not measured	not measured	not measured	not measured	not measured	not measured	not measured	not measured
UPGS15	unground surface	0.215	0.1	0.212	not measured	0.083	not measured	not measured	200 nm: generic organic material
UPGS16	ground surface 1 of 3	0.135	0.127	0.625	0.104	0.109	1	0	200 nm: generic organic material
UPGS16	ground surface 2 of 3	not measured	not measured	0.577	not measured	not measured	0	not measured	200 nm: generic organic material
UPGS16	ground surface 3 of 3	not measured	not measured	0.556	not measured	not measured	not measured	not measured	200 nm: generic organic material
UPGS16	sediment	0.064	0.056	0.23	not measured	0.071	not measured	not measured	not measured
UPGS16	unground surface	not measured	not measured	not measured	not measured	not measured	0	not measured	200 nm: generic organic material
UPGS17	ground surface 1 of 2	0.118	0.116	0.61	0.089	0.084	0	not measured	200 nm: generic organic material
UPGS17	ground surface 2 of 2	not measured	not measured	0.672	not measured	not measured	not measured	not measured	not measured
UPGS17	sediment	not measured	not measured	not measured	not measured	not measured	not measured	not measured	not measured
UPGS17	unground surface	0.11	0.084	0.178	not measured	0.15	not measured	not measured	200 nm: generic organic material
UPGS18	ground surface 1 of 1	0.113	0.096	0.775	0.084	0.085	1	0	200 nm: generic organic material
UPGS18	sediment	0.095	0.085	0.36	not measured	0.113	not measured	not measured	not measured
UPGS18	unground surface	not measured	not measured	not measured	not measured	not measured	0	not measured	200 nm: generic organic material
UPGS19	ground surface 1 of 1	0.11	0.101	0.988	0.093	0.091	0	not measured	200 nm: generic organic material
UPGS19	sediment	0.09	0.078	0.254	not measured	0.071	not measured	not measured	not measured
UPGS19	unground surface	not measured	not measured	not measured	not measured	not measured	not measured	not measured	200 nm: generic organic material
UPGS21	ground surface 1 of 4	0.097	0.095	0.969	0.078	0.071	1	0	200 nm: generic organic material
UPGS21	ground surface 2 of 4	not measured	not measured	0.705	not measured	not measured	not measured	not measured	not measured
UPGS21	ground surface 3 of 4	not measured	not measured	not measured	not measured	not measured	not measured	not measured	not measured
UPGS21	ground surface 4 of 4	not measured	not measured	not measured	not measured	not measured	not measured	not measured	not measured
UPGS21	sediment	0.089	0.071	0.315	not measured	0.075	not measured	not measured	not measured
UPGS21	unground surface	not measured	not measured	not measured	not measured	not measured	0	not measured	200 nm: generic organic material
UPGS22	ground surface 1 of 2	0.11	0.079	0.807	0.091	0.07	0	not measured	200 nm: generic organic material
UPGS22	ground surface 2 of 2	not measured	not measured	1.291	not measured	not measured	not measured	not measured	not measured
UPGS22	sediment	0.087	0.094	0.207	not measured	0.074	not measured	not measured	not measured
UPGS22	unground surface	not measured	not measured	not measured	not measured	not measured	not measured	not measured	200 nm: generic organic material
UPGS23	ground surface 1 of 1	0.12	0.11	0.451	0.105	0.105	0	not measured	200 nm: generic organic material
UPGS23	sediment	0.072	0.062	0.386	not measured	0.081	not measured	not measured	200 nm: generic organic material
UPGS23	unground surface	not measured	not measured	not measured	not measured	not measured	not measured	not measured	200 nm: generic organic material
UPGS24	ground surface 1 of 1	0.097	0.069	1	0.073	0.059	0	not measured	200 nm: generic organic material

Artefact ID	Sample location	Bradford Assay *(proteins)*	Diphenylamine *(carbohydrates)*	Falholt *(fatty acids)*	PSA *(carbohydrates)*	IKI *(starch)*	Hemastix® *(haemoglobin)*	Hemastix® + EDTA *(haemoglobin)*	Absorbance spectra peaks
UPGS24	sediment	0.099	0.108	0.313	not measured	0.08	not measured	not measured	not measured
UPGS24	unground surface	not measured	not measured	not measured	not measured	not measured	not measured	not measured	200 nm: generic organic material
UPGS25	ground surface 1 of 1	0.115	0.099	0.969	0.098	0.095	0	not measured	200 nm: generic organic material
UPGS25	sediment	0.088	0.077	0.512	not measured	0.091	0	not measured	not measured
UPGS25	unground surface	not measured	not measured	not measured	not measured	not measured	not measured	not measured	200 nm: generic organic material
UPGS26	ground surface 1 of 1	0.141	0.107	0.956	0.117	0.083	3	0	200 nm: generic organic material
UPGS26	sediment	0.088	0.073	0.238	not measured	0.08	not measured	not measured	not measured
UPGS26	unground surface	not measured	not measured	not measured	not measured	not measured	1	0	200 nm: generic organic material
UPGS27	ground surface 1 of 1	not measured	not measured	>0.856	not measured	not measured	not measured	not measured	not measured
UPGS27	sediment	0.097	0.103	0.253	not measured	0.07	not measured	not measured	not measured
UPGS27	unground surface	not measured	not measured	not measured	not measured	not measured	not measured	not measured	200 nm: generic organic material
UPGS28	ground surface 1 of 1	0.141	0.107	0.956	0.117	0.083	3	0	200 nm: generic organic material
UPGS28	sediment	0.102	0.096	0.645	not measured	0.091	not measured	not measured	not measured
UPGS28	unground surface	not measured	not measured	not measured	not measured	not measured	1	0	200 nm: generic organic material
UPGS29	ground surface 1 of 1	0.314	0.064	1.545	0.063	0.061	0	not measured	200 nm: generic organic material
UPGS29	sediment	not measured	not measured	not measured	not measured	not measured	not measured	not measured	not measured
UPGS29	unground surface	0.1	0.088	0.144	not measured	0.07	not measured	not measured	200 nm: generic organic material
UPGS30	ground surface 1 of 1	0.106	0.088	1.492	0.087	0.083	0	not measured	200 nm: generic organic material
UPGS30	unground surface	0.128	0.08	0.174	not measured	0.071	not measured	not measured	200 nm: generic organic material
UPGS30	sediment	not measured	not measured	not measured	not measured	not measured	not measured	not measured	not measured
UPGS31	ground surface 1 of 1	0.094	0.079	1.643	0.077	0.071	0	not measured	200 nm: generic organic material
UPGS31	sediment	not measured	not measured	not measured	not measured	not measured	not measured	not measured	not measured
UPGS31	unground surface	0.107	0.095	0.209	not measured	0.095	not measured	not measured	200 nm: generic organic material
UPGS32	ground surface 1 of 1	0.086	0.075	1.104	0.08	0.076	0	not measured	200 nm: generic organic material
UPGS32	sediment	not measured	not measured	not measured	not measured	not measured	not measured	not measured	not measured
UPGS32	unground surface	0.11	0.083	0.147	not measured	0.097	not measured	not measured	200 nm: generic organic material
UPGS33	ground surface 1 of 1	0.101	0.121	1.378	0.078	0.07	0	not measured	200 nm: generic organic material
UPGS33	sediment	not measured	not measured	not measured	not measured	not measured	not measured	not measured	not measured
UPGS33	unground surface	0.093	0.107	0.278	not measured	0.077	not measured	not measured	200 nm: generic organic material
UPGS34	ground surface 1 of 1	0.108	0.084	0.497	0.089	0.083	0	not measured	200 nm: generic organic material
UPGS34	sediment	not measured	not measured	not measured	not measured	not measured	not measured	not measured	not measured
UPGS34	unground surface	0.093	0.079	0.516	not measured	0.075	not measured	not measured	200 nm: generic organic material
UPGS35	ground surface 1 of 1	0.094	0.08	0.307	0.086	0.084	0	not measured	200 nm: generic organic material
UPGS35	sediment	not measured	not measured	not measured	not measured	not measured	not measured	not measured	not measured
UPGS35	unground surface	0.117	0.094	0.809	not measured	0.093	not measured	not measured	200 nm: generic organic material

Artefact ID	Sample location	Bradford Assay *(proteins)*	Diphenylamine *(carbohydrates)*	Falholt *(fatty acids)*	PSA *(carbohydrates)*	IKI *(starch)*	Hemastix® *(haemoglobin)*	Hemastix® + EDTA *(haemoglobin)*	Absorbance spectra peaks
UPGS36	ground surface 1 of 1	0.122	0.105	0.35	0.106	0.098	0	not measured	200 nm: generic organic material
UPGS36	sediment	not measured	not measured	not measured	not measured	not measured	not measured	not measured	not measured
UPGS36	unground surface	0.11	0.083	0.492	not measured	0.083	not measured	not measured	200 nm: generic organic material
UPGS37	ground surface 1 of 1	0.086	0.063	0.795	0.063	0.061	0	not measured	200 nm: generic organic material
UPGS37	sediment	0.087	0.079	0.401	not measured	0.07	0	not measured	not measured
UPGS37	unground surface	not measured	not measured	not measured	not measured	not measured	not measured	not measured	200 nm: generic organic material
UPGS38	ground surface 1 of 1	0.1	0.086	0.526	0.074	0.078	1	0	200 nm: generic organic material
UPGS38	sediment	0.124	0.106	0.193	not measured	0.094	not measured	not measured	not measured
UPGS38	unground surface	not measured	not measured	not measured	not measured	not measured	not measured	not measured	200 nm: generic organic material
UPGS39	ground surface 1 of 5	0.128	0.084	0.386	0.087	0.077	1	0	200 nm: generic organic material
UPGS39	ground surface 2 of 5	not measured	not measured	0.628	not measured	not measured	not measured	not measured	not measured
UPGS39	ground surface 3 of 5	not measured	not measured	0.418	not measured	not measured	not measured	not measured	not measured
UPGS39	ground surface 4 of 5	not measured	not measured	not measured	not measured	not measured	not measured	not measured	not measured
UPGS39	ground surface 5 of 5	not measured	not measured	0.587	not measured	not measured	not measured	not measured	not measured
UPGS39	sediment	0.09	0.078	0.217	not measured	0.084	not measured	0	not measured
UPGS39	unground surface	not measured	not measured	not measured	not measured	not measured	0	not measured	200 nm: generic organic material
L49	ground surface 1 of 1	0.113	0.09	0.391	0.094	0.083	0	not measured	200 nm: generic organic material
L49	sediment	0.309	0.097	0.238	not measured	0.096	not measured	not measured	not measured
L49	unground surface	not measured	not measured	not measured	not measured	not measured	not measured	not measured	200 nm: generic organic material
L52	ground surface 1 of 2	0.121	0.096	0.328	0.098	0.079	0	not measured	200 nm: generic organic material
L52	ground surface 2 of 2	not measured	not measured	not measured	not measured	not measured	not measured	not measured	200 nm: generic organic material
L52	sediment	0.136	0.104	0.201	not measured	0.129	0	not measured	not measured
L52	unground surface	not measured	not measured	not measured	not measured	not measured	not measured	not measured	200 nm: generic organic material
L813	ground surface 1 of 1	0.122	0.129	0.688	0.153	0.112	0	not measured	200 nm: generic organic material
L813	sediment	0.109	0.102	0.181	not measured	0.095	not measured	not measured	not measured
L813	unground surface	not measured	not measured	not measured	not measured	not measured	not measured	not measured	not measured
L868	ground surface 1 of 1	0.068	0.054	0.295	0.056	0.058	0	not measured	200 nm: generic organic material
L868	sediment	0.1	0.083	0.196	not measured	0.07	not measured	not measured	not measured
L868	unground surface	not measured	not measured	not measured	not measured	not measured	not measured	not measured	not measured
L894	ground surface 1 of 1	0.108	0.086	0.594	0.082	0.079	0	not measured	200 nm: generic organic material
L894	sediment	0.073	0.052	0.154	not measured	0.071	not measured	not measured	not measured
L894	unground surface	not measured	not measured	not measured	not measured	not measured	not measured	not measured	not measured
L1349	ground surface 1 of 1	0.149	0.121	0.406	0.136	0.124	0	not measured	200 nm: generic organic material
L1349	sediment	0.098	0.084	0.184	not measured	0.101	not measured	not measured	not measured
L1349	unground surface	not measured	not measured	not measured	not measured	not measured	not measured	not measured	200 nm: generic organic material

Artefact ID	Sample location	Bradford Assay *(proteins)*	Diphenylamine *(carbohydrates)*	Falholt *(fatty acids)*	PSA *(carbohydrates)*	IKI *(starch)*	Hemastix® *(haemoglobin)*	Hemastix® + EDTA *(haemoglobin)*	Absorbance spectra peaks
R2	ground surface 1 of 1	0.139	0.138	0.41	0.11	0.108	0	not measured	200 nm: generic organic material
R2	sediment	0.098	0.076	0.174	not measured	0.077	not measured	not measured	not measured
R2	unground surface	not measured	not measured	not measured	not measured	not measured	not measured	not measured	200 nm: generic organic material
R5	ground surface 1 of 3	0.138	0.143	0.408	0.105	0.107	0	not measured	200 nm: generic organic material
R5	ground surface 2 of 3	not measured	not measured	0.461	not measured	not measured	not measured	not measured	not measured
R5	ground surface 3 of 3	0.113	0.114	0.296	0.437	0.134	0	not measured	200 nm: generic organic material
R5	sediment	0.112	0.075	0.175	not measured	0.075	not measured	not measured	not measured
R5	unground surface	not measured	not measured	not measured	not measured	not measured	not measured	not measured	200 nm: generic organic material
R66	ground surface 1 of 3	0.112	0.087	0.326	0.313	0.083	2	0	200 nm: generic organic material
R66	ground surface 2 of 3	not measured	not measured	not measured	0.638	not measured	not measured	not measured	200 nm: generic organic material
R66	ground surface 3 of 3	not measured	not measured	not measured	not measured	not measured	not measured	not measured	200 nm: generic organic material
R66	sediment	0.085	0.082	0.193	not measured	0.091	not measured	not measured	not measured
R66	unground surface	not measured	not measured	not measured	not measured	3	0	not measured	200 nm: generic organic material
R68	ground surface 1 of 2	0.102	0.076	0.247	0.274	0.08	0	not measured	200 nm: generic organic material
R68	ground surface 2 of 2	not measured	not measured	0.216	0.594	not measured	not measured	not measured	200 nm: generic organic material
R68	sediment	0.079	0.078	0.501	not measured	0.077	not measured	not measured	not measured
R68	unground surface	not measured	not measured	not measured	not measured	not measured	not measured	not measured	200 nm: generic organic material
R69	ground surface 1 of 1	0.105	0.109	0.23	0.466	0.07	0	not measured	200 nm: generic organic material
R69	sediment	0.11	0.087	0.222	not measured	0.093	not measured	not measured	not measured
R69	unground surface	not measured	not measured	not measured	not measured	not measured	not measured	not measured	200 nm: generic organic material

SECTION 8: GAS CHROMATOGRAPHY-MASS SPECTROMETRY

8.1 Introduction

Gas chromatography-mass spectrometry (GC-MS) is a method of residue characterisation that allows non-visible trace elements in residue mixtures to be identified through separation (chromatographic) and identification (mass spectrometric) techniques. The procedure involves separation of molecules within a sample that then are ionised, detected and measured separately to provide information of the biomolecular components within the residue mixture. The presence of certain biomolecules or combinations of biomolecules (the 'chemical fingerprint') within the sample can be related to the compositions known for other organic materials to determine the residue origin may be determined (Evershed, 2008). Organic compounds such as lipids, terpenes, terpenoids, alkanes, proteins and carbohydrates are often the analytes of interest in determining the origin of unknown residues (e.g., Eerkens, 2002; Barnard et al., 2007; Buonasera, 2007; Evershed 2008; Dunne et al., 2012; Villa et al., 2015; Luong et al., 2017, 2018, 2019).

8.2 Biomolecular components of extracted residues from the Madjedbebe grinding stones

Analysis of residue mixtures sampled from the ground surfaces of the grinding stones and fragments from Madjedbebe revealed a range of biomolecular components, including plant and animal-specific compounds (Table S7). Residue mixtures that were abundant in fatty acids and glycerides indicate that the contact material had a high oil content. Monoglycerides are naturally found in seed oils and have been identified on a number of artefacts and often indicate the processing of seeds, nuts and oily fruits (e.g., olives). As there are no oil-producing fruit in the region, the presence of these residues on grinding stones and fragments from northern Australia support the processing of seeds and/or nuts that grow locally in the area. Fats that are derived from animals contain a slightly greater variety of fatty acids and glycerides and thus we were able to distinguish the presence of animal residues from those that may originate from seeds and other oily plants/plant parts (e.g., nuts, leaves and oil-producing fruits). Conversely, carbohydrate-based storage organs such as tubers, rhizomes, corms and many fruits (excepting oil-producing fruits such as olives) contain very few fatty acids and virtually no glycerides. Several samples also contained ascorbic acid (vitamin C) found in tubers, fruit, kernels, seeds and nuts. The presence of these residues on grinding stones and fragments from northern Australia support the processing of seeds and/or nuts which grow locally in the area.

Table S7: List of compounds detected with GC-MS in residue extractions sampled from the Madjedbebe grinding stones and fragments. Compounds with known origins (library matches) are listed in bold text.

Artefact ID	Sample location	Detected compound(s)	Origin/Library match(es) + Reference(s)
GS1	ground surface 1 of 1	acetamide	unknown
		**ethanediol**	**ethylene glycol, present in household cleaning products; paints; cosmetics**
		**2-hydroxy-3-methyl-benzoic acid**	**3-methylsalicylic acid, present in plants (bioactive):** Zulfiqar 1998
		phosphate	unknown
		N-dodecylmethylamine	unknown
		**2-(hydroxymethyl)phenol**	**salicylic alcohol (bioactive)**
		**4-(hydroxymethyl)phenol**	**plant: leaves or twi GSof *Aptenia cordifolia* (bioactive); gastrodigenin:** Della Greca et al. 2007
		3-methyl-12-pyridin-2-yl-8,9,10,12-tetragydro-7H-benzo(b)(4,7)phenanthrolin-11-one	unknown
		**2,6-ditertbutylphenol**	**contamination**
		2(3H)-naphthalenone, 3-hydroxy-4,4a,5,6,7,8-hexahydro-1,4a-dimethyl-7-(1-methylethenyl)-, (3S-(3.alpha.,4a.alpha.,7.alpha.))-	unknown
		**2-phenyl-2-oxphenyl-propane**	**present in plants (bioactive)**
		2-hydroxypentadecane	unknown
		**2,4-diphenyl-4-methyl-2E-pentene**	**present in plants, including seed and/or nuts**
		2-phenyl-2-oxophenyl-propane	unknown
		**monolinoleoylglycerol**	**present in plants:** Bhuiyan et al. 2009; Merlin et al. 2009; Lakshmi prava & RajaLakshmi prava 2011; Malarvizhi & Ramakrishnan 2011; Murugesan & Panneerselvam 2013; Sheela & Uthayakumari 2013
		**11-norcannabinol-9-carboxylic acid**	**present in plants (bioactive):** Aneela et al. 2014
		**hexadecanoic acid**	**degraded fatty acid, present in plants, animals + beeswax; may indicate handling contamination:** Gutiérrez et al. 1999; Malainey et al. 1999; Regert et al. 2001; Croxton et al. 2010; Malarvizhi & Ramakrishnan 2011; Al-Shammari et al. 2012; Lakshmi prava et al. 2012; Abozid et al. 2013; Michalski et al. 2013; Maia & Nunes 2013
		2,2,4-trimethyl-4-(4'-oxyphenol)chromane	unknown
		Bis(4-hydroxyphenyl)methane	unknown
		**2,6-bis(1,1-dimethylethyl)-4-(1-methyl-1-phenylethyl)-phenol**	**pentadecanes (15 Carbons), present in burnt plant material; beeswax; nuts and/or seed:** Kaal et al. 2008, 2009, Lakshmi prava et al. 2012
GS1	sediment	no compounds detected	-
GS1	unground surface	not measured	-
GS2	ground surface 1 of 2	**2-ethylhexanoic acid**	**present in plant; honey:** Fiehn et al. 2000, Jerković & Marijanović 2010, Hammami et al. 2011, Wang et al. 2012
		**oxalic acid**	**present in plants:** Haytowitz & Matthews 1984
		**2-naphthol, 1-(4-dimethylaminophenyl)azo-**	**present in plants:** Gutiérrez et al. 1999
		**1H-indene, 2,3-dihydro-1,1,3-trimethyl-3-phenyl-**	**present in plants**
		2,4-diphenyl-4-methyl-2(E)-pentene	unknown
		2-phenyl-2-oxophenyl-propane	unknown
		**octadecanoic acid, (2-phenyl-1,3-dioxolan-4-yl)methyl ester**	**present in plants:** Hassan et al. 2014
		**hexadecanoic acid**	**degraded fatty acid, present in plants, animals + beeswax; may indicate handling contamination:** Gutiérrez et al. 1999; Malainey et al. 1999; Regert et al. 2001; Croxton et al. 2010; Malarvizhi & Ramakrishnan 2011; Al-Shammari et al. 2012; Lakshmi prava et al. 2012; Abozid et al. 2013; Michalski et al. 2013; Maia & Nunes 2013
		oxirane, 2,2'-((1-methylethylidene)bis(4,1-phenyleneoxymethylene))bis-	unknown
		Bis(4-hydroxyphenyl)methane	unknown

Artefact ID	Sample location	Detected compound(s)	Origin/Library match(es) + Reference(s)
		**2,6-bis(1,1-dimethylethyl)-4-(1-methyl-1-phenylethyl)-phenol**<br><br>cholesta-8,24-dien-3-ol, 4-methyl-, (3.beta.,4.alpha.)-<br>**Rhizoxin**	**pentadecanes (15 Carbons), present in burnt plant material; beeswax; nuts and/or seed:** Kaal et al. 2008, 2009, Lakshmi prava et al. 2012<br>unknown<br>**present in fungus:** Partida-Martinez & Hertweck 2005
GS2	ground surface 2 of 2	methanesulfonylacetic acid<br>2,4-diphenyl-4-methyl-2(E)-pentene<br>**2,4-bis(dimethylbenzyl)-6-t-butylphenol**<br>**phthalic acid, 6-ethyl-3-octyl isobutyl ester**<br>phthalic acid, butyl dodecyl ester<br>**2,6-bis(1,1-dimethylethyl)-4-(1-methyl-1-phenylethyl)-phenol**	unknown<br>unknown<br>**present in plants:** Castrejón et al. 2003<br>**present in plants:** Renjie et al. 2010<br>unknown<br>**pentadecanes (15 Carbons), present in burnt plant material; beeswax; nuts and/or seed:** Kaal et al. 2008, 2009, Lakshmi prava et al. 2012
GS2	sediment	phosphate<br>**azelaic acid**<br><br>**hexadecanoic acid**<br><br><br><br><br>**1-monolinoleoylglycerol**<br><br>**octadecanoic acid**<br><br><br>N-tert-butylacetamide	unknown<br>**degraded fatty acid, present in acne creams, plants + animals:** Nicolet & Liddle 1916; Eerkins 2002; Garelnabi et al. 2010; Al-Shammari et al. 2012<br>**degraded fatty acid, present in plants, animals + beeswax; may indicate handling contamination:** Gutiérrez et al. 1999; Malainey et al. 1999; Regert et al. 2001; Croxton et al. 2010; Malarvizhi & Ramakrishnan 2011; Al-Shammari et al. 2012; Lakshmi prava et al. 2012; Abozid et al. 2013; Michalski et al. 2013; Maia & Nunes 2013<br>**present in plants, e.g., *Calophyllum:*** Bhuiyan et al. 2009, Merlin et al. 2009, Lakshmi & Rajalakshmi 2011, Malarvizhi & Ramakrishnan 2011, Murugesan & Panneerselvam 2013; Sheela & Uthayakumari 2013<br>**fatty acid, present in plants, animals + beeswax; may indicate handling contamination:** Gutiérrez et al. 1999; Malainey et al. 1999; Regert et al. 2001; Croxton et al. 2010; Abirami & Rajendran 2011; Michalski et al. 2013<br>unknown
GS2	unground surface	not measured	-
GS3	ground surface 1 of 2	no compounds detected	-
GS3	ground surface 2 of 2	**2-ethylhexanoic acid**<br><br>2,4-diphenyl-4-methyl-2(E)-pentene<br>2-phenyl-2-oxophenyl-propane<br>**hexadecanoic acid**<br><br><br><br>cholesta-8,24-dien-3-ol, 4-methyl-, (3.beta.,4.alpha.)-<br>**2,4-bis(dimethylbenzyl)-6-t-butylphenol**<br>N-tert-butylacetamide<br>4-(1,1,3,3-tetramethylbutyl)phenol<br>2-phenyl-2-oxophenyl-propane<br>**dodecandioic acid**<br>**(2,8,12,18-tetraethyl-3,7,13,17-tetramethyl-21H, 23H-porphinato(2-)-N21,N22,N23,N24)-, (SP-4-1)-**<br>2,4-diphenyl-4-methyl-2(E)-pentene<br>**cis-10-heptadecenoic acid**<br>**hexadecanoic acid butyl ester**	**present in plants, honey:** Fiehn et al. 2000, Jerković & Marijanović 2010, Hammami et al. 2011, Wang et al. 2012<br>unknown<br>unknown<br>**degraded fatty acid, present in plants, animals + beeswax; may indicate handling contamination:** Gutiérrez et al. 1999; Malainey et al. 1999; Regert et al. 2001; Croxton et al. 2010; Malarvizhi & Ramakrishnan 2011; Al-Shammari et al. 2012; Lakshmi prava et al. 2012; Abozid et al. 2013; Michalski et al. 2013; Maia & Nunes 2013<br>unknown<br>**present in plants:** Castrejón et al. 2003<br>unknown<br>unknown<br>unknown<br>**present in plants:** Chinwe et al. 2014<br>**degraded porphyrin, present in haemaglobin, myoglobin (animal blood)**<br><br>unknown<br>**present in animal fats**<br>**present in plants:** Igwe & Okwu 2013; Sujatha et al. 2014
GS3	sediment	no compounds detected	-

Artefact ID	Sample location	Detected compound(s)	Origin/Library match(es) + Reference(s)
GS3	unground surface	not measured	-
GS4	ground surface 1 of 1	**ethanediol**	**ethylene glycol, present in household cleaning products; paints; cosmetics**
		cyclohexylamine	unknown
		1-cyclohexenol	unknown
		**2-hydroxypropanoic acid**	**lactic acid, present in plants + animals**
		**2-ethylhexanoic acid**	**present in plants, honey:** Fiehn et al. 2000, Jerković & Marijanović 2010, Hammami et al. 2011, Wang et al. 2012
		**benzoic acid**	**present in plants (antibacterial), e.g., *Petalostigma, Ficus:*** Fountain et al. 1995; Cock & Kalt 2012; Kalt & Cock 2014; Jeong et al. 2014; Saravanan et al. 2014
		phosphate	unknown
		**2-(hydroxymethyl)phenol**	**salicylic alcohol (bioactive)**
		2-phenyl-2-oxophenyl-propane	unknown
		**hexadecanoic acid**	**degraded fatty acid, present in plants, animals + beeswax; may indicate handling contamination:** Gutiérrez et al. 1999; Malainey et al. 1999; Regert et al. 2001; Croxton et al. 2010; Malarvizhi & Ramakrishnan 2011; Al-Shammari et al. 2012; Lakshmi prava et al. 2012; Abozid et al. 2013; Michalski et al. 2013; Maia & Nunes 2013
		oxirane, 2,2'-((1-methylethylidene)bis(4,1-phenyleneoxymethylene))bis-	unknown
		Bis(4-hydroxyphenyl)methane	unknown
		**2,6-bis(1,1-dimethylethyl)-4-(1-methyl-1-phenylethyl)-phenol**	**pentadecanes (15 Carbons), present in burnt plant material; beeswax; plant residue inc. nuts and/or seed:** Kaal et al. 2008, 2009, Lakshmi prava et al. 2012
		Bis(4-hydroxyphenyl)methane	unknown
		**octadecanoic acid**	**fatty acid, present in plants, animals + beeswax; may indicate handling contamination:** Gutiérrez et al. 1999; Malainey et al. 1999; Regert et al. 2001; Croxton et al. 2010; Abirami & Rajendran 2011; Michalski et al. 2013
		2,4-imidazolidinedione, 5-(3,4-dihydroxy-phenol)-3-methyl-5-phenyl-	unknown
		1-hydroxy-4-hydroxymethyl-phenol	unknown
		**2,4-bis(dimethylbenzyl)-6-t-butylphenol**	**present in plants:** Castrejón et al. 2003
		**2,6-dihydroxybenzoic acid**	**fungal mycobiont: Takenaka et al. 2011**
		2,4-dihydroxy-3-methylbenzoic acid	unknown
		**2-ethylhexanoic acid**	**present in plants, honey:** Fiehn et al. 2000, Jerković & Marijanović 2010, Hammami et al. 2011, Wang et al. 2012
		**benzoic acid**	**present in plants (antibacterial), e.g., *Petalostigma, Ficus:*** Fountain et al. 1995; Cock & Kalt 2012; Kalt & Cock 2014; Jeong et al. 2014; Saravanan et al. 2014
		2-phenyl-2-oxophenyl-propane	unknown
		oxirane, 2,2'-((1-methylethylidene)bis(4,1-phenyleneoxymethylene))bis-	unknown
GS4	sediment	no compounds detected	-
GS4	unground surface	not measured	-
GS5	ground surface 1 of 1	**2-ethylhexanoic acid**	**present in plants, honey:** Fiehn et al. 2000, Jerković & Marijanović 2010, Hammami et al. 2011, Wang et al. 2012
		**benzoic acid**	**present in plants (antibacterial), e.g., *Petalostigma, Ficus:*** Fountain et al. 1995; Cock & Kalt 2012; Kalt & Cock 2014; Jeong et al. 2014; Saravanan et al. 2014
		2-phenyl-2-oxophenyl-propane	unknown
		**2,4,6-trimethyldecane**	**undecanes (13 Carbons), present in burnt plant material:** Kaal et al. 2008, 2009
		**5-methyl-2-phenylindolizine**	**present in plants, e.g., *Cissus:*** Rosy & Rosakutty 2012
		**dodecanol**	**present in plants:** Soleimani et al. 2009; Faridah et al. 2010; Sharopov et al. 2010
		**5,8-diethyldodecane**	**hexadecanes (16 Carbons), present in burnt plant material:** Kaal et al. 2008
		2-phenyl-2-oxophenyl-propane	unknown

Artefact ID	Sample location	Detected compound(s)	Origin/Library match(es) + Reference(s)
		phenylpropylamine, N-acetyl-3,4-dimethoxy-	unknown
		2-phenyl-2-oxophenyl-propane	unknown
		**hexadecanoic acid**	**degraded fatty acid, present in plants, animals + beeswax; may indicate handling contamination:** Gutiérrez et al. 1999; Malainey et al. 1999; Regert et al. 2001; Croxton et al. 2010; Malarvizhi & Ramakrishnan 2011; Al-Shammari et al. 2012; Lakshmi prava et al. 2012; Abozid et al. 2013; Michalski et al. 2013; Maia & Nunes 2013
		2,2,4-trimethyl-4-(4'-oxyphenol)chromane	unknown
		Bis(4-hydroxyphenyl)methane	unknown
		**2,6-bis(1,1-dimethylethyl)-4-(1-methyl-1-phenylethyl)-phenol**	**pentadecanes (15 Carbons); present in burnt plant material; beeswax; nuts and/or seeds:** Kaal et al. 2008, 2009; Lakshmi prava et al. 2012
		1,1',2,2'-tetrahydro-1,1'-dimethoxy-carotene	unknown
		Bis(4-hydroxyphenyl)methane	unknown
		**octadecanoic acid**	**fatty acid, present in plants, animals + beeswax; may indicate handling contamination:** Gutiérrez et al. 1999; Malainey et al. 1999; Regert et al. 2001; Croxton et al. 2010; Abirami & Rajendran 2011; Michalski et al. 2013
		**2,4-bis(dimethylbenzyl)-6-t-butylphenol**	**present in plants:** Castrejón et al. 2003
		**ethanediol**	**ethylene glycol, present in household cleaning products; paints; cosmetics**
		**phenol**	**present in plants, e.g., *Ficus*:** Saravanan et al. 2014
		**2-hydroxy-ethanoic acid**	**glycolic acid**
		glycerol	unknown
		**2-methoxyphenol**	**guaiacol, wood, fruit e.g., *Ficus* (antimicrobial):** van Bergen & Poole 2002, Ragasa et al. 2014, Saravanan et al 2014
		**2,6,10-trimethyl-tetradecane**	**tridecanes (13 Carbons), present in burnt plant material:** Kaal et al. 2008, 2009
		**2,6-dimethyl-heptadecane**	**nonadecanes (19 Carbons), present in burnt plant material, beeswax:** Kaal et al. 2008, 2009, Lakshmi prava et al. 2012
		octanol	unknown
		2-phenyl-2-oxophenyl-propane	unknown
		2-tert-butyl-4-methyl-6-(a-methylbenzyl)phenol	unknown
		**serverogenin acetate**	**present in plants, e.g., *Trichilia* sp.:** Senthilkumar et al. 2012
		**phthalic acid, butyl nonyl ester**	**contamination**
		**hexadecanoic acid**	**degraded fatty acids, present in plants, animals + beeswax, may indicate handling contamination:** Gutiérrez et al. 1999; Malainey et al. 1999; Regert et al. 2001; Croxton et al. 2010; Malarvizhi & Ramakrishnan 2011; Al-Shammari et al. 2012; Lakshmi prava et al. 2012; Abozid et al. 2013; Michalski et al. 2013; Maia & Nunes 2013
		(4-hydroxyphenyl)(2-hydroxyphenyl)methane	unknown
		Bis(4-hydroxyphenyl)methane	unknown
		acetonitrile	unknown
		**octadecanoic acid**	**fatty acid, present in plants, animals + beeswax; may indicate handling contamination:** Gutiérrez et al. 1999; Malainey et al. 1999; Regert et al. 2001; Croxton et al. 2010; Abirami & Rajendran 2011; Michalski et al. 2013
GS5	sediment	no compounds detected	-
GS5	unground surface	not measured	-
GS6	ground surface 1 of 1	**hexadecanoic acid**	**degraded fatty acid, present in plants, animals + beeswax; may indicate handling contamination:** Gutiérrez et al. 1999; Malainey et al. 1999; Regert et al. 2001; Croxton et al. 2010; Malarvizhi & Ramakrishnan 2011; Al-Shammari et al. 2012; Lakshmi prava et al. 2012; Abozid et al. 2013; Michalski et al. 2013; Maia & Nunes 2013
		**octadecanoic acid**	

Artefact ID	Sample location	Detected compound(s)	Origin/Library match(es) + Reference(s)
			**fatty acid, present in plants, animals + beeswax; may indicate handling contamination:** Gutiérrez et al. 1999; Malainey et al. 1999; Regert et al. 2001; Croxton et al. 2010; Abirami & Rajendran 2011; Michalski et al. 2013
GS6	sediment	no compounds detected	-
GS7	ground surface 1 of 1	**phenol**	**Present in plants, e.g., *Ficus*:** Saravanan et al. 2014
		**2-ethylhexanoic acid**	**present in plants, honey:** Fiehn et al. 2000, Jerković & Marijanović 2010, Hammami et al. 2011, Wang et al. 2012
		**benzoic acid**	**present in plants (antibacterial) e.g., *Petalostigma, Ficus:*** Fountain et al. 1995; Cock & Kalt 2012; Kalt & Cock 2014; Jeong et al. 2014; Saravanan et al. 2014
		2-phenyl-2-oxophenyl-propane	unknown
		**nonanoic acid**	**present in plants:** Knudsen et al. 1993
		2-hydroxymethylphenol	unknown
		**hexadecane**	**hexadecanes (16 Carbons), present in plant *Indigofera*, burnt plant material:** Kaal et al. 2008; Deshpande et al. 2013,
		**17-octadecynoic acid**	**present in plants, e.g., *Indigofera:*** Deshpande et al. 2013; Reddy et al. 2014
			unknown
		2,4-diphenyl-4-methyl-2(E)-pentene	unknown
		2-phenyl-2-oxophenyl-propane	unknown
		**hexadecanoic acid**	**degraded fatty acid, present in plants, animals + beeswax; may indicate handling contamination:** Gutiérrez et al. 1999; Malainey et al. 1999; Regert et al. 2001; Croxton et al. 2010; Malarvizhi & Ramakrishnan 2011; Al-Shammari et al. 2012; Lakshmi prava et al. 2012; Abozid et al. 2013; Michalski et al. 2013; Maia & Nunes 2013
		oxirane, 2,2'-((1-methylethylidene)bis(4,1-phenyleneoxymethylene))bis-	unknown
		(4-hydroxyphenyl)(2-hydroxyphenyl)methane	unknown
		**2,6-bis(1,1-dimethylethyl)-4-(1-methyl-1-phenylethyl)-phenol**	**pentadecanes (15 Carbons); burnt plant material; beeswax; nuts and/or seeds:** Kaal et al. 2008, 2009**;** Lakshmi prava et al. 2012
		**Pregan-20-one, 2-hydroxy-5,6-epoxy-15-methyl-**	**propolis, present in beeswax (bioactive):** Abozid et al. 2013, Shubharani et al. 2014
		5,8,11-eicosatriynoic acid	unknown
		Bis(4-hydroxyphenyl)methane	unknown
		5,8,11-eicosatriynoic acid	unknown
		**2,4-bis(dimethylbenzyl)-6-t-butylphenol**	**present in plants:** Castrejón et al. 2003
		**hydroxylamine, O-decyl-**	**present in plants:** Senthilkumar et al. 2012
		10-methylundecanoic acid, methyl ester	unknown
		2,4-diphenyl-4-methyl-2(E)-pentene	unknown
		2-methylbenzoic acid anhydride	unknown
		2,4-diphenyl-4-methyl-2(E)-pentene	unknown
		**Glycine, N-((3.alpha.,5.beta.,7.alpha.,12.alpha.)-24-oxo-3,7,12-trihydroxy-cholan-24-yl)-, methyl ester**	**amino acid derivative**
		2-tert-butyl-4-methyl-6-(a-methylbenzyl)phenol	unknown
		phthalic acid, butyl undec-2-en-1-yl ester	unknown
		phthalic acid, decyl hex-2-yn-4-yl ester	unknown
		2-(1,1-dimethylethyl)-4-(1-methyl-1-phenylethyl)-phenol	unknown
		2-(1,1-dimethylethyl)-4-(1-methyl-1-phenylethyl)-phenol	unknown
		**ethyl iso-allocholate**	**present in plants (bioactive), e.g., *Ficus*:** Sarada et al. 2011, Saravanan et al. 2014
		**9-(methoxyimino)-11,15-dihydroxy-Prost-13-en-1-oic acid**	**linolenic acid, present in plants, seeds:** Liu et al. 2000; Xue et al. 2008; Minzangi et al. 2011
		**serine, N,O-bis(m-toluoyl)-, methyl ester**	**amino acid derivative, unknown origin**

Artefact ID	Sample location	Detected compound(s)	Origin/Library match(es) + Reference(s)
		2,4-bis(1-methyl-1-phenylethyl)-phenol	unknown
		**hexadecanoic acid**	**degraded fatty acid, present in plants, animals + beeswax; may indicate handling contamination:** Gutiérrez et al. 1999; Malainey et al. 1999; Regert et al. 2001; Croxton et al. 2010; Malarvizhi & Ramakrishnan 2011; Al-Shammari et al. 2012; Lakshmi prava et al. 2012; Abozid et al. 2013; Michalski et al. 2013; Maia & Nunes 2013
		**octadecanoic acid**	**fatty acid, present in plants, animals + beeswax; may indicate handling contamination:** Gutiérrez et al. 1999; Malainey et al. 1999; Regert et al. 2001; Croxton et al. 2010; Abirami & Rajendran 2011; Michalski et al. 2013
GS7	sediment	**hexadecanoic acid**	**degraded fatty acid, present in plants, animals + beeswax; may indicate handling contamination:** Gutiérrez et al. 1999; Malainey et al. 1999; Regert et al. 2001; Croxton et al. 2010; Malarvizhi & Ramakrishnan 2011; Al-Shammari et al. 2012; Lakshmi prava et al. 2012; Abozid et al. 2013; Michalski et al. 2013; Maia & Nunes 2013
		**octadecanoic acid**	**fatty acid, present in plants, animals + beeswax; may indicate handling contamination:** Gutiérrez et al. 1999; Malainey et al. 1999; Regert et al. 2001; Croxton et al. 2010; Abirami & Rajendran 2011; Michalski et al. 2013
GS7	unground surface	not measured	-
GS8	ground surface 1 of 2	**1,1'-biphenyl, 4,4'-dinitro-**	**present in plants (bioactive)**
		2-hydroxy-2-cyclopenten-1-one	unknown
		**phenol**	**present in plants, e.g., *Ficus*:** Saravanan et al. 2014
		**2-hydroxypropanoic acid**	**lactic acid**
		**2-ethylhexanoic acid**	**present in plants, honey:** Fiehn et al. 2000, Jerković & Marijanović 2010, Hammami et al. 2011, Wang et al. 2012
		**benzoic acid**	**present in plants (antibacterial), e.g., *Petalostigma, Ficus:*** Fountain et al. 1995; Cock & Kalt 2012; Kalt & Cock 2014; Jeong et al. 2014; Saravanan et al. 2014
		phosphate	unknown
		**2-hydroxymethyl-phenol**	**salicyl alcohol, present in plants (bioactive):** Mahdi 2010
		2-methylnonadecane	unknown
		**4-hydroxybenzaldehyde**	**present in plants, e.g., *Mimusops, Solanum* (bioactive-medicinal):** Kuo et al. 2008; Ren et al. 2009; Rao et al. 2012
		2,4-diphenyl-4-methyl-2(E)-pentene	unknown
		2-phenyl-2-oxophenyl-propane	unknown
		**serverogenin acetate**	**present in plants, e.g., *Trichilia* sp.:** Senthilkumar et al. 2012
		**11-norcannabinol-9-carboxylic acid**	**present in plants (bioactive):** Aneela et al. 2014
		Bis(4-hydroxyphenyl)methane	unknown
		**2,6-bis(1,1-dimethylethyl)-4-(1-methyl-1-phenylethyl)-phenol**	**pentadecanes (15 Carbons); present in burnt plant material; beeswax; nuts and/or seed:** Kaal et al. 2008, 2009, Lakshmi prava et al. 2012
		Bis(4-hydroxyphenyl)methane	unknown
		**Tyramine**	**alkaloid, present in toxic plants, e.g., *Acacia* sp., *Phalaris:*** Adams & Camp 1966; Camp & Norvell 1966; Clement et al. 1998; Culvenor et al. 2005
		**hexadecanoic acid, 1,1-dimethylethyl ester**	**present in plants:** Prakash et al. 2011
		**octadecanoic acid**	**fatty acid, present in plants, animals + beeswax; may indicate handling contamination:** Gutiérrez et al. 1999; Malainey et al. 1999; Regert et al. 2001; Croxton et al. 2010; Abirami & Rajendran 2011; Michalski et al. 2013
		2-hydroxy-4-hydroxymethyl-phenol	unknown
		**2,4-bis(dimethylbenzyl)-6-t-butylphenol**	**present in plants:** Castrejón et al. 2003
		**hexadecanoic acid**	**degraded fatty acid, present in plants, animals + beeswax; may indicate handling contamination:** Gutiérrez et al. 1999; Malainey et al. 1999; Regert et al. 2001; Croxton

Artefact ID	Sample location	Detected compound(s)	Origin/Library match(es) + Reference(s)
			et al. 2010; Malarvizhi & Ramakrishnan 2011; Al-Shammari et al. 2012; Lakshmi prava et al. 2012; Abozid et al. 2013; Michalski et al. 2013; Maia & Nunes 2013
		**octadecanoic acid**	**fatty acid, present in plants, animals + beeswax; may indicate handling contamination:** Gutiérrez et al. 1999; Malainey et al. 1999; Regert et al. 2001; Croxton et al. 2010; Abirami & Rajendran 2011; Michalski et al. 2013
GS8	ground surface 2 of 2	**1H-indene, 2,3-dihydro-1,1,3-trimethyl-3-phenyl-**	**present in plants**
		2,4-diphenyl-4-methyl-2(E)-pentene	unknown
		**phthalic acid, octyl tridec-2-yn-1-yl ester**	**present in plants:** Senthilkumar et al. 2012
		**2,6,10,15-tetramethyl-heptadecane**	**heneicosanes (21 Carbons); present in burnt plant material, beeswax:** Kaal et al. 2008, Lakshmi prava et al. 2012, Maia & Nunes 2013
		2-(1,1-dimethylethyl)-4-(1-methyl-1-phenylethyl)-phenol	unknown
		**2,6-bis(1,1-dimethylethyl)-4-(1-methyl-1-phenylethyl)-phenol**	**pentadecanes (15 Carbons); present in burnt plant material; beeswax; nuts and/or seeds:** Kaal et al. 2008, 2009, Lakshmi prava et al. 2012
		**hexadecanoic acid, butyl ester**	**present in plants:** Igwe & Okwu.2013, Sujatha et al. 2014
		**heptadecanoic acid, heptadecyl ester**	**present in plants:** Bharathy & Uthayakumari 2013; Tyagi & Sharma 2014
		**2,4-bis(dimethylbenzyl)-6-t-butylphenol**	**present in plants:** Castrejón et al. 2003
		2,4-bis(1-methyl-1-phenylethyl)-phenol	unknown
		1,2-propanediol, 3-(octadecyloxy)- acetate	unknown
GS8	sediment	no compounds detected	-
GS9	ground surface 1 of 1	**2,6,10-trimethyl-tetradecane**	**tridecanes (13 Carbons), present in burnt plant material:** Kaal et al. 2008, 2009
		**1H-indene, 2,3-dihydro-1,1,3-trimethyl-3-phenyl-**	**present in plants**
		4,4'-(1-methylethylidene)bis(2,6-dimethyl)-phenol	unknown
		phthalic acid, butyl tetradecyl ester	unknown
		**2-ethyl-2-methyl-tridecanol**	**present in algae, plants:** Ololade & Olawore 2013, Sathya et al. 2012, Al-Mazroa et al. 2015
		**phenylalanine, 4-amino-N-t-butyloxycarbonyl-, t-butyl ester**	**amino acid derivative**
		**hexadecanoic acid**	**degraded fatty acid, present in plants, animals + beeswax; may indicate handling contamination:** Gutiérrez et al. 1999; Malainey et al. 1999; Regert et al. 2001; Croxton et al. 2010; Malarvizhi & Ramakrishnan 2011; Al-Shammari et al. 2012; Lakshmi prava et al. 2012; Abozid et al. 2013; Michalski et al. 2013; Maia & Nunes 2013
		2-(1,1-dimethylethyl)-4-(1-methyl-1-phenylethyl)-phenol	unknown
		**2-methyl-eicosane**	**present in plants:** Siddiquee et al. 2012; Santhosh Kumar et al. 2014
		1,1',2,2'-tetrahydro-1,1'-dimethoxy-carotene	unknown
		**hexadecanoic acid butyl ester**	**present in plants:** Igwe & Okwu 2013; Sujatha et al. 2014
		**eicosane**	**(20 carbons), present in plants + burnt plant material:** Kaal et al. 2008, Senthilkumar et al. 2012
		octadecanol	unknown
		**2,4-bis(dimethylbenzyl)-6-t-butylphenol**	**present in plants:** Castrejón et al. 2003
		**2,6,10,15-tetramethyl-heptadecane**	**heneicosanes (21 Carbons), present in burnt plant material; beeswax:** Kaal et al. 2008, Lakshmi prava et al. 2012, Maia & Nunes 2013
GS9	sediment	**hexadecanoic acid**	**degraded fatty acid, present in plants, animals + beeswax; may indicate handling contamination:** Gutiérrez et al. 1999; Malainey et al. 1999; Regert et al. 2001; Croxton et al. 2010; Malarvizhi & Ramakrishnan 2011; Al-Shammari et al. 2012; Lakshmi prava et al. 2012; Abozid et al. 2013; Michalski et al. 2013; Maia & Nunes 2013
		**octadecanoic acid**	**fatty acid, present in plants, animals + beeswax; may indicate handling contamination:** Gutiérrez et al. 1999; Malainey et al. 1999; Regert et al. 2001; Croxton et al. 2010; Abirami & Rajendran 2011; Michalski et al. 2013

Artefact ID	Sample location	Detected compound(s)	Origin/Library match(es) + Reference(s)
GS9	unground surface	not measured	-
GS10	ground surface 1 of 1	**1H-indene, 2,3-dihydro-1,1,3-trimethyl-3-phenyl-**	**present in plants**
		2,4-diphenyl-4-methyl-2(E)-pentene	unknown
		phthalic acid, butyl undecyl ester	unknown
		2-(1,1-dimethylethyl)-4-(1-methyl-1-phenylethyl)-phenol	unknown
		**2,6-bis(1,1-dimethylethyl)-4-(1-methyl-1-phenylethyl)-phenol**	**pentadecanes (15 Carbons), present in burnt plant material; beeswax; nuts and/or seeds:** Kaal et al. 2008, 2009, Lakshmi prava et al. 2012
		**hexadecanoic acid, butyl ester**	**present in plants:** Igwe & Okwu.2013, Sujatha et al. 2014
		**octadecanoic acid, 2-methylpropyl ester**	**possible contamination:** Bai et al. 2014
		**2,4-bis(dimethylbenzyl)-6-t-butylphenol**	**present in plants:** Castrejón et al. 2003
		2,4-bis(1-methyl-1-phenylethyl)-phenol	unknown
		1-hexacosene	unknown
		**azelaic acid**	**degraded fatty acid, present in acne creams, plants + animals:** Nicolet & Liddle 1916; Eerkins 2002; Garelnabi et al. 2010; Al-Shammari et al. 2012
		**hexadecanoic acid**	**degraded fatty acid, present in plants, animals + beeswax; may indicate handling contamination:** Gutiérrez et al. 1999; Malainey et al. 1999; Regert et al. 2001; Croxton et al. 2010; Malarvizhi & Ramakrishnan 2011; Al-Shammari et al. 2012; Lakshmi prava et al. 2012; Abozid et al. 2013; Michalski et al. 2013; Maia & Nunes 2013
		**octadecanoic acid**	**fatty acid, present in plants, animals + beeswax; may indicate handling contamination:** Gutiérrez et al. 1999; Malainey et al. 1999; Regert et al. 2001; Croxton et al. 2010; Abirami & Rajendran 2011; Michalski et al. 2013
GS10	sediment	no compounds detected	-
GS10	unground surface	not measured	-
GS13	ground surface 1 of 1	2,6,10-trimethyldecane	unknown
		**1H-indene, 2,3-dihydro-1,1,3-trimethyl-3-phenyl-**	**present in plants**
		2,4-diphenyl-4-methyl-2(E)-pentene	unknown
		**phthalic acid, octyl tridec-2-yn-1-yl ester**	**present in plants:** Senthilkumar et al. 2012
		**phthaic acid, butyl hexyl ester**	**present in plants:** Dev et al. 2011, Hossain et al. 2011
		2-(1,1-dimethylethyl)-4-(1-methyl-1-phenylethyl)-phenol	unknown
		**2,6-bis(1,1-dimethylethyl)-4-(1-methyl-1-phenylethyl)-phenol**	**pentadecanes (15 Carbons), present in burnt plant material; beeswax; nuts and/or seeds:** Kaal et al. 2008, 2009, Lakshmi prava et al. 2012
		**hexadecanoic acid, butyl ester**	**present in plants:** Igwe & Okwu.2013, Sujatha et al. 2014
		1-hexacosene	unknown
		octadecanoic acid, 2-methylpropoyl ester	unknown
		**2,4-bis(dimethylbenzyl)-6-t-butylphenol**	**present in plants:** Castrejón et al. 2003
		phthalic acid, 2-ethylhexyl neopentyl ester	unknown
GS13	sediment	no compounds detected	-
GS13	unground surface	not measured	-
GS14	ground surface 1 of 3	**1H-indene, 2,3-dihydro-1,1,3-trimethyl-3-phenyl-**	**present in plants**
		2,4-diphenyl-4-methyl-2(E)-pentene	unknown
		naphthalene-2,6-dicarboxylic acid, pentyl ester 4-pentyl-phenyl ester	unknown
		2-tert-butyl-4-methyl-6-(a-methylbenzyl)phenol	unknown
		2-(1,1-dimethylethyl)-4-(1-methyl-1-phenylethyl)-phenol	unknown
		**2,6-bis(1,1-dimethylethyl)-4-(1-methyl-1-phenylethyl)-phenol**	**pentadecanes (15 Carbons); present in burnt plant material; beeswax; nuts and/or seeds:** Kaal et al. 2008, 2009, Lakshmi prava et al. 2012
		**2,4-bis(dimethylbenzyl)-6-t-butylphenol**	**present in plants:** Castrejón et al. 2003
		2,4-bis(1-methyl-1-phenylethyl)-phenol	unknown

Artefact ID	Sample location	Detected compound(s)	Origin/Library match(es) + Reference(s)
		phenol<br>2-hydroxymethylphenol<br>5-methylpentadecane<br>1-acetyl-4-amino-5-ethyl-2,5-dihydro-1H-pyrrole-3-carbonitrile<br>2,4-diphenyl-4-methyl-2(E)-pentene<br>2-phenyl-2-oxophenyl-propane<br>**11-norcannabinol-9-carboxylic acid**<br>Bis(4-hydroxyphenyl)methane<br>Bis(4-hydroxyphenyl)methane<br>5,8,11,14-eicosatetraynoic acid<br>**hexadecanoic acid, 1,1-dimethylethyl ester**<br>**docosahexanoic acid, 1,2,3-propanetriyl ester**<br>2,4-bis(1-methyl-1-phenylethyl)-phenol<br>**2-hydroxypropanoic acid**<br>**hexadecanoic acid**<br>**octadecanoic acid**	unknown<br>unknown<br>unknown<br>unknown<br>unknown<br>unknown<br>**present in plants (bioactive):** Aneela et al. 2014<br>unknown<br>unknown<br>unknown<br>**present in plants:** Prakash et al. 2011<br>**present in plants:** Olutayo et al. 2013<br>unknown<br>**lactic acid, present in plants + animals**<br>**degraded fatty acid, present in plants, animals + beeswax; may indicate handling contamination:** Gutiérrez et al. 1999; Malainey et al. 1999; Regert et al. 2001; Croxton et al. 2010; Malarvizhi & Ramakrishnan 2011; Al-Shammari et al. 2012; Lakshmi prava et al. 2012; Abozid et al. 2013; Michalski et al. 2013; Maia & Nunes 2013<br>**fatty acid, present in plants, animals + beeswax; may indicate handling contamination:** Gutiérrez et al. 1999; Malainey et al. 1999; Regert et al. 2001; Croxton et al. 2010; Abirami & Rajendran 2011; Michalski et al. 2013
GS14	ground surface 2 of 3	not measured	-
GS14	ground surface 3 of 3	not measured	-
GS14	sediment	no compounds detected	-
GS14	unground surface	not measured	-
GS15	ground surface 1 of 1	4,6-dimethyl-2-thioxo-1,2-dihydro-3-pyridinecarbonitrile<br>2,4-diphenyl-4-methyl-2(E)-pentene<br>**2,6-bis(1,1-dimethylethyl)-4-(1-methyl-1-phenylethyl)-phenol**<br>**2,4-bis(dimethylbenzyl)-6-t-butylphenol**<br>2,4-bis(1-methyl-1-phenylethyl)-phenol<br>2,4-bis(1-methyl-1-phenylethyl)-phenol hexadecanoic acid<br>**octadecanoic acid**	unknown<br>unknown<br>**pentadecanes (15 Carbons), present in burnt plant material; beeswax; plant residue inc. nuts and/or seed:** Kaal et al. 2008, 2009, Lakshmi prava et al. 2012<br>**present in plants:** Castrejón et al. 2003<br>unknown<br>unknown<br>**fatty acid, present in plants, animals + beeswax; may indicate handling contamination:** Gutiérrez et al. 1999; Malainey et al. 1999; Regert et al. 2001; Croxton et al. 2010; Abirami & Rajendran 2011; Michalski et al. 2013
GS15	sediment	no compounds detected	-
GS15	unground surface	not measured	-
GS16	ground surface 1 of 2	2,6,10-trimethyltetradecane<br>**1H-indene, 2,3-dihydro-1,1,3-trimethyl-3-phenyl-**<br>2,4-diphenyl-4-methyl-2(E)-pentene<br>**2,6-bis(1,1-dimethylethyl)-4-(1-methyl-1-phenylethyl)-phenol**<br>**Pregan-20-one, 2-hydroxy-5,6-epoxy-15-methyl-**<br>**9-desoxo-9-x-acetoxy-3,8,12-tri-O-acetylingol**<br>**2,4-bis(dimethylbenzyl)-6-t-butylphenol**<br>2,4-bis(1-methyl-1-phenylethyl)-phenol<br>2,4-bis(1-methyl-1-phenylethyl)-phenol	unknown<br>**present in plants**<br>unknown<br>**pentadecanes (15 Carbons); present in burnt plant material; beeswax; nuts and/or seeds:** Kaal et al. 2008, 2009, Lakshmi prava et al. 2012<br>**propolis, present in beeswax (bioactive):** Abozid et al. 2013, Shubharani et al. 2014<br>**present in plants (bioactive); esssential oil of *Mikania scanden*:** Remya & Saj 2013<br>**present in plants:** Castrejón et al. 2003<br>unknown<br>unknown

Artefact ID	Sample location	Detected compound(s)	Origin/Library match(es) + Reference(s)
GS16	ground surface 2 of 2	**1,2-benzisothiazol-3-amine**<br>**phenol**<br>phosphate<br>**2,4,6-trimethyloctane**<br>**dodecanedioic acid**<br><br>2,4-diphenyl-4-methyl-2(E)-pentene<br>**tetradecanoic acid**<br><br><br><br><br><br>**narcissidine-7-one, 1,3-diacetyl-4,12-dihydro-,(1.alpha.,2.beta.,3.alpha.)-**	**present in plants (bioactive):** Priyanka et al. 2014<br>**present in plants e.g., *Ficus*:** Saravanan et al. 2014<br>unknown<br>**undecanes (11 Carbons), present in burnt plant material:** Kaal et al. 2008<br>**sebacic acid, present in plants, honey; may indicate handling contamination,:** Byrdwell & Neff 1998; Eerkens 2002; Senanayake 2006; Lachman et al. 2010<br>unknown<br>**myristic acid, present in plants (e.g., *Calophyllum*); plant oils; animal fats:** Gutiérrez et al. 1999; Maya et al. 2006; Azmat et al. 2010; Ogunlesi et al. 2010; Sutha et al. 2011; Abirami & Rajendran 2011; Fievez et al. 2011; Kale et al. 2011; Malarvizhi & Ramakrishnan 2011; Maruthupandian & Mohan 2011; Saravanan et al. 2013, Al-Shammari et al. 2012; Ertas et al. 2014; Gnanamuthu & Rameshkumar 2014<br>**alkaloid, present in plant (bioactive):** Santana et al. 2008; Bastida et al. 2011; de Andrade et al. 2012
GS16	sediment	no compounds detected	-
GS16	unground surface	not measured	-
GS18	ground surface 1 of 2	2,4-diphenyl-4-methyl-2(E)-pentene<br>**phthalic acid, dibutyl ester**<br><br>**2,6-bis(1,1-dimethylethyl)-4-(1-methyl-1-phenylethyl)-phenol**<br><br>**hexadecanoic acid butyl ester**<br>1-hexacosene<br>**octadecanoic acid butyl ester**<br>**2,4-bis(dimethylbenzyl)-6-t-butylphenol**<br>2,4-bis(1-methyl-1-phenylethyl)-phenol<br>**1H-indene, 2,3-dihydro-1,1,3-trimethyl-3-phenyl-**<br>2,4-diphenyl-4-methyl-2(E)-pentene<br>1,5-cyclooctadiene, 3,4,7,8-tetrakis(1-methylethylidene)-<br>1,5-cyclooctadiene, 3,4,7,8-tetrakis(1-methylethylidene)-<br>hydrazine, N-(3-methylbenzoyl)-N'-(2-nitrobenzoyl)-	unknown<br>**present in plants; can indicate handling contamination:** Zhao & Yang 2008; Al-Shammari et al. 2012; Prasad & Suresh 2012<br>**pentadecanes (15 Carbons), present in burnt plant material; beeswax; nuts and/or seed:** Kaal et al. 2008, 2009, Lakshmi prava et al. 2012<br>**present in plants:** Igwe & Okwu 2013; Sujatha et al. 2014<br>unknown<br>**present in plants:** Nayak et al. 2014<br>**present in plants:** Castrejón et al. 2003<br>unknown<br>**present in plants**<br>unknown<br>unknown<br>unknown<br>unknown
GS18	ground surface 2 of 2	**ethanediol**<br>**phenol**<br>**2-hydroxypropanoic acid**<br>glycerol<br>2-hydroxymethylphenol<br>2,6-ditertbutyl-phenol<br>2,4-diphenyl-4-methyl-2(E)-pentene<br>2-phenyl-2-oxophenyl-propane<br>D-homo-24-nor-17-oxachola-20,22-diene-3,7,16-trione, 14,15:21,23-diepoxy-4,4,8-trimethyl-, (5.alpha.,13.alpha.,14.beta.,15.beta.,17a.alpha.)-<br>phthalic acid, butyl dodecyl ester<br>oxirane, 2,2'-((1-methylethylidene)bis(4,1-phenyleneoxymethylene))bis-<br>oxirane, 2,2'-((1-methylethylidene)bis(4,1-phenyleneoxymethylene))bis-<br>2'-oxophenyl-4'-oxophenyl-methane<br>**2,6-bis(1,1-dimethylethyl)-4-(1-methyl-1-phenylethyl)-phenol**	**ethylene glycol, present in household cleaning products; paints; cosmetics**<br>**present in plants e.g., *Ficus*:** Saravanan et al. 2014<br>**lactic acid, present in plant + animal**<br>unknown<br>unknown<br>unknown<br>unknown<br>unknown<br>unknown<br>unknown<br>unknown<br>unknown<br>unknown<br>unknown<br>**pentadecanes (15 Carbons), present in burnt plant material; beeswax; nuts and/or seed:** Kaal et al. 2008, 2009, Lakshmi prava et al. 2012

Artefact ID	Sample location	Detected compound(s)	Origin/Library match(es) + Reference(s)
		Bis(4-hydroxyphenyl)methane<br>**2,4-bis(dimethylbenzyl)-6-t-butylphenol**	unknown<br>**present in plants:** Castrejón et al. 2003
GS18	sediment	not measured	-
GS18	unground surface	not measured	-
GS19	ground surface 1 of 1	**1H-indene, 2,3-dihydro-1,1,3-trimethyl-3-phenyl-**<br>2,4-diphenyl-4-methyl-2(E)-pentene<br>**2,6-bis(1,1-dimethylethyl)-4-(1-methyl-1-phenylethyl)-phenol**<br><br>1,5-cyclooctadiene, 3,4,7,8-tetrakis(1-methylethylidene)-<br>**hexadecanoic acid butyl ester**<br>**1-heptadecanol acetate**<br>**octadecanoic acid butyl ester**<br>**octadecanoic acid 2-methylpropyl ester**<br>**2,4-bis(dimethylbenzyl)-6-t-butylphenol**<br>**2,6-bis(1,1-dimethylethyl)-4-(1-methyl-1-phenylethyl)-phenol**	**present in plants**<br>unknown<br>**pentadecanes (15 Carbons), present in burnt plant material; beeswax; nuts and/or seeds:** Kaal et al. 2008, 2009, Lakshmi prava et al. 2012<br>unknown<br>**present in plants:** Igwe & Okwu 2013; Sujatha et al. 2014<br>**present in plants:** Wesołowska et al. 2011<br>**present in plants:** Nayak et al. 2014<br>**contamination**: Stringer et al. 2000<br>**present in plants:** Castrejón et al. 2003<br>**pentadecanes (15 Carbons), present in burnt plant material; beeswax; nuts and/or seeds:** Kaal et al. 2008, 2009, Lakshmi prava et al. 2012
GS19	sediment	no compounds detected	-
GS19	unground surface	not measured	-
GS20	ground surface 1 of 1	**phenol**<br>2-hydroxymethylphenol<br>2,6,10-trimethyltetradecane<br>2,4-diphenyl-4-methyl-2(E)-pentene<br>4,4'-(1-methylethylidene)bis(2,6-dimethyl-phenol)<br>**(1,1,2-trimethylpropyl)-benzene**<br>2,4-diphenyl-4-methyl-2(E)-pentene<br>2,4,7,14-tetramethyl-4-vinyl-tricyclo(5.4.3.0(1,8))tetradecan-6-ol<br>2-phenyl-2-oxophenyl-propane<br>2-tert-butyl-4-methyl-6-(a-methylbenzyl)phenol<br>**phthalic acid, isobutyl octadecyl ester**<br>oxirane, 2,2'-((1-methylethylidene)bis(4,1-phenyleneoxymethylene))bis-<br>Bis(4-hydroxyphenyl)methane<br>**2,6-bis(1,1-dimethylethyl)-4-(1-methyl-1-phenylethyl)-phenol**<br><br>dibenzo(b.f)1,5-dioxacyclooctane, 4-methoxy-6,12-(ethylideno)-<br>1,5-cyclooctadiene, 3,4,7,8-tetrakis(1-methylethylidene)-<br>**hexadecanoic acid, 1,1-dimethylethyl ester**<br>**2,4-bis(dimethylbenzyl)-6-t-butylphenol**<br>2,4-bis(1-methyl-1-phenylethyl)-phenol<br>**monolinoleoylglycerol**<br><br><br><br>**hexadecanoic acid**<br><br><br><br>**octadecanoic acid**	**present in plants e.g., *Ficus*:** Saravanan et al. 2014<br>unknown<br>unknown<br>unknown<br>unknown<br>**contamination**<br>unknown<br>unknown<br>unknown<br>unknown<br>**present in plants, e.g., *Solanum*:** Ren & Tian 2012; Chen-xing et al. 2014; Kaushik et al. 2014<br>unknown<br>unknown<br>**pentadecanes (15 Carbons), present in burnt plant material; beeswax; nuts and/or seeds:** Kaal et al. 2008, 2009, Lakshmi prava et al. 2012<br>unknown<br>unknown<br>**present in plants:** Prakash et al. 2011<br>**present in plants:** Castrejón et al. 2003<br>unknown<br>**present in plants:** Bhuiyan et al. 2009; Merlin et al. 2009; Lakshmi prava & RajaLakshmi prava 2011; Malarvizhi & Ramakrishnan 2011; Murugesan & Panneerselvam 2013; Sheela & Uthayakumari 2013<br>**degraded fatty acid, present in plants, animals + beeswax; may indicate handling contamination:** Gutiérrez et al. 1999; Malainey et al. 1999; Regert et al. 2001; Croxton et al. 2010; Malarvizhi & Ramakrishnan 2011; Al-Shammari et al. 2012; Lakshmi prava et al. 2012; Abozid et al. 2013; Michalski et al. 2013; Maia & Nunes 2013

Artefact ID	Sample location	Detected compound(s)	Origin/Library match(es) + Reference(s)
			**fatty acid, present in plants, animals + beeswax; may indicate handling contamination:** Gutiérrez et al. 1999; Malainey et al. 1999; Regert et al. 2001; Croxton et al. 2010; Abirami & Rajendran 2011; Michalski et al. 2013
GS20	sediment	no compounds detected	-
GS20	unground surface	not measured	-
GS21	ground surface 1 of 1	**7-methylhexadecane**<br><br>**1H-indene, 2,3-dihydro-1,1,3-trimethyl-3-phenyl-**<br>**serverogenin acetate**<br>2,4,4,6-tetramethyl-6-phenyl-1-heptene<br>2,4-diphenyl-4-methyl-2(E)-pentene<br>androst-5,7-dien-3-ol-17-one, acetate<br>**Pregan-20-one, 2-hydroxy-5,6-epoxy-15-methyl-**<br>phthalic acid, butyl tridec-2-yn-1-yl ester<br>**phenylalanine, 4-amino-N-t-butyloxycarbonyl-, t-butyl ester**<br>4,6-bis(t-butyl)-2-(dimethylbenzyl)phenol<br>**2,6-bis(1,1-dimethylethyl)-4-(1-methyl-1-phenylethyl)-phenol**<br><br><br>1,5-cyclooctadiene, 3,4,7,8-tetrakis(1-methylethylidene)-<br>2,6-dimethyl-4,4-tetramethylene-1,4-dihydropyridine-3,5-dicarbonitrile<br>**2,4-bis(dimethylbenzyl)-6-t-butylphenol**<br>2,4-diphenyl-4-methyl-2(E)-pentene<br>androst-5,7-dien-3-ol-17-one, acetate<br>**hexadecanoic acid**<br><br><br><br><br>**octadecanoic acid**	**heptadecanes (17 Carbons), present in burnt plant material, beeswax:** Kaal et al. 2008, 2009, Maia & Nunes 2013, Regert et al. 2001<br>**present in plants**<br>**present in plants, e.g., *Trichilia* sp.:** Senthilkumar et al. 2012<br>unknown<br>unknown<br>unknown<br>**propolis, present in beeswax (bioactive):** Abozid et al. 2013, Shubharani et al. 2014<br>unknown<br>**amino acid derivative**<br>unknown<br>**pentadecanes (15 Carbons), present in burnt plant material; beeswax; plant residue inc. nuts and/or seed:** Kaal et al. 2008, 2009, Lakshmi prava et al. 2012<br>unknown<br>unknown<br>**present in plants:** Castrejón et al. 2003<br>unknown<br>unknown<br>**degraded fatty acid, present in plants, animals + beeswax; may indicate handling contamination:** Gutiérrez et al. 1999; Malainey et al. 1999; Regert et al. 2001; Croxton et al. 2010; Malarvizhi & Ramakrishnan 2011; Al-Shammari et al. 2012; Lakshmi prava et al. 2012; Abozid et al. 2013; Michalski et al. 2013; Maia & Nunes 2013<br>**fatty acid, present in plants, animals + beeswax; may indicate handling contamination:** Gutiérrez et al. 1999; Malainey et al. 1999; Regert et al. 2001; Croxton et al. 2010; Abirami & Rajendran 2011; Michalski et al. 2013
GS21	sediment	phthalic acid, butyl 2-methylpropyl ester<br>**1,3-dioxane, 4-(hexadecyloxy)-2-pentadecyl-**	unknown<br>**present in plants:** Salem et al. 2011
GS21	unground surface	not measured	-
GS22	ground surface 1 of 1	**1H-indene, 2,3-dihydro-1,1,3-trimethyl-3-phenyl-**<br>2,4-diphenyl-4-methyl-2(E)-pentene<br>**2,6-bis(1,1-dimethylethyl)-4-(1-methyl-1-phenylethyl)-phenol**<br><br>1H-indole-2-carboxylic acid, 3-methyl-4-oxo-6-(3,4,5-trimethoxyphenyl)-4,5,6,7-tetrahydro-, ethyl ester<br>**2,4-bis(dimethylbenzyl)-6-t-butylphenol**<br>2H,8H-benzo(1,2-b:5,4-b')dipyran-10-propanol, 5-methoxy-2,2,8,8-tetramethyl-<br>2,4-bis(1-methyl-1-phenylethyl)-phenol<br>hexadecane	**present in plants**<br>unknown<br>**pentadecanes (15 Carbons), present in burnt plant material; beeswax; nuts and/or seed:** Kaal et al. 2008, 2009, Lakshmi prava et al. 2012<br>unknown<br>unknown<br>**present in plants:** Castrejón et al. 2003<br>unknown<br>unknown<br>unknown
GS22	sediment	no compounds detected	-
GS22	unground surface	not measured	-
GS23	ground surface 1 of 2	**5-methyl-tetradecane**	**pentadecanes (15 Carbons), present in burnt plant material, beeswax:** Kaal et al. 2008, 2009, Lakshmi prava et al. 2012

Artefact ID	Sample location	Detected compound(s)	Origin/Library match(es) + Reference(s)
		**1H-indene, 2,3-dihydro-1,1,3-trimethyl-3-phenyl-**	**present in plants**
		benzenepropanoic acid	unknown
		2,4-diphenyl-4-methyl-2(E)-pentene	unknown
		**2,6-bis(1,1-dimethylethyl)-4-(1-methyl-1-phenylethyl)-phenol**	**pentadecanes (15 Carbons), present in burnt plant material; beeswax; nuts and/or seed:** Kaal et al. 2008, 2009, Lakshmi prava et al. 2012
		cholestan-8,24-dien-3-ol, 4-methyl-, (3.beta.,4.alpha.)-	unknown
		**Pregan-20-one, 2-hydroxy-5,6-epoxy-15-methyl-**	**propolis, present in beeswax (bioactive):** Abozid et al. 2013, Shubharani et al. 2014
		6-(7-nitrobenzofurazan-4-yl)amino-morphinan-4,5-epoxy-3,6-di-ol	unknown
		**2,4-bis(dimethylbenzyl)-6-t-butylphenol**	**present in plants:** Castrejón et al. 2003
		2,4-bis(1-methyl-1-phenylethyl)-phenol	unknown
		**2,6-dimethyl-heptadecane**	**nonadecanes (19 Carbons), present in burnt plant material, beeswax:** Kaal et al. 2008, 2009, Lakshmi prava et al. 2012
		2,4-diphenyl-4-methyl-2(E)-pentene	unknown
		4,4'-(1-methylethylidene)bis(2,6-dimethyl-phenol)	unknown
		**phthalic acid, butyl nonyl ester**	**contamination**
		**11-norcannabinol-9-carboxylic acid**	**present in plants (bioactive):** Aneela et al. 2014
		**4H-1-benzopyran-4-one, 5,7-dihydroxy-2-(3,4,5-trimethoxyphenyl)-**	**flavone**
		trans-1,1'-bibenzoindanylidene	unknown
		1,2-propanediol, 3-(octadecyloxy)- diacetate	unknown
		**serverogenin acetate**	**present in plants, e.g., *Trichilia* sp.:** Senthilkumar et al. 2012
		2,4-bis(1-methyl-1-phenylethyl)-phenol	unknown
		phosphate	unknown
		**heptanedioic acid**	**pimelic acid**
		**octanedioic acid**	**suberic acid, present in plants:** Yayli et al 2001; Eerkins 2002; Ertas et al. 2014
		**azelaic acid**	**degraded fatty acid, present in acne creams, plants + animals:** Nicolet & Liddle 1916; Eerkins 2002; Garelnabi et al. 2010; Al-Shammari et al. 2012
		**decanedioic acid**	**fatty acid, present in plants, animals, beeswax, can indicate handling contamination:** Gutiérrez et al. 1999; Malainey et al. 1999; Regert et al. 2001; Croxton et al. 2010; Malarvizhi & Ramakrishnan 2011; Al-Shammari et al. 2012; Lakshmi prava et al. 2012; Abozid et al. 2013; Michalski et al. 2013; Maia & Nunes 2013
		5,8,11-eicosatriynoic acid	unknown
		**hexadecanoic acid**	**degraded fatty acid, present in plants, animals + beeswax; may indicate handling contamination:** Gutiérrez et al. 1999; Malainey et al. 1999; Regert et al. 2001; Croxton et al. 2010; Malarvizhi & Ramakrishnan 2011; Al-Shammari et al. 2012; Lakshmi prava et al. 2012; Abozid et al. 2013; Michalski et al. 2013; Maia & Nunes 2013
		androst-4-ene-3,20-dione, 11,16,22-triacetoxy-	unknown
		9,12-octadecadienoic acid	unknown
		**octadecanoic acid**	**fatty acid, present in plants, animals + beeswax; may indicate handling contamination:** Gutiérrez et al. 1999; Malainey et al. 1999; Regert et al. 2001; Croxton et al. 2010; Abirami & Rajendran 2011; Michalski et al. 2013
		9,12-octadecadienoic acid	unknown
		9,12-octadecadienoic acid	unknown
		eicosanol	unknown
		**eicosanoic acid**	**arachidic acid, present in propolis, plants including seeds; nuts (e.g., *Macadamia* sp.); animal:** Abozid et al. 2013, Alhassanm et al. 2014, Gaikwad et al. 2011, Igwe & Okwu 2013, Refaat et al. 2013, Sáez et al. 2014, Suseno et al. 2014
		**octadecandioic acid**	

Artefact ID	Sample location	Detected compound(s)	Origin/Library match(es) + Reference(s)
		**docosanoic acid**<br>monostearin	**present in plants, e.g., *Pandanus;* can indicate handling contamination;** Judefeind et al. 2008, Mahalingam et al. 2012<br>**behenic acid, present in plants including seeda and nuts (*Macadamia* sp):** Ertas et al. 2014, Gaikwad et al. 2011, Igwe & Okwu 2013, Makhija et al. 2010, Sáez et al. 2014<br>unknown
GS23	ground surface 2 of 2	not measured	-
GS23	sediment	octadecanol<br>**hexadecanoic acid, 1,1-dimethylethyl ester**<br>tetradecan-6-ol	unknown<br>**present in plants:** Prakash et al. 2011<br>unknown
GS23	unground surface	not measured	-
GS24	ground surface 1 of 2	2,4-diphenyl-4-methyl-2(E)-pentene<br>2-(1,1-dimethylethyl)-4-(1-methyl-1-phenylethyl)-phenol<br>**2,6-bis(1,1-dimethylethyl)-4-(1-methyl-1-phenylethyl)-phenol**<br>**2,4-bis(dimethylbenzyl)-6-t-butylphenol**<br>2,4-bis(1-methyl-1-phenylethyl)-phenol	unknown<br>unknown<br>**pentadecanes (15 Carbons), present in burnt plant material; beeswax; nuts and/or seed:** Kaal et al. 2008, 2009, Lakshmi prava et al. 2012<br>**present in plants:** Castrejón et al. 2003<br>unknown
GS24	ground surface 2 of 2	4,6-dimethyl-2-thioxo-1,2-dihydro-3-pyridinecarbonitrile<br>2,4-diphenyl-4-methyl-2(E)-pentene<br>**2,6-bis(1,1-dimethylethyl)-4-(1-methyl-1-phenylethyl)-phenol**<br>**2,4-bis(dimethylbenzyl)-6-t-butylphenol**<br>2,4-bis(1-methyl-1-phenylethyl)-phenol<br>phosphate<br>**octanedioic acid**<br>**azelaic acid**	unknown<br>unknown<br>**pentadecanes (15 Carbons), present in burnt plant material; beeswax; plant residue inc. nuts and/or seed:** Kaal et al. 2008, 2009, Lakshmi prava et al. 2012<br>**present in plants:** Castrejón et al. 2003<br>unknown<br>unknown<br>**suberic acid, present in plant:** Yayli et al 2001; Eerkins 2002; Ertas et al. 2014<br>**degraded fatty acid, present in acne creams, plants + animals:** Nicolet & Liddle 1916; Eerkins 2002; Garelnabi et al. 2010; Al-Shammari et al. 2012
GS24	sediment	no compounds detected	-
GS24	unground surface	not measured	-
GS26	ground surface 1 of 1	**1H-indene, 2,3-dihydro-1,1,3-trimethyl-3-phenyl-**<br>2,4-diphenyl-4-methyl-2(E)-pentene<br>**2,4-bis(dimethylbenzyl)-6-t-butylphenol**<br>2,4-bis(1-methyl-1-phenylethyl)-phenol<br>**2,6,11-trimethyl-dodecane**<br>hexadecane<br>2,4-diphenyl-4-methyl-2(E)-pentene<br>**2,6-bis(1,1-dimethylethyl)-4-(1-methyl-1-phenylethyl)-phenol**<br>2,4-bis(1-methyl-1-phenylethyl)-phenol<br>**azelaic acid**	**present in plants**<br>unknown<br>**present in plants:** Castrejón et al. 2003<br>unknown<br>**heptadecanes (17 Carbons), present in burnt plant material, beeswax:** Kaal et al. 2008; 2009; Regert et al. 2001; Maia & Nunes 2013<br>unknown<br>unknown<br>**pentadecanes (15 Carbons), present in burnt plant material; beeswax; nuts and/or seeds:** Kaal et al. 2008, 2009, Lakshmi prava et al. 2012<br>unknown<br>**degraded fatty acid, present in acne creams, plants + animals:** Nicolet & Liddle 1916; Eerkins 2002; Garelnabi et al. 2010; Al-Shammari et al. 2012
GS26	sediment	no compounds detected	-
GS26	unground surface	not measured	-
GS27	ground surface 1 of 1	2-hydroxymethylphenol<br>2,4-diphenyl-4-methyl-2(E)-pentene<br>**7-dehydrocholexteryl isocaproate**<br>**Pregan-20-one, 2-hydroxy-5,6-epoxy-15-methyl-**	unknown<br>unknown<br>**animal residue: milk, skin**<br>**propolis, present in beeswax (bioactive):** Abozid et al. 2013, Shubharani et al. 2014

Artefact ID	Sample location	Detected compound(s)	Origin/Library match(es) + Reference(s)
		2-phenyl-2-oxophenyl-propane	unknown
		**phenylalanine, 4-amino-N-t-butyloxycarbonyl-, t-butyl ester**	**amino acid derivative**
		**narcissidine-7-one, 1,3-diacetyl-4,12-dihydro-,(1.alpha.,2.beta.,3.alpha.)-**	**alkaloid, present in toxic plants (bioactive):** Santana et al. 2008; Bastida et al. 2011; de Andrade et al. 2012
		**11-norcannabinol-9-carboxylic acid**	**present in plants (bioactive):** Aneela et al. 2014
		Bis(4-hydroxyphenyl)methane	unknown
		**2,6-bis(1,1-dimethylethyl)-4-(1-methyl-1-phenylethyl)-phenol**	**pentadecanes (15 Carbons); present in burnt plant material; beeswax; nuts and/or seed:** Kaal et al. 2008, 2009, Lakshmi prava et al. 2012
		**2,4-bis(dimethylbenzyl)-6-t-butylphenol**	**present in plants:** Castrejón et al. 2003
GS27	sediment	octadecanol	unknown
		**octadecanoic acid**	**fatty acid, present in plants, animals + beeswax; may indicate handling contamination:** Gutiérrez et al. 1999; Malainey et al. 1999; Regert et al. 2001; Croxton et al. 2010; Abirami & Rajendran 2011; Michalski et al. 2013
GS27	unground surface	not measured	-
GS28	ground surface 1 of 1	2,4-diphenyl-4-methyl-2(E)-pentene	unknown
		2,4-diphenyl-4-methyl-2(E)-pentene	unknown
		**2,6-bis(1,1-dimethylethyl)-4-(1-methyl-1-phenylethyl)-phenol**	**pentadecanes (15 Carbons); present in burnt plant material; beeswax; nuts and/or seed:** Kaal et al. 2008, 2009, Lakshmi prava et al. 2012
		**2,4-bis(dimethylbenzyl)-6-t-butylphenol**	**present in plants:** Castrejón et al. 2003
		2,4-bis(1-methyl-1-phenylethyl)-phenol	unknown
		2,4-diphenyl-4-methyl-2(E)-pentene	unknown
		2,6-di-tert-butyl-4-(2,4-dimethylbenzyl)phenol	unknown
		phosphate	unknown
		**octanedioic acid**	**suberic acid, present in plant:** Yayli et al 2001; Eerkins 2002; Ertas et al. 2014
		**azelaic acid**	**degraded fatty acid, present in acne creams, plants + animals:** Nicolet & Liddle 1916; Eerkins 2002; Garelnabi et al. 2010; Al-Shammari et al. 2012
GS28	sediment	**octadecanoic acid**	**fatty acid, present in plants, animals + beeswax; may indicate handling contamination:** Gutiérrez et al. 1999; Malainey et al. 1999; Regert et al. 2001; Croxton et al. 2010; Abirami & Rajendran 2011; Michalski et al. 2013
GS28	unground surface	no compounds detected	-
GS29	ground surface 1 of 1	**1H-indene, 2,3-dihydro-1,1,3-trimethyl-3-phenyl-**	**present in plants**
		2,4-diphenyl-4-methyl-2(E)-pentene	unknown
		**2,6-bis(1,1-dimethylethyl)-4-(1-methyl-1-phenylethyl)-phenol**	**pentadecanes (15 Carbons); present in burnt plant material; beeswax; nuts and/or seed:** Kaal et al. 2008, 2009, Lakshmi prava et al. 2012
		**2,4-bis(dimethylbenzyl)-6-t-butylphenol**	**present in plants:** Castrejón et al. 2003
		2,4-bis(1-methyl-1-phenylethyl)-phenol	unknown
		**azelaic acid**	**degraded fatty acid, present in acne creams, plants + animals:** Nicolet & Liddle 1916; Eerkins 2002; Garelnabi et al. 2010; Al-Shammari et al. 2012
GS29	sediment	no compounds detected	-
GS29	unground surface	not measured	-
GS30	ground surface 1 of 1	2,4-diphenyl-4-methyl-2(E)-pentene	unknown
		**2,6-bis(1,1-dimethylethyl)-4-(1-methyl-1-phenylethyl)-phenol**	**pentadecanes (15 Carbons); present in burnt plant material; beeswax; nuts and/or seed:** Kaal et al. 2008, 2009, Lakshmi prava et al. 2012
		**hexadecanoic acid, 1,1-dimethylethyl ester**	**present in plants:** Prakash et al. 2011
		**2,4-bis(dimethylbenzyl)-6-t-butylphenol**	**present in plants:** Castrejón et al. 2003
		**azelaic acid**	**degraded fatty acid, present in acne creams, plants + animals:** Nicolet & Liddle 1916; Eerkins 2002; Garelnabi et al. 2010; Al-Shammari et al. 2012

Artefact ID	Sample location	Detected compound(s)	Origin/Library match(es) + Reference(s)
GS30	sediment	no compounds detected	-
GS30	unground surface	not measured	-
GS31	ground surface 1 of 1	2,4-diphenyl-4-methyl-2(E)-pentene<br>2,6-di-tert-butyl-4-(2,4-dimethylbenzyl)phenol<br>**2,4-bis(dimethylbenzyl)-6-t-butylphenol**<br>**1-ethyl-4-methylbenzene**<br><br>tetradecan-3-ol<br>5-hydroxy-5-methyl-2-phenyl-3-isoxazolidinone<br>**azelaic acid**<br><br>**hexadecanoic acid**<br><br><br><br>**octadecanoic acid**	unknown<br>unknown<br>**present in plants:** Castrejón et al. 2003<br>**present in plants, including seed and/or nut:** de Lacy Costello et al. 2001; Ciganek et al. 2007; Janakiraman et al. 2012; Omikorede et al. 2012.<br>unknown<br>unknown<br>**degraded fatty acid, present in acne creams, plants + animals:** Nicolet & Liddle 1916; Eerkins 2002; Garelnabi et al. 2010; Al-Shammari et al. 2012<br>**degraded fatty acid, present in plants, animals + beeswax; may indicate handling contamination:** Gutiérrez et al. 1999; Malainey et al. 1999; Regert et al. 2001; Croxton et al. 2010; Malarvizhi & Ramakrishnan 2011; Al-Shammari et al. 2012; Lakshmi prava et al. 2012; Abozid et al. 2013; Michalski et al. 2013; Maia & Nunes 2013<br>**fatty acid, present in plants, animals + beeswax; may indicate handling contamination:** Gutiérrez et al. 1999; Malainey et al. 1999; Regert et al. 2001; Croxton et al. 2010; Abirami & Rajendran 2011; Michalski et al. 2013
GS31	sediment	no compounds detected	-
GS31	unground surface	not measured	-
GS32	ground surface 1 of 2	**1H-indene, 2,3-dihydro-1,1,3-trimethyl-3-phenyl-**<br>2-methyl-2-phenyl-tridecane<br>2,4-diphenyl-4-methyl-2(E)-pentene<br><br>**octadecanoic acid, ethyl ester**<br><br>2,6-di-tert-butyl-4-(2,4-dimethylbenzyl)phenol<br>1,5-cyclooctadiene, 3,4,7,8-tetrakis(1-methylethylidene)-<br>**2,4-bis(dimethylbenzyl)-6-t-butylphenol**<br>**1H-indene, 2,3-dihydro-1,1,3-trimethyl-3-phenyl-**<br>1,1'-(3,3-dimethyl-1-butenylidene)bis-benzene<br>**2,6-bis(1,1-dimethylethyl)-4-(1-methyl-1-phenylethyl)-phenol**<br><br>**azelaic acid**	**present in plants**<br>unknown<br>unknown<br>**present in plants, e.g., *Cissus*; insects:** Kumar et al. 2012; Babu et al. 2014; Lacheva 2014; Sivagurunathan 2014<br>unknown<br>unknown<br>**present in plants:** Castrejón et al. 2003<br>**present in plants**<br>unknown<br>**pentadecanes (15 Carbons), present in burnt plant material; beeswax; nuts and/or seeds:** Kaal et al. 2008, 2009, Lakshmi prava et al. 2012<br>**degraded fatty acid, present in acne creams, plants + animals:** Nicolet & Liddle 1916; Eerkins 2002; Garelnabi et al. 2010; Al-Shammari et al. 2012
GS32	ground surface 2 of 2	no compounds detected	-
GS32	sediment	no compounds detected	-
GS32	unground surface	not measured	-
GS33	ground surface 1 of 1	2,4-diphenyl-4-methyl-2(E)-pentene<br>**2,6-bis(1,1-dimethylethyl)-4-(1-methyl-1-phenylethyl)-phenol**<br><br>**2,4-bis(dimethylbenzyl)-6-t-butylphenol**<br>phosphate<br>**azelaic acid**	unknown<br>**pentadecanes (15 Carbons); present in burnt plant material; beeswax; nuts and/or seed:** Kaal et al. 2008, 2009, Lakshmi prava et al. 2012<br>**present in plants:** Castrejón et al. 2003<br>unknown<br>**degraded fatty acid, present in acne creams, plants + animals:** Nicolet & Liddle 1916; Eerkins 2002; Garelnabi et al. 2010; Al-Shammari et al. 2012
GS33	sediment	no compounds detected	-
GS33	unground surface	not measured	-
GS35	ground surface 1 of 2	Bis(4-hydroxyphenyl)methane	unknown

Artefact ID	Sample location	Detected compound(s)	Origin/Library match(es) + Reference(s)
		**azelaic acid**	**degraded fatty acid, present in acne creams, plants + animals:** Nicolet & Liddle 1916; Eerkins 2002; Garelnabi et al. 2010; Al-Shammari et al. 2012
GS35	ground surface 2 of 2	2-phenyl-2-oxophenyl-propane	unknown
		**hexadecanoic acid**	**degraded fatty acid, present in plants, animals + beeswax; may indicate handling contamination:** Gutiérrez et al. 1999; Malainey et al. 1999; Regert et al. 2001; Croxton et al. 2010; Malarvizhi & Ramakrishnan 2011; Al-Shammari et al. 2012; Lakshmi prava et al. 2012; Abozid et al. 2013; Michalski et al. 2013; Maia & Nunes 2013
			unknown
		pentadecan-2-ol	unknown
		(4-hydroxyphenyl)-(2-hydroxyphenyl)-methane	unknown
		Bis(4-hydroxyphenyl)methane	**unknown, bioactive**
		**androsta-3,5-dien-3-ol**	unknown
		3,5-bis(4-(1,1-dimethylethyl)phenyl)-2,3-dihydro-1H-indene-1-one	unknown
		2,4-imidazolidinedione, 5-(3,4-dihydroxy-phenyl)-3-methyl-5-phenyl-	**present in plants:** Castrejón et al. 2003
		**2,4-bis(dimethylbenzyl)-6-t-butylphenol**	**degraded fatty acid, present in acne creams, plants + animals:** Nicolet & Liddle 1916;
		**azelaic acid**	Eerkins 2002; Garelnabi et al. 2010; Al-Shammari et al. 2012
GS35	sediment	no compounds detected	-
GS35	unground surface	not measured	-
GS36	ground surface 1 of 2	no compounds detected	-
GS36	ground surface 2 of 2	acetamide	unknown
		5-(3-benzylamino-2-hydroxypropoxy)naphtho(1,2-b)thiophene	unknown
		1-ethyl-2-methyl-benzene	unknown
		1-ethyl-4-methyl-benzene	unknown
		phosphate	unknown
		2,4-imidazolidinedione, 5-(3,4-dihydroxy-phenyl)-3-methyl-5-phenyl-	unknown
		**octanedioic acid**	**suberic acid, present in plant:** Yayli et al 2001; Eerkins 2002; Ertas et al. 2014
		azelaic acid	unknown
		2,4-imidazolidinedione, 5-(3,4-dihydroxy-phenyl)-3-methyl-5-phenyl-	unknown
		4,4'-(1-methylethylidene)bis(2,6-dimethyl-phenol)	unknown
		**hexadecanoic acid**	**degraded fatty acid, present in plants, animals + beeswax; may indicate handling contamination:** Gutiérrez et al. 1999; Malainey et al. 1999; Regert et al. 2001; Croxton et al. 2010; Malarvizhi & Ramakrishnan 2011; Al-Shammari et al. 2012; Lakshmi prava et al. 2012; Abozid et al. 2013; Michalski et al. 2013; Maia & Nunes 2013
		3,9.beta.;14,15-diepoxypregn-16-en-20-one, 3,11.beta.,18-triacetoxy-	unknown
		2-(4-methoxy-phenyl)-6-p-tolyl-pyridine	unknown
		**androsta-3,5-dien-3-ol**	**unknown, bioactive**
		**octadecanoic acid**	**fatty acid, present in plants, animals + beeswax; may indicate handling contamination:** Gutiérrez et al. 1999; Malainey et al. 1999; Regert et al. 2001; Croxton et al. 2010; Abirami & Rajendran 2011; Michalski et al. 2013
		eicosanol	unknown
		**eicosanoic acid**	**arachidic acid, present in propolis, plants including seeds + nuts (e.g., *Macadamia* sp.); animal residues:** Abozid et al. 2013, Alhassanm et al. 2014, Gaikwad et al. 2011, Igwe & Okwu 2013, Refaat et al. 2013, Sáez et al. 2014, Suseno et al. 2014
		**1-monolinoleoylglycerol**	**present in plants e.g, *Calophyllum:*** Bhuiyan et al. 2009; Merlin et al. 2009; Lakshmi & Rajalakshmi 2011; Malarvizhi & Ramakrishnan 2011; Murugesan & Panneerselvam 2013; Sheela & Uthayakumari 2013
		5,8,11-eicosatriynoic acid	unknown

Artefact ID	Sample location	Detected compound(s)	Origin/Library match(es) + Reference(s)
		**azelaic acid**	**degraded fatty acid, present in acne creams, plants + animals:** Nicolet & Liddle 1916; Eerkins 2002; Garelnabi et al. 2010; Al-Shammari et al. 2012
GS36	sediment	no compounds detected	-
GS36	unground surface	not measured	-
GS37	ground surface 1 of 1	**2-ethylhexanoic acid**<br><br>**azelaic acid**<br><br>**monolinoleoylglycerol**<br><br><br>**hexadecanoic acid**<br><br><br><br>**octadecanoic acid**<br><br><br>**2',6'-dihydroxyacetophenone**	**present in plants; honey:** Fiehn et al. 2000, Jerković & Marijanović 2010, Hammami et al. 2011, Wang et al. 2012<br>**degraded fatty acids, present in acne creams, plants + animals:** Nicolet & Liddle 1916; Eerkins 2002; Garelnabi et al. 2010; Al-Shammari et al. 2012<br>**present in plants:** Bhuiyan et al. 2009; Merlin et al. 2009; Lakshmi prava & RajaLakshmi prava 2011; Malarvizhi & Ramakrishnan 2011; Murugesan & Panneerselvam 2013; Sheela & Uthayakumari 2013<br>**degraded fatty acid, present in plants, animals + beeswax; may indicate handling contamination:** Gutiérrez et al. 1999; Malainey et al. 1999; Regert et al. 2001; Croxton et al. 2010; Malarvizhi & Ramakrishnan 2011; Al-Shammari et al. 2012; Lakshmi prava et al. 2012; Abozid et al. 2013; Michalski et al. 2013; Maia & Nunes 2013<br>**fatty acid, present in plants, animals + beeswax; may indicate handling contamination:** Gutiérrez et al. 1999; Malainey et al. 1999; Regert et al. 2001; Croxton et al. 2010; Abirami & Rajendran 2011; Michalski et al. 2013<br>**present in plants:** Ceciliaet al. 2012
GS37	sediment	no compounds detected	-
GS37	unground surface	not measured	-
GS38	ground surface 1 of 2	**azelaic acid**	**degraded fatty acids, present in acne creams, plants + animals:** Nicolet & Liddle 1916; Eerkins 2002; Garelnabi et al. 2010; Al-Shammari et al. 2012
GS38	ground surface 2 of 2	**azelaic acid**	**degraded fatty acids, present in acne creams, plants + animals:** Nicolet & Liddle 1916; Eerkins 2002; Garelnabi et al. 2010; Al-Shammari et al. 2012
GS38	sediment	no compounds detected	-
GS38	unground surface	not measured	-
GS39	ground surface 1 of 1	acetamide<br>propylbenzene<br>1-ethyl-2-methyl-benzene<br>**isopropylbenzene**<br>**2-hydroxypropanoic acid**<br>**octadecanoic acid**	unknown<br>unknown<br>unknown<br>**cumene**<br>**lactic acid, present in plants + animals**<br>**fatty acid, present in plants, animals + beeswax; may indicate handling contamination:** Gutiérrez et al. 1999; Malainey et al. 1999; Regert et al. 2001; Croxton et al. 2010; Abirami & Rajendran 2011; Michalski et al. 2013
GS39	sediment	no compounds detected	-
GS39	unground surface	not measured	-
GS40	ground surface 1 of 1	no compounds detected	-
GS40	sediment	no compounds detected	-
GS40	unground surface	not measured	-
GS41	ground surface 1 of 1	no compounds detected	-
GS41	sediment	no compounds detected	-
GS41	unground surface	not measured	-
GS42	ground surface 1 of 1	phosphate	unknown
GS42	sediment	**hexadecanoic acid, 1,1-dimethylethyl ester**<br>**octadecanoic acid butyl ester**	**present in plants:** Prakash et al. 2011<br>**present in plants:** Nayak et al. 2014

Artefact ID	Sample location	Detected compound(s)	Origin/Library match(es) + Reference(s)
		phthalic acid, decyl 2-ethylhexyl ester	unknown
GS42	unground surface	not measured	-
GS43	ground surface 1 of 1	no compounds detected	-
GS43	sediment	no compounds detected	-
GS43	unground surface	not measured	-
GS44	ground surface 1 of 1	**azelaic acid**	**degraded fatty acid, present in acne creams, plants + animals:** Nicolet & Liddle 1916; Eerkins 2002; Garelnabi et al. 2010; Al-Shammari et al. 2012
GS44	sediment	**azelaic acid**	**degraded fatty acid, present in acne creams, plants + animals:** Nicolet & Liddle 1916; Eerkins 2002; Garelnabi et al. 2010; Al-Shammari et al. 2012
GS44	unground surface	not measured	-
GS45	ground surface 1 of 1	no compounds detected	-
GS45	sediment	**Bis(4-hydroxyphenyl)propane**<br>**azelaic acid**	**present in plants (bioactive):** Kim et al. 2004<br>**degraded fatty acid, present in acne creams, plants + animals:** Nicolet & Liddle 1916; Eerkins 2002; Garelnabi et al. 2010; Al-Shammari et al. 2012
GS45	unground surface	not measured	-
GS46	ground surface 1 of 2	**benzoic acid**<br><br>**azelaic acid**<br><br>**hexadecanoic acid**	**present in plants (antibacterial), e.g., *Petalostigma, Ficus:*** Fountain et al. 1995; Cock & Kalt 2012; Kalt & Cock 2014; Jeong et al. 2014; Saravanan et al. 2014<br>**degraded fatty acid, present in acne creams, plants + animals:** Nicolet & Liddle 1916; Eerkins 2002; Garelnabi et al. 2010; Al-Shammari et al. 2012<br>**degraded fatty acid, present in plants, animals + beeswax; may indicate handling contamination:** Gutiérrez et al. 1999; Malainey et al. 1999; Regert et al. 2001; Croxton et al. 2010; Malarvizhi & Ramakrishnan 2011; Al-Shammari et al. 2012; Lakshmi prava et al. 2012; Abozid et al. 2013; Michalski et al. 2013; Maia & Nunes 2013
GS46	ground surface 2 of 2	no compounds detected	-
GS46	sediment	**azelaic acid**	**degraded fatty acid, present in acne creams, plants + animals:** Nicolet & Liddle 1916; Eerkins 2002; Garelnabi et al. 2010; Al-Shammari et al. 2012
GS46	unground surface	not measured	-
GS47	ground surface 1 of 1	Bis(4-hydroxyphenyl)methane<br>phosphate	unknown<br>unknown
GS47	unground surface	not measured	-
GS48	ground surface 1 of 1	no compounds detected	-
GS48	sediment	no compounds detected	-
GS48	unground surface	not measured	-
GS49	ground surface 1 of 1	**azelaic acid**	**degraded fatty acid, present in acne creams, plants + animals:** Nicolet & Liddle 1916; Eerkins 2002; Garelnabi et al. 2010; Al-Shammari et al. 2012
GS49	sediment	no compounds detected	-
GS49	unground surface	not measured	-
GS50	ground surface 1 of 1	no compounds detected	-
GS50	sediment	**dodecanoic acid, 2,3-bis(acetyloxy)propyl ester**	**present in plants:** Sodipo et al. 2012
GS50	unground surface	not measured	-
GS53	ground surface 1 of 1	alanine<br>**hexadecanoic acid**<br><br><br><br>**ascorbic acid 2,6-dihexadecanoate**	unknown<br>**degraded fatty acid, present in plants, animals + beeswax; may indicate handling contamination:** Gutiérrez et al. 1999; Malainey et al. 1999; Regert et al. 2001; Croxton et al. 2010; Malarvizhi & Ramakrishnan 2011; Al-Shammari et al. 2012; Lakshmi prava et al. 2012; Abozid et al. 2013; Michalski et al. 2013; Maia & Nunes 2013<br>**vitamin C fatty ester**

Artefact ID	Sample location	Detected compound(s)	Origin/Library match(es) + Reference(s)
		16-methyl heptadecanoic acid<br>Triacontyl acetate<br>hexadecyl nonyl ether<br>**Phenol, 2,2'-methylenebis[6-(1,1-dimethylethyl)-4-methyl-**<br>**hexadecanoic acid, 2-hydroxy-1-(hydroxymethyl)ethyl ester**<br>**Octanoic acid, 2,3-dihydroxypropyl ester**<br>**9-octadecenamide**<br>**phthalic acid, di-(1-hexen-5-yl) ester**<br>**emylcamate**<br>6-methyl octadecane<br>dodecanoic acid, 3-hydroxy<br>17-methyl octadecanoic acid<br>4-tetradecanol<br>tridecanoic acid, 4,8,12-trimethyl-	unknown<br>unknown<br>unknown<br>**p-cresol, 2,2'methylenebis[6-tert-butyl-**<br>**palmitic acid beta monoglyceride**<br>**alpha monostearin (stearic acid alpha monoglyceride)**<br>**Oleamide (oleic acid amide)**<br>**plastic contamination**<br>**(3-pentanol, 3-methyl-, carbamate)**<br>hydrocarbon<br>unknown<br>unknown<br>unknown<br>unknown
GS53	sediment	not measured	-
GS53	unground surface	not measured	-
GS56	ground surface 1 of 2	17-methyl octadecanoic acid<br>15-methyl-hexadecanoic acid<br>n-pentadecanol<br>15-methyl hexadecanoic acid<br>16-methyl heptadecanoic acid<br>9-octadecenal<br>Phenol, 2,2'-methylenebis[6-(1,1-dimethylethyl)-4-methyl-	unknown<br>unknown<br>unknown<br>unknown<br>unknown<br>unknown<br>unknown
GS56	ground surface 2 of 2	**homoserine**<br>4-penten-1-ol, 3-methyl-<br>1-undecanol<br>1-hexacosanol<br>17-methyl octadecanoic acid	**Butyric acid, 2-amino-4-hydroxy-**<br>unknown<br>unknown<br>unknown<br>unknown
GS56	sediment	not measured	-
GS56	unground surface	not measured	-
GS73	ground surface 1 of 1	4,4-dimethoxy-2-methyl-2-butanol<br>nonanoic acid<br>diethyltoluamide<br>methylcyclodecane<br>dodecanoic acid<br>n-pentadecanol<br>hexadecanois acid<br>**ascorbic acid, 2,6-dihexadecanoate**<br>3,7,11-trimethyl-1-dodecanol<br>3-hydroxy-dodecanoic acid<br>16-methyl heptadecanoic acid<br>**octadecanoic acid, 2-hydroxy-1,3-propanediyl ester**<br>8-octadecenal<br>10-methyl-nonadecane<br>6-methyl-octadecane<br>**butyrolactone**<br>1-heptyn-4-ol	unknown<br>unknown<br>unknown<br>unknown<br>unknown<br>unknown<br>unknown<br>**vitamin C fatty ester**<br>unknown<br>unknown<br>unknown<br>**octadecanoic acid diglyceride (glycerin-1,3-distearate)**<br>unknown<br>unknown<br>unknown<br>**2(3H)-furanone, dihydro-**<br>unknown

Artefact ID	Sample location	Detected compound(s)	Origin/Library match(es) + Reference(s)
		tridecanoic acid<br>1-methyl inosine<br>1-decanol<br>15-methyl-hexadecanoic acid<br>**16-methyl-heptadecanoic acid**	unknown<br>unknown<br>unknown<br>unknown<br>**(octadecanoic acid)**
GS73	sediment	not measured	-
GS73	unground surface	not measured	-
GS74	ground surface 1 of 1	**hexadecanoic acid**<br><br><br><br><br>**octadecanoic acid**<br><br><br>caprolactam<br>2-decanol<br>diethyltoluamide<br>hexanoic acid, 3,5,5-trimethyl-, 2-ethylhexyl ester<br>farnesane<br>2-methyloctacosane<br>**hexadecanoic acid, 2-hydroxy-1-(hydroxymethyl)ethyl ester**<br>**octadecanoic acid, 2,3-dihydroxypropyll ester**	**degraded fatty acid, present in plants, animals + beeswax; may indicate handling contamination:** Gutiérrez et al. 1999; Malainey et al. 1999; Regert et al. 2001; Croxton et al. 2010; Malarvizhi & Ramakrishnan 2011; Al-Shammari et al. 2012; Lakshmi prava et al. 2012; Abozid et al. 2013; Michalski et al. 2013; Maia & Nunes 2013<br>**fatty acid, present in plants, animals + beeswax; may indicate handling contamination:** Gutiérrez et al. 1999; Malainey et al. 1999; Regert et al. 2001; Croxton et al. 2010; Abirami & Rajendran 2011; Michalski et al. 2013<br>unknown<br>unknown<br>unknown<br>unknown<br>unknown<br>unknown<br>**2-mono-palmitin (palmitic acid beta monoglyceride)**<br>**1-mono-stearin (stearic acid, alpha monoglyceride)**
GS74	sediment	not measured	-
GS74	unground surface	not measured	-
GS75	ground surface 1 of 2 (from central depression)	**oxirane, 2-methyl-2-phenyl-**<br>phenol, 3,5-bis(1,1-dimethylethyl)-<br>**hexadecanoic acid**<br><br><br><br>**ascorbic acid, 2,6-dihexadecanoate**<br>**octadecanoic acid**<br><br><br>hexadecanoic acid, 1-(hydroxymethyl)-1,2-ethanediyl ester<br>**n-pentadecanol**<br>phenol, 2,2'-methylenebis[6-(1,1-dimethylethyl)-4-methyl-<br>monopalmitin<br>**hexadecanoic acid, 2-hydroxy-1-(hydroxymethyl)ethyl ester**<br>**octadecanoic acid, 2,3-dihydroxypropyl ester**	**alpha.beta.epoxy-Cumene**<br>unknown<br>**degraded fatty acid, present in plants, animals + beeswax; may indicate handling contamination:** Gutiérrez et al. 1999; Malainey et al. 1999; Regert et al. 2001; Croxton et al. 2010; Malarvizhi & Ramakrishnan 2011; Al-Shammari et al. 2012; Lakshmi prava et al. 2012; Abozid et al. 2013; Michalski et al. 2013; Maia & Nunes 2013<br>**vitamin C fatty ester**<br>**fatty acid, present in plants, animals + beeswax; may indicate handling contamination:** Gutiérrez et al. 1999; Malainey et al. 1999; Regert et al. 2001; Croxton et al. 2010; Abirami & Rajendran 2011; Michalski et al. 2013 **dipalmitin glycerol**<br>unknown<br>**p-cresol, 2,2'-methylenebis[6-tert-butyl-**<br>unknown<br>**2-mono-palmitin (palmitic acid beta monoglyceride)**<br>**1-mono-stearin (Stearic acid alpha monoglyceride)**
GS75	ground surface 2 of 2	1-decanol<br>17-methyl-octadecanoic acid<br>**hexadecanoic acid**	unknown<br>unknown<br>**degraded fatty acid, present in plants, animals + beeswax; may indicate handling contamination:** Gutiérrez et al. 1999; Malainey et al. 1999; Regert et al. 2001; Croxton et al. 2010; Malarvizhi & Ramakrishnan 2011; Al-Shammari et al. 2012; Lakshmi prava et al. 2012; Abozid et al. 2013; Michalski et al. 2013; Maia & Nunes 2013

Artefact ID	Sample location	Detected compound(s)	Origin/Library match(es) + Reference(s)
		**octadecanoic acid**	**fatty acid, present in plants, animals + beeswax; may indicate handling contamination:** Gutiérrez et al. 1999; Malainey et al. 1999; Regert et al. 2001; Croxton et al. 2010; Abirami & Rajendran 2011; Michalski et al. 2013
GS75	sediment	not measured	-
GS75	unground surface	not measured	-
GS79	ground surface 1 of 1	no compounds detected	-
GS79	sediment	not measured	-
GS79	unground surface	not measured	-
GS80	ground surface 1 of 1	not measured	-
GS80	sediment	not measured	-
GS80	unground surface	not measured	-
GS82	ground surface 1 of 2	not measured	-
GS82	ground surface 2 of 2	not measured	-
GS82	sediment	not measured	-
GS82	unground surface	not measured	-
GS86	ground surface 1 of 1	not measured	-
GS86	sediment	not measured	-
GS86	unground surface	not measured	-
GS87	ground surface 1 of 1	not measured	-
GS87	sediment	not measured	-
GS87	unground surface	not measured	-
UPGS1	ground surface 1 of 3	no compounds detected	-
UPGS1	ground surface 2 of 3	no compounds detected	-
UPGS1	ground surface 3 of 3	no compounds detected	-
UPGS1	sediment	**azelaic acid**	**degraded fatty acid, present in acne creams, plants + animals:** Nicolet & Liddle 1916; Eerkins 2002; Garelnabi et al. 2010; Al-Shammari et al. 2012
UPGS1	unground surface	not measured	-
UPGS2	ground surface 1 of 1	no compounds detected	-
UPGS2	sediment	not measured	-
UPGS2	unground surface	not measured	-
UPGS3	ground surface 1 of 1	**azelaic acid**	**degraded fatty acid, present in acne creams, plants + animals:** Nicolet & Liddle 1916; Eerkins 2002; Garelnabi et al. 2010; Al-Shammari et al. 2012
UPGS3	sediment	not measured	-
UPGS3	unground surface	not measured	-
UPGS4	ground surface 1 of 1	**azelaic acid**	**degraded fatty acid, present in acne creams, plants + animals:** Nicolet & Liddle 1916; Eerkins 2002; Garelnabi et al. 2010; Al-Shammari et al. 2012
UPGS4	sediment	no compounds detected	-
UPGS4	unground surface	not measured	-
UPGS5	ground surface 1 of 1	**azelaic acid**	**degraded fatty acid, present in acne creams, plants + animals:** Nicolet & Liddle 1916; Eerkins 2002; Garelnabi et al. 2010; Al-Shammari et al. 2012
UPGS5	sediment	not measured	-
UPGS5	unground surface	not measured	-
UPGS6	ground surface 1 of 1	scopoletin Morinda, Solanum<br>**3,4-dihydroisoquinoline, 1-(3-hydroxybenzyl)-6-methoxy-**<br>azelaic acid<br>3,5-dimethoxy-4-hydroxybenzoate	unknown<br>**alkaloid (bioactive), present in toxic plants**<br>unknown<br>unknown

Artefact ID	Sample location	Detected compound(s)	Origin/Library match(es) + Reference(s)
		5-allyl-1-methoxy-2,3-dihydroxybenzene	unknown
		benzhydrazide, 4-(4-(4-methoxyphenyl)-5-methylthiazol-2-ylamino)-	unknown
		N-(2-hydroxyethyl)-9-methyl-4-methylthio-1,2-carbazoledicarboximide	unknown
		**3,5-dimethoxy-4-hydroxy-cinnamaldehyde**	**sinapaldehyde, present in plants (bioactive):** Gopalakrishnan & Vadivel 2011; Cabrita et al. 2012; Heigenmoser et al. 2013
		**hexadecanoic acid**	**degraded fatty acid, present in plants, animals + beeswax; may indicate handling contamination:** Gutiérrez et al. 1999; Malainey et al. 1999; Regert et al. 2001; Croxton et al. 2010; Malarvizhi & Ramakrishnan 2011; Al-Shammari et al. 2012; Lakshmi prava et al. 2012; Abozid et al. 2013; Michalski et al. 2013; Maia & Nunes 2013
		benzhydrazide, 4-(4-(4-methoxyphenyl)-5-methylthiazol-2-ylamino)-	unknown
		2,4-imidazolidinedione, 5-(3,4-dihydroxy-phenyl)-3-methyl-5-phenyl-	unknown
		2,4-imidazolidinedione, 5-(3,4-dihydroxy-phenyl)-3-methyl-5-phenyl-	unknown
		**octadecanoic acid**	**fatty acid, present in plants, animals + beeswax; may indicate handling contamination:** Gutiérrez et al. 1999; Malainey et al. 1999; Regert et al. 2001; Croxton et al. 2010; Abirami & Rajendran 2011; Michalski et al. 2013
		**11-cis-octdecenoic acid**	**present in animals, plants (including roots, seeds + nuts e.g., *Asclepias* and *Macadamia*); bacteria:** Chisholm & Hopkins 1960; Holloway & Wakil 1964; Shibahara et al. 1986; Miyatani et al. 2001; Sağlik et al. 2002; Denev et al. 2011, Kumar et al. 2014; Ugoeze.et al. 2014.
UPGS6	sediment	**azelaic acid**	**degraded fatty acid, present in acne creams, plants + animals:** Nicolet & Liddle 1916; Eerkins 2002; Garelnabi et al. 2010; Al-Shammari et al. 2012
UPGS6	unground surface	not measured	-
UPGS7	ground surface 1 of 1	no compounds detected	-
UPGS7	sediment	no compounds detected	-
UPGS7	unground surface	not measured	-
UPGS9	ground surface 1 of 1	**azelaic acid**	**degraded fatty acid, present in acne creams, plants + animals:** Nicolet & Liddle 1916; Eerkins 2002; Garelnabi et al. 2010; Al-Shammari et al. 2012
UPGS9	sediment	not measured	-
UPGS9	unground surface	not measured	-
UPGS10	ground surface 1 of 1	no compounds detected	-
UPGS10	sediment	**tridecan-2-ol**	**present in bacteria; plants; insects:** Bruschini et al. 2006, Chikhi et al. 2012, Weise et al. 2012
UPGS10	unground surface	not measured	-
UPGS11	ground surface 1 of 1	**2-hydroxypropanoic acid**	**lactic acid, present in plants + animals**
		**2-ethylhexanoic acid**	**present in plants, honey:** Fiehn et al. 2000, Jerković & Marijanović 2010, Hammami et al. 2011, Wang et al. 2012
		**benzoic acid**	**present in plants (antibacterial), e.g., *Petalostigma, Ficus:*** Fountain et al. 1995; Cock & Kalt 2012; Kalt & Cock 2014; Jeong et al. 2014; Saravanan et al. 2014
		phosphate	unknown
		2,4-imidazolidinedione, 5-(3,4-dihydroxyphenyl)-3-methyl-5-phenyl-	unknown
		prosta-5,13-dien-1-oic acid, 9,11,15-trihydroxy-, (5Z,9.alpha.,11.alpha.,13E, 15S)-	unknown
		**levoglucosan**	**present in burnt organics:** Kehrwald et al. 2012, Latif et al. 2012
		benz(a)anthracene-7-carbonitrile	unknown
		**monolinoleoylglycerol**	**present in plants:** Bhuiyan et al. 2009; Merlin et al. 2009; Lakshmi prava & RajaLakshmi prava 2011; Malarvizhi & Ramakrishnan 2011; Murugesan & Panneerselvam 2013; Sheela & Uthayakumari 2013
		**phthalic acid, butyl decyl ester**	**present in plants:** Ranganathan 2014

Artefact ID	Sample location	Detected compound(s)	Origin/Library match(es) + Reference(s)
		**hexadecanoic acid**	**degraded fatty acid, present in plants, animals + beeswax; may indicate handling contamination:** Gutiérrez et al. 1999; Malainey et al. 1999; Regert et al. 2001; Croxton et al. 2010; Malarvizhi & Ramakrishnan 2011; Al-Shammari et al. 2012; Lakshmi prava et al. 2012; Abozid et al. 2013; Michalski et al. 2013; Maia & Nunes 2013
		octadecanol	unknown
		**hexadecanoic acid 1,1-dimethylethyl ester**	**present in plants:** Prakash et al. 2011
		**octadecanoic acid**	**fatty acid, present in plants, animals + beeswax; may indicate handling contamination:** Gutiérrez et al. 1999; Malainey et al. 1999; Regert et al. 2001; Croxton et al. 2010; Abirami & Rajendran 2011; Michalski et al. 2013
		**octadecanoic acid butyl ester**	**present in plants:** Nayak et al. 2014
		phthalic acid, isodecyl octyl ester	unknown
		monolinoleoylglycerol	unknown
UPGS11	sediment	no compounds detected	-
UPGS11	unground surface	not measured	-
UPGS12	ground surface 1 of 1	no compounds detected	-
UPGS12	sediment	no compounds detected	-
UPGS12	unground surface	not measured	-
UPGS14	ground surface 1 of 1	sulphur	unknown
		propylbenzene	unknown
		1-ethyl-2-methyl-benzene	unknown
		**isopropylbenzene**	**cumene**
		**heptanoic acid**	**enanthic acid, present in essential oils**
		glyoxylic acid	unknown
		**azelaic acid**	**degraded fatty acid, present in acne creams, plants + animals:** Nicolet & Liddle 1916; Eerkins 2002; Garelnabi et al. 2010; Al-Shammari et al. 2012
		**hexadecanoic acid**	**degraded fatty acid, present in plants, animals + beeswax; may indicate handling contamination:** Gutiérrez et al. 1999; Malainey et al. 1999; Regert et al. 2001; Croxton et al. 2010; Malarvizhi & Ramakrishnan 2011; Al-Shammari et al. 2012; Lakshmi prava et al. 2012; Abozid et al. 2013; Michalski et al. 2013; Maia & Nunes 2013
		**octadecanoic acid**	**fatty acid, present in plants, animals + beeswax; may indicate handling contamination:** Gutiérrez et al. 1999; Malainey et al. 1999; Regert et al. 2001; Croxton et al. 2010; Abirami & Rajendran 2011; Michalski et al. 2013
UPGS14	sediment	no compounds detected	-
UPGS14	unground surface	not measured	-
UPGS15	ground surface 1 of 1	no compounds detected	-
UPGS15	sediment	no compounds detected	-
UPGS15	unground surface	not measured	-
UPGS16	ground surface 1 of 3	no compounds detected	-
UPGS16	ground surface 2 of 3	propylene glycol	unknown
		**azelaic acid**	**degraded fatty acid, present in acne creams, plants + animals:** Nicolet & Liddle 1916; Eerkins 2002; Garelnabi et al. 2010; Al-Shammari et al. 2012
UPGS16	ground surface 3 of 3	2,3-dihydroxybutane	unknown
UPGS16	sediment	no compounds detected	-
UPGS16	unground surface	not measured	-
UPGS17	ground surface 1 of 2	**azelaic acid**	**degraded fatty acid, present in acne creams, plants + animals:** Nicolet & Liddle 1916; Eerkins 2002; Garelnabi et al. 2010; Al-Shammari et al. 2012
		3-phenyl-prop-2-ene	unknown

Artefact ID	Sample location	Detected compound(s)	Origin/Library match(es) + Reference(s)
UPGS17	ground surface 2 of 2	no compounds detected	-
UPGS17	sediment	not measured	-
UPGS17	unground surface	not measured	-
UPGS18	ground surface 1 of 1	1-ethyl-3-methyl-benzene<br>1-ethyl-4-methy-benzene<br>oxanilic acid	unknown<br>unknown<br>unknown
UPGS18	sediment	no compounds detected	-
UPGS18	unground surface	not measured	-
UPGS19	ground surface 1 of 1	not measured	-
UPGS19	sediment	no compounds detected	-
UPGS19	unground surface	not measured	-
UPGS21	ground surface 1 of 4	**monoamidoethylmalonic acid**	**present in plants:** Zhang et al. 2010
UPGS21	ground surface 2 of 4	**1-ethyl-4-methylbenzene**<br><br>1-ethyl-2-methylbenzene<br>**3-methyl-2-hydroxy-2-butenoic acid**<br>**N-(2-methyl-1-oxo-2-propenyl)-N-glycine**<br>**hexadecanoic acid**	**present in plants, including seeds and/or nuts; plant *Vitex:*** de Lacy Costello et al. 2001; Ciganek et al. 2007; Janakiraman et al. 2012; Omikorede et al. 2012.<br>unknown<br>**2-Hydroxy-3-methylbutyric acid, present in plants**<br>**amino acid derivative, unknown origin**<br>**degraded fatty acid, present in plants, animals + beeswax; may indicate handling contamination:** Gutiérrez et al. 1999; Malainey et al. 1999; Regert et al. 2001; Croxton et al. 2010; Malarvizhi & Ramakrishnan 2011; Al-Shammari et al. 2012; Lakshmi prava et al. 2012; Abozid et al. 2013; Michalski et al. 2013; Maia & Nunes 2013
UPGS21	ground surface 3 of 4	not measured	-
UPGS21	ground surface 4 of 4	not measured	-
UPGS21	sediment	no compounds detected	-
UPGS21	unground surface	not measured	-
UPGS22	ground surface 1 of 2	no compounds detected	-
UPGS22	ground surface 2 of 2	**1-ethyl-4-methylbenzene**<br><br>**Cyclopentanol**<br><br>oxanilic acid<br>4,6-dimethyl-2-thioxo-1,2-dihydro-3-pyridinecarbonitrile<br>**hexadecanoic acid**	**present in plants, including seeds and/or nuts; plant *Vitex:*** de Lacy Costello et al. 2001; Ciganek et al. 2007; Janakiraman et al. 2012; Omikorede et al. 2012.<br>**present in plants:** Hadi et al. 2013, Helen et al. 2011, Jerkovic & Mastelic. 2001, Ramesh et al. 2014<br>unknown<br>unknown<br>**degraded fatty acid, present in plants, animals + beeswax; may indicate handling contamination:** Gutiérrez et al. 1999; Malainey et al. 1999; Regert et al. 2001; Croxton et al. 2010; Malarvizhi & Ramakrishnan 2011; Al-Shammari et al. 2012; Lakshmi prava et al. 2012; Abozid et al. 2013; Michalski et al. 2013; Maia & Nunes 2013
UPGS22	sediment	no compounds detected	-
UPGS22	unground surface	not measured	-
UPGS23	ground surface 1 of 1	**2-hydroxypropanoic acid**<br>**hexadecanoic acid**<br><br><br><br>**octadecanoic acid**	**lactic acid, present in plants + animals**<br>**degraded fatty acid, present in plants, animals + beeswax; may indicate handling contamination:** Gutiérrez et al. 1999; Malainey et al. 1999; Regert et al. 2001; Croxton et al. 2010; Malarvizhi & Ramakrishnan 2011; Al-Shammari et al. 2012; Lakshmi prava et al. 2012; Abozid et al. 2013; Michalski et al. 2013; Maia & Nunes 2013<br>**fatty acid, present in plants, animals + beeswax; may indicate handling contamination:** Gutiérrez et al. 1999; Malainey et al. 1999; Regert et al. 2001; Croxton et al. 2010; Abirami & Rajendran 2011; Michalski et al. 2013

Artefact ID	Sample location	Detected compound(s)	Origin/Library match(es) + Reference(s)
UPGS23	sediment	no compounds detected	-
UPGS23	unground surface	not measured	-
UPGS24	ground surface 1 of 1	1-ethyl-2-methyl-benzene<br>oxanilic acid<br>**N-(2-methyl-1-oxo-2-propenyl)-N-glycine**	unknown<br>unknown<br>**amino acid derivative, unknown origin**
UPGS24	sediment	no compounds detected	-
UPGS24	unground surface	not measured	-
UPGS25	ground surface 1 of 1	**2-hydroxypropanoic acid**<br>phosphate<br>**monolinoleoylglycerol**<br><br><br>**azelaic acid**<br><br>**hexadecanoic acid**<br><br><br><br>**octadecanoic acid**	**lactic acid, present in plants + animals**<br>unknown<br>**present in plants:** Bhuiyan et al. 2009; Merlin et al. 2009; Lakshmi prava & RajaLakshmi prava 2011; Malarvizhi & Ramakrishnan 2011; Murugesan & Panneerselvam 2013; Sheela & Uthayakumari 2013<br>**degraded fatty acid, present in acne creams, plants + animals:** Nicolet & Liddle 1916; Eerkins 2002; Garelnabi et al. 2010; Al-Shammari et al. 2012<br>**degraded fatty acid, present in plants, animals + beeswax; may indicate handling contamination:** Gutiérrez et al. 1999; Malainey et al. 1999; Regert et al. 2001; Croxton et al. 2010; Malarvizhi & Ramakrishnan 2011; Al-Shammari et al. 2012; Lakshmi prava et al. 2012; Abozid et al. 2013; Michalski et al. 2013; Maia & Nunes 2013<br>**fatty acid, present in plants, animals + beeswax; may indicate handling contamination:** Gutiérrez et al. 1999; Malainey et al. 1999; Regert et al. 2001; Croxton et al. 2010; Abirami & Rajendran 2011; Michalski et al. 2013
UPGS25	sediment	no compounds detected	-
UPGS25	unground surface	not measured	-
UPGS26	ground surface 1 of 1	**ethanediol**<br>2-hydroxypropanol	**ethylene glycol, present in household cleaning products; paints; cosmetics**<br>unknown
UPGS26	sediment	**9,12,15-octadecatrienoic acid glycerol**	**linoleic acid, present in propolis; plants including seeds; nuts (e.g., *Macadamia* sp.); animals:** Malainey et al. 1999; Abirami & Rajendran 2011; Krishna et al. 2012; Abozid et al. 2013; Choudhari & Kareppa 2013; Chaudhary et al. 2014; Ertas et al. 2014; Sáez et al. 2014
UPGS26	unground surface	not measured	-
UPGS27	ground surface 1 of 1	no compounds detected	-
UPGS27	sediment	no compounds detected	-
UPGS27	unground surface	not measured	-
UPGS28	ground surface 1 of 1	no compounds detected	-
UPGS28	sediment	no compounds detected	-
UPGS28	unground surface	not measured	-
UPGS29	ground surface 1 of 1	**2-hydroxypropanoic acid**<br>**nonanoic acid**<br>**hexadecanoic acid**<br><br><br><br>**monolinoleoylglycerol**<br><br><br>2-amino-1-(4-methoxyphenyl)-5-phenyl-1H-pyrrole-3,4-dicarbonitrile<br>**octadecanoic acid**	**lactic acid, present in plants + animals**<br>**present in plants:** Knudsen et al. 1993<br>**degraded fatty acid, present in plants, animals + beeswax; may indicate handling contamination:** Gutiérrez et al. 1999; Malainey et al. 1999; Regert et al. 2001; Croxton et al. 2010; Malarvizhi & Ramakrishnan 2011; Al-Shammari et al. 2012; Lakshmi prava et al. 2012; Abozid et al. 2013; Michalski et al. 2013; Maia & Nunes 2013<br>**present in plants:** Bhuiyan et al. 2009; Merlin et al. 2009; Lakshmi prava & RajaLakshmi prava 2011; Malarvizhi & Ramakrishnan 2011; Murugesan & Panneerselvam 2013; Sheela & Uthayakumari 2013<br>unknown

Artefact ID	Sample location	Detected compound(s)	Origin/Library match(es) + Reference(s)
			**fatty acid, present in plants, animals + beeswax; may indicate handling contamination:** Gutiérrez et al. 1999; Malainey et al. 1999; Regert et al. 2001; Croxton et al. 2010; Abirami & Rajendran 2011; Michalski et al. 2013
UPGS29	sediment	not measured	-
UPGS29	unground surface	not measured	-
UPGS30	ground surface 1 of 1	1-(3-methylbutyl)-2,3,5-trimethylbenzene<br>1-(3-methylbutyl)-2,3,5-trimethylbenzene	unknown<br>unknown
UPGS30	unground surface	not measured	-
UPGS30	sediment	not measured	-
UPGS31	ground surface 1 of 1	no compounds detected	-
UPGS31	sediment	not measured	-
UPGS31	unground surface	not measured	-
UPGS32	ground surface 1 of 1	**2-hydroxypropanoic acid**<br>**2,4-dintrophenyl-arginine**<br>**hexadecanoic acid**<br>**octadecanoic acid**	**lactic acid, present in plants + animals**<br>**amino acid derivative, unkown origin**<br>**degraded fatty acid, present in plants, animals + beeswax; may indicate handling contamination:** Gutiérrez et al. 1999; Malainey et al. 1999; Regert et al. 2001; Croxton et al. 2010; Malarvizhi & Ramakrishnan 2011; Al-Shammari et al. 2012; Lakshmi prava et al. 2012; Abozid et al. 2013; Michalski et al. 2013; Maia & Nunes 2013<br>**fatty acid, present in plants, animals + beeswax; may indicate handling contamination:** Gutiérrez et al. 1999; Malainey et al. 1999; Regert et al. 2001; Croxton et al. 2010; Abirami & Rajendran 2011; Michalski et al. 2013
UPGS32	sediment	not measured	-
UPGS32	unground surface	not measured	-
UPGS33	ground surface 1 of 1	no compounds detected	-
UPGS33	sediment	not measured	-
UPGS33	unground surface	not measured	-
UPGS34	ground surface 1 of 1	**2-hydroxypropanoic acid**<br>**hexadecanoic acid**<br>androst-2-en-17-amine, 4,4-dimethyl-N-(2-phenylethyl)-, (5.alpha.)-<br>**octadecanoic acid**	**lactic acid, present in plants + animals**<br>**degraded fatty acid, present in plants, animals + beeswax; may indicate handling contamination:** Gutiérrez et al. 1999; Malainey et al. 1999; Regert et al. 2001; Croxton et al. 2010; Malarvizhi & Ramakrishnan 2011; Al-Shammari et al. 2012; Lakshmi prava et al. 2012; Abozid et al. 2013; Michalski et al. 2013; Maia & Nunes 2013<br>unknown<br>**fatty acid, present in plants, animals + beeswax; may indicate handling contamination:** Gutiérrez et al. 1999; Malainey et al. 1999; Regert et al. 2001; Croxton et al. 2010; Abirami & Rajendran 2011; Michalski et al. 2013
UPGS34	sediment	not measured	-
UPGS34	unground surface	not measured	-
UPGS35	ground surface 1 of 1	no compounds detected	-
UPGS35	sediment	not measured	-
UPGS35	unground surface	not measured	-
UPGS36	ground surface 1 of 1	no compounds detected	-
UPGS36	sediment	not measured	-
UPGS36	unground surface	not measured	-
UPGS37	ground surface 1 of 1	no compounds detected	-
UPGS37	sediment	no compounds detected	-
UPGS37	unground surface	not measured	-

Artefact ID	Sample location	Detected compound(s)	Origin/Library match(es) + Reference(s)
UPGS38	ground surface 1 of 1	**azelaic acid**	**degraded fatty acid, present in acne creams, plants + animals:** Nicolet & Liddle 1916; Eerkins 2002; Garelnabi et al. 2010; Al-Shammari et al. 2012
UPGS38	sediment	no compounds detected	-
UPGS38	unground surface	not measured	-
UPGS39	ground surface 1 of 5	no compounds detected	-
UPGS39	ground surface 2 of 5	**ethanediol**<br>**3-phenyl-2-propenol**	**ethylene glycol, present in household cleaning products; paints; cosmetics**<br>**cinnamyl alcohol, present in plants:** de Vega et al. 2013, Wang et al. 2013
UPGS39	ground surface 3 of 5	no compounds detected	-
UPGS39	ground surface 4 of 5	not measured	-
UPGS39	ground surface 5 of 5	no compounds detected	-
UPGS39	sediment	no compounds detected	-
UPGS39	unground surface	not measured	-
L49	ground surface 1 of 1	lactic acid<br>oxanilic acid<br>**2-ethylhexanoic acid**	unknown<br>unknown<br>**present in plant, honey:** Fiehn et al. 2000, Jerković & Marijanović 2010, Hammami et al. 2011, Wang et al. 2012
L49	sediment	no compounds detected	-
L49	unground surface	not measured	-
L52	ground surface 1 of 2	4,6-dimethyl-2-thioxo-1,2-dihydro-3-pyridinecarbonitrile<br>**heptanoic acid**	unknown<br>**enanthic acid, present in essential oils**
L52	ground surface 2 of 2	no compounds detected	-
L52	sediment	no compounds detected	-
L52	unground surface	not measured	-
L813	ground surface 1 of 1	phosphate<br>**azelaic acid**	unknown<br>**degraded fatty acid, present in acne creams, plants + animals:** Nicolet & Liddle 1916; Eerkins 2002; Garelnabi et al. 2010; Al-Shammari et al. 2012
L813	sediment	no compounds detected	-
L813	unground surface	not measured	-
L868	ground surface 1 of 1	Bis(4-hydroxyphenyl)methane<br>**monoamidoethylmalonic acid**<br>**tridecan-2-ol**<br>**monolinoleoylglycerol**<br>**hexadecanoic acid**<br>15-isopropenyl-oxacyclopentadecan-2-one<br>**octadecanoic acid**	unknown<br>**present in plants:** Zhang et al. 2010<br>**present in bacteria; plants; insects:** Bruschini et al. 2006, Chikhi et al. 2012, Weise et al. 2012<br>**present in plants:** Bhuiyan et al. 2009; Merlin et al. 2009; Lakshmi prava & RajaLakshmi prava 2011; Malarvizhi & Ramakrishnan 2011; Murugesan & Panneerselvam 2013; Sheela & Uthayakumari 2013<br>**degraded fatty acid, present in plants, animals + beeswax; may indicate handling contamination:** Gutiérrez et al. 1999; Malainey et al. 1999; Regert et al. 2001; Croxton et al. 2010; Malarvizhi & Ramakrishnan 2011; Al-Shammari et al. 2012; Lakshmi prava et al. 2012; Abozid et al. 2013; Michalski et al. 2013; Maia & Nunes 2013<br>unknown<br>**fatty acid, present in plants, animals + beeswax; may indicate handling contamination:** Gutiérrez et al. 1999; Malainey et al. 1999; Regert et al. 2001; Croxton et al. 2010; Abirami & Rajendran 2011; Michalski et al. 2013
L868	sediment	no compounds detected	-
L868	unground surface	not measured	-
L894	ground surface 1 of 1	no compounds detected	-

Artefact ID	Sample location	Detected compound(s)	Origin/Library match(es) + Reference(s)
L894	sediment	no compounds detected	-
L894	unground surface	not measured	-
L1349	ground surface 1 of 1	**1-ethyl-4-methylbenzene**<br><br>1-ethyl-2-methylbenzene<br>**2-hydroxypropanoic acid**<br>oxanilic acid<br>4,6-dimethyl-2-thioxo-1,2-dihydro-3-pyridinecarbonitrile<br>**monoamidoethylmalonic acid**<br>**hexadecanoic acid**	**present in plants, including seeds and/or nuts; plant *Vitex*:** de Lacy Costello et al. 2001; Ciganek et al. 2007; Janakiraman et al. 2012; Omikorede et al. 2012<br>unknown<br>**lactic acid, present in plants + animals**<br>unknown<br>unknown<br>**present in plants:** Zhang et al. 2010<br>**degraded fatty acid, present in plants, animals + beeswax; may indicate handling contamination:** Gutiérrez et al. 1999; Malainey et al. 1999; Regert et al. 2001; Croxton et al. 2010; Malarvizhi & Ramakrishnan 2011; Al-Shammari et al. 2012; Lakshmi prava et al. 2012; Abozid et al. 2013; Michalski et al. 2013; Maia & Nunes 2013
L1349	sediment	no compounds detected	-
L1349	unground surface	not measured	-
R2	ground surface 1 of 1	**azelaic acid**	**degraded fatty acid, present in acne creams, plants + animals:** Nicolet & Liddle 1916; Eerkins 2002; Garelnabi et al. 2010; Al-Shammari et al. 2012
R2	sediment	no compounds detected	-
R2	unground surface	not measured	-
R5	ground surface 1 of 3	no compounds detected	-
R5	ground surface 2 of 3	**1-ethyl-4-methylbenzene**<br><br>**2-hydroxypropanoic acid**<br>**heptanoic acid**<br>**hexadecanoic acid**	**present in plants, including seeds and/or nuts; plant *Vitex*:** de Lacy Costello et al. 2001; Ciganek et al. 2007; Janakiraman et al. 2012; Omikorede et al. 2012<br>**lactic acid, present in plants + animals**<br>**enanthic acid, present in essential oils**<br>**degraded fatty acid, present in plants, animals + beeswax; may indicate handling contamination:** Gutiérrez et al. 1999; Malainey et al. 1999; Regert et al. 2001; Croxton et al. 2010; Malarvizhi & Ramakrishnan 2011; Al-Shammari et al. 2012; Lakshmi prava et al. 2012; Abozid et al. 2013; Michalski et al. 2013; Maia & Nunes 2013
R5	ground surface 3 of 3	no compounds detected	-
R5	sediment	no compounds detected	-
R5	unground surface	not measured	-
R66	ground surface 1 of 3	no compounds detected	-
R66	ground surface 2 of 3	no compounds detected	-
R66	ground surface 3 of 3	no compounds detected	-
R66	sediment	no compounds detected	-
R66	unground surface	not measured	-
R68	ground surface 1 of 2	**benzoic acid**<br><br>**azelaic acid**<br><br><br>**hexadecanoic acid**	**present in plants (antibacterial), e.g., *Petalostigma, Ficus:*** Fountain et al. 1995; Cock & Kalt 2012; Kalt & Cock 2014; Jeong et al. 2014; Saravanan et al. 2014<br>**degraded fatty acid, present in acne creams, plants + animals:** Nicolet & Liddle 1916; Eerkins 2002; Garelnabi et al. 2010; Al-Shammari et al. 2012<br>**degraded fatty acid, present in plants, animals + beeswax; may indicate handling contamination:** Gutiérrez et al. 1999; Malainey et al. 1999; Regert et al. 2001; Croxton et al. 2010; Malarvizhi & Ramakrishnan 2011; Al-Shammari et al. 2012; Lakshmi prava et al. 2012; Abozid et al. 2013; Michalski et al. 2013; Maia & Nunes 2013
R68	ground surface 2 of 2	**isopropylbenzene**<br>**1-ethyl-4-methylbenzene**	**cumene**<br>**present in plants, including seeds and/or nuts; plant *Vitex*:** de Lacy Costello et al. 2001; Ciganek et al. 2007; Janakiraman et al. 2012; Omikorede et al. 2012

Artefact ID	Sample location	Detected compound(s)	Origin/Library match(es) + Reference(s)
		benzo(b)thiophene-4-acetic acid	unknown
R68	sediment	**azelaic acid**	**degraded fatty acid, present in acne creams, plants + animals:** Nicolet & Liddle 1916; Eerkins 2002; Garelnabi et al. 2010; Al-Shammari et al. 2012
R68	unground surface	not measured	-
R69	ground surface 1 of 1	no compounds detected	-
R69	sediment	**azelaic acid**	**degraded fatty acid, present in acne creams, plants + animals:** Nicolet & Liddle 1916; Eerkins 2002; Garelnabi et al. 2010; Al-Shammari et al. 2012
R69	unground surface	not measured	-

SECTION 9: FUNCTIONAL INTERPRETATIONS

9.1 Introduction

Table S8 lists our functional interpretations of the Madjedbebe grinding stone assemblage, separated by Phase, based on multiple lines of evidence including morphology, usewear and residues. Our combined analyses indicates that the Madjedbebe grinding stones were used for a diverse range of tasks, including the processing of plants (n=60, including seeds, n=17), animal tissue (n=4), red pigments (n=17) and the direct striking/filing of stone (n=5).

Table S8: List of analysed grinding stones and fragments (n=104) by Phase, artefact class and assigned function based on tool stone morphology, usewear and residue traces.

Artefact ID.	Square	Spit	Implement class	Function/material utilised
**Phase 7**	**n=5**			
UPGS39	C2	3	filing stone	metal axe and stone axe sharpening
GS1	D2	5	coupled stone (lower)	plant processing (hard seed)
UPGS2	C2	5	coupled stone (upper)	plant processing (starchy plant)
L49	C2	5	coupled stone (upper)	plant (starchy plant, tubers, USOs, seed)
GS8	D2	8	coupled stone (upper)	plant processing (seed)
**Phase 6**	**n=4**			
R2	C4	4	uncertain	plant processing
L52	C3	5	coupled stone (upper)	plant processing (seed)
GS2	C3	8	coupled stone (upper)	plant processing (hard seed)
R69	C2	18	filing stone	pigment processing
**Phase 5**	**n=15**			
UPGS3	D2	10	uncertain	unknown
UPGS4	D2	16A	filing stone	manufacture-ground
R66	E1	17	coupled stone (lower)	plant processing
R68	E1	18	uncertain	unknown
GS6	C2	21	uncertain/unused	uncertain, possibly unused
UPGS5	B2	21	filing stone	uncertain, plant (hard?)
GS5	D2	21	filing stone	plant processing (wood or fruit)
GS4	E1	21	filing stone; coupled stone	pigment processing; plant processing
GS3	E1	21	filing stone	plant (soft & starchy plant); animal processing
GS7	C2	22A	coupled stone (upper)/hammer	stone, plant processing?
UPGS6	E2	23	uncertain	plant processing
L813	D2	23	filing stone	pigment processing
GS10	D2	24	uncertain	plant processing
GS9	D2	24	uncertain	plant processing; possible animal processing
UPGS7	C2	24A	filing stone	pigment processing
**Phase 4**	**n=33**			
R5	E1	21	coupled stone (lower?)	plant processing (seed)
GS53	B6	24	coupled stone (upper)	plant processing
L868	E1	24	uncertain	unknown
UPGS9	D2	25	coupled stone (upper)	plant processing (hard seed)
UPGS11	D2	25	coupled stone	plant processing
L894	C2	25	uncertain	unknown
GS13	D2	25A	uncertain	plant processing
UPGS10	D2	25A	uncertain	unknown

Artefact ID.	Square	Spit	Implement class	Function/material utilised
GS14	D2	26	coupled stone	plant processing
GS15	D2	26	filing stone	pigment processing
GS16	D2	26	coupled stone (upper)	plant processing (seed)
GS18	D3	26	coupled stone (upper)/hammer	plant processing (seed); hammerstone
UPGS14	E1	26	uncertain	pigment processing; plant processing
UPGS15	C2	26	filing stone	pigment processing
UPGS16	D2	26	coupled stone (upper)	plant processing
UPGS17	C2	26	uncertain	plant processing; possible animal processing
UPGS18	C2	26	filing stone	plant processing (wood)
UPGS19	C2	26	coupled stone (upper)	uncertain, hard material
UPGS21	C2	26	filing stone; coupled stone (upper)	bone working; plant processing (seed)
UPGS22	C2	26	coupled stone	plant processing
GS19	C2	26A	coupled stone	plant processing
UPGS12	D2	26A	uncertain	unknown
GS20	E1	27	coupled stone (lower)	plant processing
UPGS24	E3	28	filing stone	uncertain, possibly plant processing
GS21	E2	28A	filing stone; coupled stone	pigment & plant processing (seed)
GS22	D2	28A	coupled stone (upper)	plant processing
GS23	D2	28A	uncertain	plant processing
UPGS23	D2	28A	filing stone	pigment processing
GS24	E2	28A	coupled stone (upper)	plant processing (seed, nut, roots)
GS26	C2	28A	coupled stone (upper)	plant processing (nuts, seeds)
UPGS37	E2	28A	uncertain	unknown
GS49	C4	29	coupled stone (upper)	uncertain, possible plant processing
GS56*	B6	31	coupled stone (lower)	plant processing
**Phase 3**	**n=16**			
GS27	C1	28	uncertain	unknown
GS28	C1	28	coupled stone (upper)	plant processing
UPGS25	C1	29	filing stone; coupled stone (upper)	pigment processing; plant processing
UPGS28	C2	29A	filing stone	unknown
UPGS29	C2	29A	filing stone	unknown
L1349	D1	32	uncertain	plant processing
GS29	D1	34	coupled stone (lower)	plant processing
GS30	D1	34	coupled stone (lower)	plant processing (seed)
GS31	D1	34	uncertain	uncertain, possible plant processing
GS33	D2	34	filing stone; coupled stone	pigment processing; pos. plant processing
GS35	D1	34	uncertain	uncertain, possible plant processing
UPGS26	C3	35	coupled stone (upper)	plant processing (seed)
UPGS30	C3	36	uncertain	unknown
GS38	C2	37A	filing stone	uncertain, possible axe manufacture
UPGS31	C2	37A	filing stone	unknown
UPGS32	C2	37A	uncertain	unknown
**Phase 2**	**n=29**			
GS36	C1	35	coupled stone	plant processing
GS37	C1	35	coupled stone (upper)	plant processing
UPGS27	C1	36	filing stone	unknown
GS32	C2+C3	37	coupled stone (lower): anvil/mortar	plant processing
GS39	D1	37	coupled stone (lower)	plant (burnt plant, including seed, nut, tuber, wood)
GS40	D2	37	filing stone	pigment processing; plant processing
GS41	D2	38	filing stone	pigment processing
GS43	D1	38	lower stone, filing stone?	plant processing
GS46	D2	39	uncertain	unknown
GS47	D2	39	uncertain	uncertain, possible plant processing

Artefact ID.	Square	Spit	Implement class	Function/material utilised
GS44	D2	39A	uncertain	unknown
GS45	D2	39A	uncertain	unknown
UPGS33	C2	39A	filing stone	unknown
UPGS34	C2	39A	uncertain	uncertain, possible pigment processing
GS48	D2	40A	uncertain	unknown
UPGS35	C3	42	filing stone	uncertain, possible pigment processing
UPGS36	C3	44	filing stone	pigment processing
GS50	C4	45	uncertain	unknown
GS72	B6	50	coupled stone (lower)	unknown
GS73	B5	52	coupled stone (lower); filing stone	plant (seed), possible pigment processing
GS75	B6	52	coupled stone (lower)	plant processing
GS74	C6	53	coupled stone (lower)	plant processing
GS79	B6	54	filing stone	stone axe manufacture
GS80	B6	56	coupled stone (lower)	unknown
GS82	B6	55	coupled stone (lower)	unknown
GS86	B6	63	coupled stone (upper)	unknown
GS87	C6	66	coupled stone (upper)	unknown
R299	B6	50	coupled stone (lower)	uncertain, possible plant processing
R305	B6	51	coupled stone (lower)	unknown
**Unknown**	**n=2**			
UPGS1	-	-	coupled stone (upper)	plant processing
UPGS38	-	-	filing stone	unknown

REFERENCES

Abirami, P. and A. Rajendran 2011 GC-MS determination of bioactive compounds of Indigofera aspalathoides. *Journal of Natural Product and Plant Resources* 1(4): 126–130.

Abozid, M.M. and A.A. El-Kaway Ahmed 2013 Chemical composition of Egyptian and commercial propolis and its effects on liver function and lipid profiles in albino rats. Journal of Biological and Chemical Environmental Research 8(2): 323–340.

Adams, H. and B.J. Camp 1966 The isolation and identification of three alkaloids from Acacia Berlandier. Toxicon 4: 85-89.

Adams, J.L. 1993 Mechanisms of wear on ground stone surfaces. Pacific Coast Archaeological Society Quarterly 29: 61–74.

Adams, J.L. 1994 The development of prehistoric grinding technology in the point pines area, east-central Arizona. Museum Anthropology 19: 17–29.

Adams, J.L. 2002 Ground Stone Analysis: A Technological Approach. University of Utah Press, Salt Lake City.

Alexander, M.P. 1969 Differential staining of aborted and nonaborted pollen. Stain Technology 44: 117–122.

Allen, J. 2017 Yes, Virginia, there is a Santa Claus; He just doesn't bring presents to children who don't believe in him. *Australian Archaeology, 83*(3): 163–165.

Allen, J., and J. O'Connell 2020. A different paradigm for the initial colonisation of Sahul. *Archaeology in Oceania, 55*(1): 1-14.

Alhassanm, A.J., M.S. Sule, H. Abubakar, T.M. Abdulmumin and M.A. Dangambo 2014 Fatty acids composition of Bambara groundnut (Vigna subterranean (L) verd C) grown in Madobi, Kano State - Nigeria. European Scientific Journal 10(24): 143–151.

Al-Mazroa, S.A., L.H. Al-Wahaibi, A.A. Mousa and H.Z. Al-Khathlan 2015 Essential oil of some seasonal flowering plants grown in Saudi Arabia. Arabian Journal of Chemistry 8(2): 212–217.

Aneela, S., A. Dey and S. De 2014 GC-MS analysis of methanolic extract of Prosopis spicigera. International Journal of Phytopharmacology 5(3): 168–171.

Azmat, S., R. Ifzal, M. Rasheed, F. Valimohammad and V. Uddin Ahmad 2010 GC-MS analysis of n-hexane extract from seeds and leaves of Phoenix dactylifers L. Journal of the Chemical Society of Pakistan 32(5): 672–676.

Babu, M., D.P. Raja, A.A. Arockiaraj and J. Vinnarasi 2014 Chemical constituents and their biological activity of their Ulva lactuca linn. International Journal of Pharmaceutics and Drug Analysis 2(7): 595–600.

Bai, S., L. Seasotiya, A. Malik, P. Bharti and S. Dalal 2014 GC-MS analysis of chloroform extract of Acacia nilotica L. leaves. Journal of Pharmacognosy and Phytochemistry 2(6): 79–82.

Banks, W.T. and C.T. Greenwood 1975. Starch and its Components. Edinburgh: Edinburgh University Press.

Barnard, H., Ambrose, S.H., Beehr, D.E., Forster, M.D., Lanehart, R.E., Malainey, M.E., Parr, R.E., Rider, M., Solazzo, C. and Yohe Ii, R.M., 2007. Mixed results of seven methods for organic residue analysis applied to one vessel with the residue of a known foodstuff. Journal of Archaeological Science 34, 28–37.

Bastida, J., S. Berkov, L. Torras, N. Belén Pigni, J.-P. de Andrade, V. Martínez, C. Codina and F. Viladomat 2011 Chemical and biological aspects of Amaryllidaceae alkaloids. In D. Muñoz-Torrero (ed). Recent Advances in Pharmaceutical Sciences, pp. 65–100. Transworld Research Network: Kerala, India.

Bharathy, V. and F. Uthayakumari 2013 Bioactive components in leaves of Jatropha tanjorensis J.L. Ellis and Saroja by GC-MS analysis. International Journal of PharmTech Research 5(4): 1839–1843.

Bhuiyan, M.N.I., J. Begum, P.K. Sardar and M.S. Rahman 2009 Constituents of peel and leaf essential oils of Citrus medica L. Journal of Scientific Research 1(2): 387–392.

Bruschini, C., F. R. Dani, G. Pieraccini, F. Guarna and S. Turillazzi 2006 Volatiles from the venom of five species of paper wasps (Polistes dominulus, P. gallicus, P. nimphus, P. sulcifer and P. olivaceus). Toxicon 47: 812–825.

Buonasera, T. 2007 Investigating the presence of ancient absorbed organic residues in groundstone using GC–MS and other analytical techniques: a residue study of several prehistoric milling tools from central California. Journal of Archaeological Science 34: 1379–1390.

Byrdwell, C. and W.E. Neff 1998 Analysis of hydroxy-containing seed oils using atmospheric pressure chemical ionization mass spectrometry. Journal of Liquid Chromatography and Related Technologies 21(10): 1485–1501.

Cabrita, M.J.B., R. Garcia, N. Martins, M. Gomes Da Silva and A.M.C. Freitas 2012 Gas chromatography in analysis of compounds released from wood into wine. Progress in Agricultural, Biomedical and Industrial Applications 10: 186–208.

Camp, B. and M. J. Norvell 1966 The phenylethylamine alkaloids of native range plants. Economic Botany 20(3): 274–278.

Castrejón, F. M., C. Cruz-Vázquez, M. Fernández-Ruvalcaba, J. Miolina-Torres, J. S. Cruz and M. Ramos Parra 2003 Repellence of Boophilus microplus larvae in Stylosanthes humilis and Stylosanthes hamata plants. Parasitol Latinoam 58: 118–121.

Cecilia, K., K. Glaston, M. Simon and B. Renaud 2012 Volatile organic compounds in brewed Kenyan Arabica coffee genotypes by solid phase extraction gas chromatography mass spectrometry. Food Science and Quality Management 8: 18–26.

Chaloupka, G. and P. Giuliani 1984 Gundulk Abel Gundalg Mayali Flora. Unpublished Report prepared for the Northern Territory Museum of Arts and Sciences.

Chaudhary, N., S. S. Husain and M. Ali 2014 Phytochemical investigation of the stem bark of Ficus hispida L. Journal of Scientific and Innovative Research 3(4): 409–413.

Chen-xing, Z., Z. Mi, H. Jing, D. Ya-fang and L. Bao-cai 2014 Chemical composition and antioxidant activity of the essential oil from the flowers of Artemisia austro-yunnanensis. Journal of Chemical and Pharmaceutical Research 6(7): 1583–1587.

Chikhi, I., H. Allali, M. El Amine Dib, N. Halla, A. Muselli, B. Tabti and J. Costa 2012 Free radical scavenging and antibacterial activity of essential oil and solvent extracts of Iris planifolia (Mill) from Algeria. Journal of Medicinal Plants Research 6(10): 1961–1968.

Chinwe, S.A., C. Anyakora, D. Ota, M. De Waard and H.A.B. Coker 2014 GC-MS analysis, anti-inflammatory and anti-seizure effects of n-octanoic acid from special breed palm kernel nut oil. Planta Medica 80(10): PD118.

Chisholm, M. J., and Hopkins, C. Y. 1960 11-Octadecenoic acid and other fatty acids of Asclepias syriaca seed oil. Canadian Journal of Chemistry 38(6): 805–812.

Chou, K.-S., J.-C. Tsai, and C.-T. Lo 2001 The adsorption of Congo Red and vacuum pump oil by rice hull ash. Bioresource Technology 78(2): 217–219.

Choudhari, S.S. and B.M. Kareppa 2013 Identification of bioactive compounds of Zingiber officinale roscoe rhizomes through gas chromotography and mass spectrometry. International Journal of Pharmaceutical Research and Development 5(8): 16–20.

Ciganek, M., B. Pisarikova and Z. Zraly 2007 Determination of volatile organic compounds in the crude and heat treated amaranth samples. Veterinarni Medicina 52(3): 111–120.

Clarkson, C., Jacobs, Z., Marwick, B., Fullagar, R., Wallis, L., Smith, M., Roberts, R. G., Hayes, E., Lowe, K., Carah, X., and S.A. Florin2017 Human occupation of northern Australia by 65,000 years ago. *Nature*, *547*(7663): 306–310.

Clarkson, C., Roberts, R. G., Jacobs, Z., Marwick, B., Fullagar, R., Arnold, L. J., and Q., Hua 2018 Reply to comments on Clarkson et al. (2017). 'Human occupation of northern Australia by 65,000 years ago'. *Australian Archaeology*, *84*(1): 84–89.

Clement, B.A., C.M. Goff and T.D.A. Forbes 1998 Toxic amines and alkaloides from Acacia rigidula. Phytochemistry 49(5): 1377–1380.

Cock, I.E. and F.R. Kalt 2012 Gas chromatography-mass spectroscopy analysis of a Xanthorrhoea johnsonii leaf extract displaying apparent anaesthetic effects. Journal of Natural Pharmaceuticals 3(2): 78.

Conn, H.J. and R.D. Lillie 196. Biological Stains: A Handbook of the Nature and Uses of the Dyes. Philipelphia: Williams and Wilkins.

Coster, A.C.F. and J.H. Field 2015 What starch grain is that? A geometric morphometric approach to determining plant species origin. Journal of Archaeological Science 58: 9–25.

Coster, A.C.F. and J.H. Field 2018 The shape of things to come—using geometric and morphometric analyses to identify archaeological starch grains. In: Anderssen, R.S., P. Broadbridge, Y. Fukumoto, K. Kajiwara, M. Simpson and I. Turner, I. (Eds.), Agriculture as a Metaphor for Creativity in all Human Endeavors, Mathematics for Industry 28. Springer Nature, Singapore, pp. 1–6.

Croxton, R.S., M.G. Baron, D. Butler, T. Kent, and V.G. Sears 2010 Variation in amino acid and lipid composition of latent fingerprints. Forensic Science International 199: 93–102.

Culvenor, R.A., K.F.M. Reed and S.E. McDonald 2005 Comparative levels of dimethyltryptamine- and tyramine-related alkaloid toxins in Australian cultivars and some wild populations of Phalaris aquatica. Australian Journal of Agricultural Research 56(12): 1395–1403.

Cutler, D.F., T. Botha and D.W. Stevenson 2008 Plant Anatomy: An Applied Approach. Oxford: Blackwell Publishing.

de Andrade, J.P., N. Belén Pigni, L. Torras-Claveria, Y. Guo, S. Berkov, R. Reyes-Chilpa, A. E. Amrani, J.A.S. Zuanazzi, C. Codina, F. Viladomat and J. Bastida 2012 Alkaloids from Hippeastrum genus: chemisty and biological activity. Revista Latinoamericana de Química 40(2): 83–98.

De la Torre, I., A. Benito-Calvo, A. Arroyo, A. Zupancich and T. Proffitt 2013 Experimental protocols for the study of battered stone anvils from Olduvai Gorge (Tanzania). Journal of Archaeological Science 40: 313–332.

De Lacy Costello B.P.J, P. Evans, R.J. Ewen, H.E. Gunson, N.M. Ratcliffe and P.T.N. Spencer-Phillips 1999 Identification of volatiles generated by potato tubers (Solanum tuberosum cv. Maris Piper) infected by Erwinia carotovora, Bacillus polymyxa and Arthrobacter sp. Plant Pathology 48: 345–51

Della Greca, M., A. Fiorentino, A. Izzo, F. Napoli, R. Purcaro and A. Zarrelli 2007 Phytotoxicity of secondary metabolites from Aptenia cordifolia. Chemistry and Biodiversity 4(2): 118-128.

Denev, R.V., I.S. Kuzmanova, S.M. Momchilova and B.M. Nikolova-Damyanova 2011 Resolution and quantification of isomeric fatty acids by silver ion HPLC: fatty acid composition of aniseed oil (Pimpinella anisum, Apiaceae). Journal of AOAC International 94(1): 4–8.

Deshpande, P.S., D.K. Khatri and A.R. Juvekar 2013 GC–MS analysis of phytocomponents from petroleum ether extracted oil of Indigofera cordifolia seeds. Journal of Pharmaceutical Sciences 5: 2831–2838.

Dev, N., A.K. Das, M.A. Hossain, and S.M.M. Rahman 2010 Chemical compositions of different extracts of Ocimum basilicum leaves. Journal of Scientific Research 3(1): 197.

Dunne, J., Evershed, R.P., Salque, M., Cramp, L., Bruni, S., Ryan, K., Biagetti, S. and di Lernia, S. 2012 First dairying in green Saharan Africa in the fifth millennium BC. Nature 486(7403): 390–394.

Eerkens, J. 2002 The preservation and identification of piñon resins by GC-MS in pottery from the western Great Basin. Archaeometry. 44: 95–105.

Eerkens, J.W. 2002 The preservation and identification of Piñon resins by GC-MS in pottery from the western Great Basin. Archaeometry 44: 95–105.

Evershed, R.P. 2008 Organic residue analysis in archaeology: the archaeological biomarker revolution. Archaeometry 50: 895–924.

Evert, R.F. 2006 Esau's Plant Anatomy. New Jersey: Wiley & Sons, Inc.

Falholt, K., B. Lund and W. Falholt 1973 An easy colorimetric micromethod for routine determination of free fatty acids in plasma. Clinica Chimica Acta 46: 105–211.

Faridah, Q.Z., A.H.A. Abdelmageed, H.A. Nor and Y. Muhamad 2010 Comparative study of essential oil composition of leaves and rhizomes of Alpinia conchigera Griff. at different post-harvest drying periods. Journal of Medicinal Plants Research 4(24): 2700–2705.

Fiehn, O., Kopka, J., Trethew ey, R. N., and Willmitzer, L. 2000 Identification of uncommon plant metabolites based on calculation of elemental compositions using gas chromatography and quadrupole mass spectrometry. Analytical Chemistry 72(15): 3573–3580.

Field, J.H., L. Kealhofer, R. Cosgrove, A.C.F. Coster. 2016 Human-environment dynamics during the Holocene in the Australian Wet Tropics of NE Queensland: a starch and phytolith study. Journal of Anthropology and Archaeology 44: 216–234.

Fievez, V., E. Colman, J.M. Castro-Montoya, I. Stefanov and B. Vlaeminck 2012 Milk odd-and branched-chain fatty acids as biomarkers of rumen function—An update. Animal Feed Science and Technology 172(1): 51–65.

Florin, S. A., Roberts, P., Marwick, B., Patton, N. R., Shulmeister, J., Lovelock, C. E., Barry, L.A., Hua, Q., Nango, M., Djandjomerr, D., Fullagar, R., Wallis, L.A. and C. Clarkson 2021. Pandanus nutshell generates a palaeoprecipitation record for human occupation at Madjedbebe, northern Australia. *Nature ecology & evolution*, *5*(3): 295-303.

Fountain, D.W., C.A. Cornford and G.J. Shaw 1995 Benzoic acid and hydroxylated benzoic acids in pollen. Grana 34(3): 213–216.

Fox, G. and M. Garde 2018 An-Me Arri-ngun: Traditional Plant Foods of the Kundjeyhmi People of Kakadu National Park: The Food We Eat. Gundjeihmi Aboriginal Corporation, Jabiru.

Fullagar, R., B. Stephenson and E.H. Hayes 2017 Grinding grounds: function and distribution of grinding stones from an open site in the Pilbara, Western Australia. Quaternary International 427: 175–183.

Fullagar, R., E. Hayes, B. Stephenson, J. Field, C. Matheson, N. Stern and K. Fitzsimmons 2015 Evidence for Pleistocene seed grinding at Lake Mungo, south-eastern Australia. Archaeology in Oceania 50: 3–19.

Fullagar, R., Liu, L., Bestel, S., Jones, D., Ge, W., Wilson, A., Zhai, S., 2012. Stone tool-use experiments to determine the function of grinding stones and denticulate sickles. Bulletin of the Indo-Pacific Prehistory Association 32: 29–34.

Gaikwad, M., S. Kaleand S. Bhandare 2011 Extraction, characterization and comparison of fixed oil of Moringa oleifera L and Moringa concanensis Nimmo Fam Moringaceae. International Journal of PharmTech Research 3: 1567–1575.

Garelnabi, M., D. Litvinov and S. Parthasarathy 2010 Evaluation of a gas chromatography method for azelaic acid determination in selected biological samples. North American Journal of Medical Sciences 2(9): 397–402.

Gnanamuthu, G., and K. Rameshkumar 2014 Biochemical and fatty acid analysis of faeces in umblachery cattle (Bos indicus) during different phases of estrous cycle. Research Journal of Animal, Veterinary and Fishery Sciences 2(1): 1–5.

Gopalakrishnan, S. and E. Vadivel 2011 GC-MS analysis of some bioactive constituents of Mussaenda frondosa Linn. International Journal of Pharma and Bio Sciences 2(1): 113–120

Goren-Inbar, N., G. Sharon, Y. Melamed and M. Kislev 2002 Nuts, nut cracking, and pitted stones at Gesher Benot Ya'aqov, Israel. Proceedings of the National Academy of Science 99(4): 2455–60.

Gutiérrez, A., J.C. del Río, F.J. González-Vila and F. Martin 1999 Chemical composition of lipophilic extractives from Eucalyptus globulus labill. wood. Holzforschung 53: 481–486.

Hadi, M., B. Kashefi, A. Sobhanipur and M. Rezaarabsorkhi 2013 Study on effect of some medicinal plant extracts on growth and spore germination of Fusarium oxysporum schlecht. In vitro. American-Eurasian Journal of Agricultural and Environmental Sciences 13 (4): 581–588.

Hammami, S., A. Nguir, D. Saidana, J. Cheriaa and Z. Mighri 2011 Chemical analysis and antimicrobial effects of essential oil from Limoniastrum guyonianum growing in Tunisia. Journal of Medicinal Plants Research 5: 2540–2545.

Hamon, C. 2008 Functional analysis of stone grinding and polishing tools from the earliest Neolithic of north-western Europe. Journal of Archaeological Science 35: 1502–1520.

Hassan, W.H., A.A. El Gamal, E. El-Sheddy, M. Al-Oquil and N. Nayyar Farshori 2014 The chemical composition and antimicrobial activity of the essential oil of Lavandula coronopifolia growing in Saudi Arabia. Journal of Chemical and Pharmaceutical Research 6(2): 604–615.

Hayes, E.H. 2015 What was ground? A functional analysis of grinding stones from Madjedbebe and Lake Mungo, Australia. Unpublished PhD Thesis, University of Wollongong, New South Wales, Australia.

Hayes, E.H., C. Pardoe and R. Fullagar 2018a Sandstone grinding/pounding tools: Use-trace reference libraries and Australian archaeological applications. Journal of Archaeological Science: Reports 20: 97–114.

Hayes, E.H., C. Spry, R. Fullagar, A. Tuechler, P. Schell and M. Goulding 2020 Tool-use experiments to determine the function of an incised ground stone artefact with potential symbolic significance. Journal of Lithic Studies 7(30): 1–17.

Hayes, E.H., J.H. Field, A.C.F. Coster, R. Fullagar, C. Matheson, S.A. Florin, M. Nango, D. Djandjomerr, B. Marwick, L.A. Wallis and M.A. Smith 2021 Holocene grinding stones at Madjedbebe reveal the processing of starchy plant taxa and animal tissue. Journal of Archaeological Science: Report, 35: p.102754.

Hayes, E.H., R. Fullagar, K. Mulvaney and K. Connell 2018b Food or fibercraft? Grinding stones and Triodia grass (spinifex). Quaternary International 468: 271–283.

Haytowitz, D.B., and R.H. Matthews 1984 Composition of foods: vegetables and vegetable products: raw, processed, prepared. USDA Agriculture Handbook no. 8-11; U.S. Department of Agriculture: Washington.

Heigenmoser, A., F. Liebner, E. Windeisen and K. Richter 2013 Investigation of thermally treated beech (Fagus sylvatica) and spruce (Picea abies) by means of multifunctional analytical pyrolysis-GC/MS. Journal of Analytical and Applied Pyrolysis 100: 117–126.

Helen, P.M. and A.M. Anil 2011 Analysis of essential oil constituents and in vitro antimicrobial screening of Kaempferia galangal. Advanced Biotechnology 10(10): 07–09.

Holloway, P.W., and S.J. Wakil 1964 Synthesis of fatty acids in animal tissues II. The occurrence and biosynthesis of cis-vaccenic acid. Journal of Biological Chemistry 239(8): 2489–2495.

Hossain, M.A., M.D. Shah and M. Sakari 2011 Gas chromatography–mass spectrometry analysis of various organic extracts of Merremia borneensis from Sabah. Asian Pacific Journal of Tropical Medicine 4(8): 637–641.

Igwe, O.U., and D.E. Okwu 2013 GC-MS evaluation of bioactive compounds and antibacterial activity of the oil fraction from the seeds of Brachystegia eurycoma (HARMS). Asian Journal of Plant Science and Research 3(2): 47–54.

Janakiraman, N., J. Jasmin Jansi, M. Johnson, S. Jeeva and T. Renisheya Joy Jeba Malar 2012 Phytochemical analysis on Asystasia gangetica (L.) T. Anderson. Journal of Harmonized Research in Pharmacy 1(1): 19–32.

Jensen, W.A. 1962 Botanical Histochemistry – Principles and Practice. Berkeley: W.H. Freeman and Company.

Jeong, J.M. and S.M. Kim 2014 Bioactive materials: simultaneous determination of benzoic acid, caffeic acid and clorogenic acid in seeds of Eriobotrya japonica and their antibacterial effect. Journal of Applied Biological Chemistry 57(1): 89–93.

Jerković, I. and Z. Marijanović 2010 Oak (Quercus frainetto Ten.) honeydew honey—approach to screening of volatile organic composition and antioxidant capacity (DPPH and FRAP assay). Molecules 15(5): 3744–3756.

Jones, C.J., D. Hare and S.J. Compton 1989 Measuring plant protein with the Bradford Assay: evaluation and standard method. Journal of Chemical Ecology 15: 979–992.

Jones, R. and B. Meehan 1989 Plant foods of the Gidjingali: ethnographic and archaeological perspectives from northern Australia on tuber and seed exploitation. In D.R. Harris and G.C. Hillman (Eds), Foraging and Farming: The Evolution of Plant Exploitation, pp.120–135. London: Unwin Hyman Ltd.

Judefeind, A., P.J. van Rensburg, S. Langelaar, J.W. Wiechers and J. du Plessis 2008 Quantitative determination of octadecenedioic acid in human skin and transdermal perfusates by gas chromatography-mass spectrometry. Journal of Chromatographic Science 46(6): 544–550.

Kaal, J., A. Martínez-Cortizas and K.G Nierop 2009 Characterisation of aged charcoal using a coil probe pyrolysis-GC/MS method optimised for black carbon. Journal of Analytical and Applied Pyrolysis 85(1): 408–416.

Kaal, J., A. Martínez-Cortizas, K.G. Nierop and P. Buurman 2008 A detailed pyrolysis-GC/MS analysis of a black carbon-rich acidic colluvial soil (Atlantic ranker) from NW Spain. Applied Geochemistry 23(8): 2395–405.

Kalt F.R., and I.E. Cock 2014 Gas chromatography-mass spectroscopy analysis of bioactive petalostigma extracts: Toxicity, antibacterial and antiviral activities. Pharmacognosy Magazine 10(37): 37.

Kanzaki, G. and E.Y. Berger 1959 Colorimetric determination of methylcellulose with Diphenylamine. Analytical Chemistry 31: 1383–1385.

Kaushik, P., S. Lai, A.C. Rana and D. Kaushik 2014 GC-MS Analysis of Bioactive Constituents of Pinus roxburghii Sarg.(Pinaceae) from Northern India. Research Journal of Phytochemistry 8(2): 42–46.

Kehrwald, N., R. Zangrando, P. Gabrielli, J.L. Jaffrezo, C. Boutron, C. Barbante and A. Gambaro 2012 Levoglucosan as a specific marker of fire events in Greenland snow. Tellus Series B: Chemical and Physical Meteorology 64: 1–9.

Knudsen, J.L. and L. Tollsten 1993 Trends in floral scent chemistry in pollination syndromes: floral scent composition in moth-pollinated taxa. Botanical Journal of the Linnean Society 113: 263–284.

Kraybill, N. 1977 Pre-agricultural tools for the preparation of foods in the Old World. In C.A. Reeds, (ed), Origins of Agriculture, pp.485–521. Ninth International Congress of Anthropological and Ethnological Science, Chicago, 1976. The Hague: Mouton Publishers.

Krishna, N.V., V. Raman, K. R. Babu and C. Apparo 2012 Antioxidant activity and GC-MS analysis of phragmytes vallatoria leaf ethanolic extract. International Research Journal of Pharmacy 3(3): 252–254.

Kruger, N.J. 1994 The Bradford methods for protein quantification. In J.M. Walker (ed), Methods in Molecular Biology, Volume 32, Basic Protein and Peptide Protocols, pp.9–15. Totowa, NJ: Humana Press.

Kumar, D., S. Arora and A. Verma 2013 Fatty acid composition and antimicrobial and antioxidant activity of Cassia glauca seed extracts. International Journal of Phytopharmacology 4(2): 113–118.

Kumar, R.Z.A. and A. Bhaskar 2014 Determination of bioactive components from the ethanolic peel extract of Citrus reticulata by Gas chromatography–Mass Spectrometry. International Journal of Drug Development and Research 4(4): 166–174.

Kumar, S.T., A. Anandan and M. Jegadeesan 2012 Identification of chemical compounds in Cissus quadrangularis L. variant I of different samples using GC-MS analysis. Archives of Applied Science Research 4(4): 1782–1787.

Kuo, P.C., M.L. Yang, P.L. Wu, H.N. Shih, T.D. Thang, N.X. Dung and T.S. Wu 2008 Chemical constituents from Abutilon indicum. Journal of Asian Natural Products Research 10(7): 689–693.

Lacheva, M. 2014 GC-MS studies of the chemical composition of two inedible Agaricus species (Section Xanthodermatei) in Bulgaria. Journal of Biodiversity and Environmental Sciences 4(4): 287–295.

Lachman, J., A. Hejtmankova, J. Sýkora, J. Karban, M. Orsák and B. Rygerová 2010 Contents of major phenolic and flavonoid antioxidants in selected Czech honey. Czech Journal of Food Sciences 28(5): 412–426.

Lakshmi prava, M., A. Ahmad, T.R. Krishna and B.S. Bellubbi 2012 A study on composition and structure of beeswax. Journal of Chemical, Biological and Physical Sciences 2(4): 2036–2043.

Lakshmi, T.V., and P. Rajalakshmi 2011 Identification of phyto-components and its biological activities of aloe vera through the gas chromatography-mass spectrometry. International Research Journal of Pharmacy 2(5): 247–249.

Lamb, J. and T. Loy 2005 Seeing Red: the use of Congo Red dye to identify cooked and damaged starch grains in archaeological residues. Journal of Archaeological Science 32: 1433–1440.

Latif, M.T., C.S. Mei, N.M. Hanif and T. Srithawira 2012 Levoglucosan as an indicator of biomass burning from selected tropical plants. Environment Asia 5(2): 22–27.

Liisberg, M.F. 1968 Rhodamine B as an extremely specific stain for cornification. Acta Anatomica 69(1): 52–57.

Lillie, R.D. 1976 H.J. Conn's Biological Stains. Baltimore: Williams and Wilkins.

Liu, L., J. Field, R. Fullagar, S. Bestel, X. Chen and X. Ma 2010b What did grinding stones grind? New light on Early Neolithic subsistence economy in the Middle Yellow River Valley, China. Antiquity 84: 816–833.

Liu, L., P. Howe, Y. Zhou, Z. Xu, C. Hocart, R. Zhanga 2000 Fatty acids and b-carotene in Australian purslane (Portulaca oleracea) varieties. Journal of Chromatography: A 893(2000): 207–213.

Liu, L., S. Ma and J. Cui 2014 Identification of starch granules using a two-step identification method. Journal of Archaeological Science 52: 421–427.

Louderback, L.A., N.M., Herzog and B.M. Pavlik, 2017 A new approach for identifying starch granules of wild food plants from arid western North America. Starch-Stärke, 69(5–6): p.1600167.

Luong, S., E.H. Hayes, E. Flannery, T. Sutikna, M.W. Tocheri, E.W. Saptomo and R.G. Roberts 2017 Development and application of a comprehensive analytical workflow for the quantification of non-volatile low molecular weight lipids on archaeological stone tools. Analytical Methods 9(30): pp.4349–4362.

Luong, S., M.W. Tocheri, E.H. Hayes, T. Sutikna, R. Fullagar, E.W. Saptomo and R.G. Roberts 2019 Combined organic biomarker and use-wear analyses of stone artefacts from Liang Bua, Flores, Indonesia. Scientific Reports 9(1): 1-17.

Luong, S., M.W. Tocheri, T. Sutikna, E.W. Saptomo and R.G. Roberts, 2018 Incorporating terpenes, monoterpenoids and alkanes into multiresidue organic biomarker analysis of archaeological stone artefacts from Liang Bua (Flores, Indonesia). Journal of Archaeological Science: Reports 19: 189–199.

Mahalingam, R., R. Bharathidasan, V. Ambikapathy and A. Panneerselvam 2012 Phytochemical compound analysis of Pandanus odoratissimum. Asian Journal of Plant Science and Research 2(3): 228–231.

Mahdi, J. G. 2010 Medicinal potential of willow: a chemical perspective of aspirin discovery. Journal of Saudi Chemical Society 14(3): 317–322.

Maia, M. and F.M. Nunes 2013 Authentication of beeswax (Apis mellifera) by high-temperature gas chromatography and chemometric analysis. Food Chemistry 136(2): 961–968.

Malarvizhi, P., and N. Ramakrishian 2011 GC-MS analysis of biologically active compounds in leaves of Calophyllum inophyllum L. International Journal of Chemtech Research 3(2): 806–809.

Marreiros, J., I. Calandra, W. Gneisinger, E. Paixão, A. Pedergnana and L. Schunk 2020 Rethinking use-wear analysis and experimentation as applied to the study of past hominin tool use. Journal of Paleolithic Archaeology 3(3): 475-502.

Maruthupandian, A. and V.R. Mohan 2011 GC-MS analysis of ethanol extract of Wattakaka volubilis (lf) stapf. leaf. International Journal of Phytomedicine 3(1): 59–62.

Marwick, B., Hayes, E., Clarkson, C. and R. Fullagar 2017 Movement of lithics by trampling: An experiment in the Madjedbebe sediments, northern Australia. *Journal of Archaeological Science 79*: 73–85.

Masuko, T., A. Minami, N. Iwasaki, T. Majima, S-I. Nishimura and Y. Lee 2005. Analytical Biochemistry 339: 69–72.

Matheson C.D. and M. Veall 2014. Presumptive blood test using Hemastix® with EDTA in Archaeology. Journal of Archaeological Science 41: 230–241.

Maya, K.M., T.J. Zachariah, K.S. Krishnamurthy, J. Rema and B. Krishnamoorthy 2006 Fatty acids and leaf amino acids in Myristica fragrans Houtt and related taxa. Indian Journal of Horticulture 63(3): 316–318.

McCarthy, F. D. 1976. Australian Aboriginal Stone Implements. Sydney: Australian Museum Trust.

McCready, R.M. and W.Z. Hassid 1943 Separation and quantitative estimation of amylose and amylopectin in potato starch. Journal of American Chemical Society 65: 1154–1157.

Meehan, B., P. Gaffey and R. Jones 1978 Fire to steel: Aboriginal exploitation of pandanus and some wider implications. In P.K. Lauer (Ed), Readings in Material Culture, pp.73–96. Occasional papers in Anthropology. Brisbane: Anthropology Museum, University of Queensland.

Merlin, N.J., V. Parthasarathy, R. Manavalan and S. Kumaravel 2009 Chemical investigation of aerial parts of Gmelina asiatica Linn by GC-MS. Pharmacognosy Research 1(3): 152.

Michalski, S., R. Shaler, F.L. Dorman 2013 The evaluation of fatty acid ratios in latent fingermarks by gas chromatography/mass spectrometry (GC/MS) analysis. Journal of Forensic Science 58: S215–S220.

Minzangi, K., A.N. Kaaya, F. Kansiime, J.R.S. Tabuti, B. Samvura and O. Grahl-Nielsen 2011 Fatty acid composition of seed oils from selected wild plants of Kahuzi-Biega National Park and surroundings, Democratic Republic of Congo. African Journal of Food Science 5(4): 219–226.

Miyatani, S., K. Yamamoto, T. Nakayama, A. Kinoshita and A. Shibahara 2001 Occurrence of cis-vaccenic acid in root vegetables. Japanese Journal of Food chemistry 8(3): 184–188.

Murugesan, S., and A. Panneerselvam 2013 Evaluation of phytochemical constituents from stems of Memecylon umbellatum brum. F. by GC-MS analysis. Research and Reviews: Journal of Botanical Sciences 2(4): 29–24.

Musaubach, M., A. Plos and P. Babot 2013 Differentiation of archaeological maize (Zea mays L.) from native wild grasses based on starch grain morphology: cases from the central Pampas of Argentina. Journal of Archaeological Science 40: 1186–1193.

Nayak, S., A.K. Jena, D.K. Mittal, and D. Joshi 2014 GC-MS analysis of phytocostituents of some wild zingiberaceae plants methanolic rhizome extracts. Research in Plant Sciences 2(1): 1–5.

Neff, H. 2003 Analysis of Mesoamerican plumbate pottery surfaces by laser ablation-inductively coupled plasma-mass spectrometry (LA-ICP-MS). Journal of Archaeological Science 30(1): 21–35.

Nicolet, B.H. and L.M. Liddle 1916 The occurrence of azelaic acid as a product of the spontaneous oxidation of fats. Industrial and Engineering Chemistry 8(5): 416–417.

O'Connell, J. F., Allen, J., Williams, M. A., Williams, A. N., Turney, C. S., Spooner, N. A., Kamminga, J., Brown, G., and A. Cooper 2018 When did *Homo sapiens* first reach Southeast Asia and Sahul? *Proceedings of the National Academy of Sciences*, *115*(34): 8482–8490.

Allen, J. and J.F. O'Connell 2020 A different paradigm for the initial colonisation of Sahul. *Archaeology in Oceania*, *55*(1): 1-14.

Odell, G.H. 1977 The Application of Micro-wear Analysis to the Lithic Component of an Entire Prehistoric Settlement: Methods, Problems and Functional Reconstructions. Unpublished PhD thesis, Department of Anthropology, Harvard University.

Ogunlesi, M., W. Okiei, M. Ademoye and E.A. Osibote 2010 Analysis of essential oil from the stem of Chansmanthera dependens. Journal of Natural Products 3: 47–53.

Ololade, Z.S. and N.O. Olawore 2013 Chemistry and medicinal potentials of the seed essential oil of Eucalyptus toreliana F. Muell grown in Nigeria. Global Journal of Science Frontier Research 13(3): 1–9.

Olutayo, O., O. Abayomi, F. Olakunle, A. Temitope and I. Ibrahim 2013 The chemical composition of essential oil from the root of Cissampelos owariensis (p. beauv) and free radical scavenging activities of its extracts. African Journal of Pure and Applied Chemistry 7(6): 225–230.

Omikorede, O.E., O.A. Lawal and O.A. Iresemowo 2012 Volatile constituents, antibacterial and insecticidal activities of essential oil from the leaves of Vitex agnus-castus L.(Verbenaceae). Canadian Journal on Computing in Mathematics, Natural Sciences, Engineering and Medicine 3(7): 256–260.

Peterson, N. 1968 The pestle and mortar: an ethnographic analogy for archaeology in Arnhem Land. Mankind 6: 567–570.

Piperno, D.R., A.J. Ranere, I. Holst and P. Hansell 2000 Starch grains reveal early crop horticulture in the Panamanian tropical forest. Nature 407(6808): 894–897.

Prakash, O., M. Gondwal and A.K. Pant 2011 Essential oils composition and antioxidant activity of water extract from seeds and fruit pulp of Skimmia anquetilia NP Taylor and Airy Shaw. Indian Journal of Natural Products and Resources 2(4): 435–441.

Priyanka, C., P. Kumar, S. Bankar and L. Karthik 2014 In vitro antibacterial activity and GCMS analysis of Acacia karoo and Ziziphus mauritiana extracts. Journal of Taibah University for Science 9(1): 13–19.

Purdie, S., P. Patrick, L. Hyadbi, P. Thomas, D. Fletcher, G. Barrett, M. Ramsey, D. Watbi, M. M. Martin, M. Thomas, M. Thomas, M, P. Widalji, F. Kofod, S. Thomas, P. Mung Mung, R. Peters, J. Blythe and G. Wightman 2018 Gija Plants and Animals: Aboriginal Flora and Fauna Knowledge from the East Kimberley, North Australia. Northern Territory Botanical Bulletin No. 47.

Ramesh, H.P. and R.N. Tharanathan 1999 Water-extracted polysaccharides of selected cereals and influence of temperature on the extractability of polysaccharides in sorghum. Food Chemistry 64(3): 345–350.

Ranganathan, D. 2014 Phytochemical analysis of Caralluma nilagiriana using GC–MS. Journal of Pharmacognosy and Phytochemistry 3(1): 155–159.

Rao, G.V., C. Sharlene and T. Mukhopadhyay 2012 Secondary metabolites from the flowers of Mimusops elengi Linn. Der Pharmacia Lettre 4(6): 1817–1820

Reddy, S., K.R.-M. Ammani, N. Rajesh, D. Aravind and C. Bala Sekaran 2014 Phytochemical and GC-MS analysis of Commiphora caudata (Wt. and Arn.) Eng. Bark. Indian Journal of Advances in Plant Research 1(5): 24–29.

Refaat, J., M.S. Kamel, M.A. Ramadan and A.A. Ali 2012 Crinum; an endless source of bioactive principles: a review. Part 1-Crinum alkaloids: lycorine-type alkaloids. International Journal of Pharmaceutical Sciences and Research 3: 1883–1890.

Regert, M., S. Colinart, L. Degrand and O. Decavallas 2001 Chemical alteration and use of beeswax through time: accelerated ageing tests and analysis of archaeological samples from various environmental contexts. Archaeometry 43(4): 549–569.

Remya, M.R. and O.P. Saj 2013 GC/MS studies on the leaf essential oil of Mikania scandens (L) wild. Asian Journal of Biomedical and Pharmaceutical Sciences 3(19): 41–43.

Ren, Q., and Y. Tian 2012 Studies of aroma active components in naked oat by GC-MS. Journal of Food, Agriculture and Environment 10(3-4): 67–71.

Ren, Y., D.W. Zhang and S.J. Dai 2009 Chemical constituents from Solanum lyratum. The Chinese Journal of Natural Medicines 7(3): 203–205.

Renjie, L., L. Zhenhong and S. Shidi 2010 GC-MS analysis of fennel essential oil and its effect on microbiology growth in rats' intestine. African Journal of Microbiology Research 4: 1319–1323.

Rosy, B.A. and P.J. Rosakutty 2012 GC-MS analysis of methanol wild plant and callus extracts from three Cissus species, Family Vitaceae. Journal of Chemical and Pharmaceutical Research 4(7): 3420–3426.

Russell Smith, J., D. Lucas, M. Gapindi, B. Gunbunuka, N. Kapirigi, G. Namingum, K. Lucas, P. Giuliani, and G. Chaloupka 1997 Aboriginal resource utilization and fire management practice in western Arnhem Land, monsoonal Northern Australia: notes for prehistory, lessons for the future. Human Ecology 25(2): 159–195.

Russell-Smith, J. 1985 Studies in the jungle: people, fire and monsoon forest. In R. Jones (Ed), Archaeological Research in Kakadu National Park, pp.241–258. Canberra: Australian National Parks and Wildlife Service.

Sáez, A., S. Montoya, J. Cabrera, C. Asensio and E. Ortega 2014 Characterisation and lipid profile of macadamia nuts (Macadamia integrifolia and Macadamia tetraphylla). International Journal of Engineering and Applied Sciences 4(9): 33–39.

Sağlık, S., K. Alpınar and S. İmre 2002 Fatty acid composition of Dracunculus vulgaris Schott (Araceae) seed oil from Turkey. Journal of Pharmacy and Pharmaceutical Sciences 5: 231–233.

Salem, A.Z.M., M.Z.M. Salem, M. González-Ronquillo, L.M. Camacho and M. Cipriano 2011 Major chemical constituents of Leucaena leucocephala and Salix babylonica leaf extracts. Journal of Tropical Agriculture 49(1-2): 95–98.

Santana, O., M. Reina, A. L. Anayac, F. Hernández, M. E. Izquierdoa and A. González-Colomaa 2008 3-O-Acetyl-narcissidine, a bioactive alkaloid from Hippeastrum puniceum Lam. (Amaryllidaceae). Verlag der Zeitschrift für Naturforschung, Tübingen 63(9-10): 639–643.

Santhosh Kumar, S., P. Samydurai, R. Ramakrishnan and N. Nagarajan 2014 Gas chromatography and mass spectrometry analysis of bioactive constituents of Adiantum cappillus-veneris L. International Journal of Pharmacy and Pharmaceutical Sciences 6(4): 60–63.

Sarada, K., R.J. Margret and V.R. Mohan 2011 GC–MS Determination of Bioactive Components of Naringi crenulata (Roxb) Nicolson. International Journal of ChemTech Research 3(3): 1548–1555.

Saravanan, P., G. Chanramohan, J. Mariajancyrani and P. Shanmugasundaram 2014 GC-MS analysis of phytochemical constituents in ethanolic bark extract of Ficus religiosa linn. International Journal of Pharmacy and Pharmaceutical Sciences 6(1): 457–460.

Sathya, S., S. Srisudha and P. Gunasekaran 2012 Growth rate, pigment composition and fatty acid profile of Chlorella pyrenoidosa. International Journal of Biological and Pharmaceutical Research 3(5): 677–683.

Senanayake, M.J. 2006 A Chemical Investigation of New Zealand Unifloral Honeys. Unpublished PhD thesis, University of Waikato, Hamilton.

Senthilkumar, N., S. Murugesan and K.B. Vijayalakshmi 2012 GC-MS-MS analysis of Trichilia connaroides (Wight and Arn.) Bentv (Meliaceae): A tree of ethnobotanical records. Asian Journal of Plant Science and Research 2(2): 193–197.

Sharopov, F.S., I.S. Gulmurodov and W.N. Setzer 2010 Essential oil composition of Hypericum perforatum L. and Hypericum scabrum L. growing wild in Tajikistan. Journal of Chemical and Pharmaceutical Research 2(6): 284–290.

Sheela, D. and F. Uthayakumari 2013 GC-MS analysis of bioactive constituents from coastal sand dune taxon-sesuvium portulacastrum (L.) Bioscience Discovery 4(1): 47–53.

Shibahara, A., K. Yamamoto, T. Nakayama and G. Kajimoto 1986 cis-Vaccenic acid in mango pulp lipids. Lipids 21(6): 388–394.

Siddiquee, S., B.E. Cheong, K. Taslima, H. Kausar and M.M. Hasan 2012 Separation and identification of volatile compounds from liquid cultures of Trichoderma harzianum by GC-MS using three different capillary columns. Journal of Chromatographic Science 50(4): 358–367.

Sivagurunathan, A.X. 2014 GC-MS evaluation of bioactive compounds of Marsilea quadrifolia Linn (aquatic fern). International Journal for Pharmaceutical Research Scholars 3(1): 164–170.

Smith, N.M. 1991 Ethnobotanical field notes from the Northern Territory, Australia. Journal of Adelaide Botanical Gardens 14(1): 1–65.

Smith, M. A., Ward, I. and I. Moffat 2020 How do we distinguish termite stone lines from artefact horizons? A challenge for geoarchaeology in tropical Australia. *Geoarchaeology*, *35*(2): 232–242.

Smith, M., Ward, I. and I. Moffat 2021 Letter to the editors on termite stone lines. *Geoarchaeology*, *36*(2): 363–365.

Sodipo, O.A., F.I. Abdulrahman, T.E. Alemika and I.A. Gulani 2012 Chemical composition and biological properties of the petroleum ether extract of Solanum macrocarpum L.(Local Name: Gorongo). British Journal of Pharmaceutical Research 2(2): 108–128.

Soleimani, M., P.A Azar, M. Saber-Tehranil and A. Rustaiyan 2009 Volatile composition of Ruta graveolens L. of North of Iran. World Applied Sciences Journal 7(1): 124–126.

Spry, C., E.H., Hayes, R. Fullagar, A. Tuechler, A., P. Schell and M. Goulding 2019 An unusual, incised ground stone artefact from southwestern Victoria, Australia: its function and potential symbolic significance. Australian Archaeology 85(1): 95–101.

Srebotnik, E. and K. Messner 1994 A simple method that uses differential staining and light microscopy to assess the selectivity of wood delignification by white rot fungi. Applied and Environmental Microbiology 60(4): 1383–1386.

Stadelmann, E.J., and H. Kinzel 1972 Vital staining of plant cells. Methods in Cell Physiology 5: 325.

Stringer, R., I. Labunska, D. Santillo, P. Johnston, J. Siddorn and A. Stephenson 2000 Concentrations of phthalate esters and identification of other additives in PVC children's toys. Environmental Science and Pollution Research 7(1): 27–36.

Sujatha, M., K. Karthika, S. Sivakamasundari, J. Mariajancyrani and G. Chandramohan 2014 GC-MS analysis of phytocomponents and total antioxidant activity of hexane extract of Sinapis alba. International Journal of Pharmaceutical, Chemical and Biological Science 4(1): 112–117.

Sutha, S., V.K. Devi and V.R. Mohan 2011 GC-MS determination of bioactive components of Erythropalum scandens Bl., Bijdr. Journal of Applied Pharmaceutical Science 1(9): 170–173.

Takenaka, Y., N. Morimoto, N. Hamada and T. Tanahashi 2011 Phenolic compounds from the cultured mycobionts of Graphis proserpens. Phytochemistry 72(11): 1431–1435.

Thomson, D.F. 1939 The seasonal factor in human culture: illustrated from the life of a contemporary nomadic group. Proceedings of the Prehistoric Society 5: 209–221.

Tyagi, R. and V. Sharma 2014 A comparison of volatile compounds in different genotypes of Sesamum indium L. by GC-MS. International Journal of Pharmaceutical Sciences and Research 5(1): 249–258.

Ugoeze, K.C., T. Ehianeta, C. Alaribe and C. Anyakora 2014 Analysis and identification of oils from seed extract of Anthonotha macrophylla using gas chromatography-mass spectrometry (GC-MS). African Journal of Biotechnology 13(22): 2260–2264.

Van Bergen, P.F. and I Poole 2002 Stable carbon isotopes of wood: a clue to palaeoclimate? Palaeogeography, Palaeoclimatology, Palaeoecology 182(1): 31–45.

Van Peer, P., R. Fullagar, S. Stokes, R.M. Bailey, J. Moeyersons, F. Steenhoudt, A. Geerts, T. Vanderbeken, M. De Dapper and F. Geus 2003 The early to Middle Stone Age transition and the emergence of modern human behaviour at site 8-B-11, Sai Island, Sudan. Journal of Human Evolution 45: 187–193.

Villa, P., L. Pollarolo, I. Degano, L. Birolo, M. Pasero, C. Biagioni, K. Douka, R. Vinciguerra, J.J. Lucejko, and L. Wadley 2015 A milk and ochre paint mixture used 49,000 years ago at Sibudu, South Africa. PLoS ONE, 10: e0131273.

Wang, H.J., X.L. Wu, T.Z. Zhou, X.M. Deng and D.C. Wang 2012 Chemical constituents from the fruits of Kadsura marmorata. Journal of Chinese Medicinal Materials 35(3): 396–399.

Wang, Y.H., B. Avula, N.D. Nanayakkara, J. Zhao and I.A. Khan 2013 Cassia cinnamon as a source of coumarin in cinnamon-flavored food and food supplements in the United States. Journal of Agricultural and Food Chemistry 61(18): 4470–4476.

Weise, T., M. Kai, A. Gummesson, A. Troeger, S. von Reuß, S. Piepenborn and B. Piechulla 2012 Volatile organic compounds produced by the phytopathogenic bacterium Xanthomonas campestris pv. Vesicatoria. Journal of Organic Chemistry 8(1): 579–596.

Wesołowska, A., D. Jadczak and M. Grzeszczuk 2011 Chemical composition of the pepper fruit extracts of hot cultivars Capsicum annuum L. Acta Scientiarum Polonorum: Hortorum Cultus 10(1): 171–184.

Wessely, Z., S.H. Shapiro, J.L. Klavins and H.M. Tinberg 198. Identification of mallory bodies with Rhodamine B fluorescence and other stains for keratin. Stain Technology 56(3): 169–176.

Wightman, G. and N.M. Smith 1991 Ethnobotany, Vegetation and Floristics of Milingimbi, Northern Australia. Northern Territory Botanical Bulletin No. 6, Conservation Commission of the Northern Territory.

Williams, M. A., Spooner, N. A., McDonnell, K., and J. F. O'Connell 2021 Identifying disturbance in archaeological sites in tropical northern Australia: Implications for previously proposed 65,000-year continental occupation date. *Geoarchaeology*, *36*(1): 92–108.

Wilson, T.M. 1907 On the chemistry and staining properties of certain derivatives of the methylene blue group when combined with eosin. The Journal of Experimental Medicine 9(6): 645–670.

Wright, K.I. 1994 Ground-stone tools and hunter-gatherer subsistence in Southwest Asia: implications for the transition to farming. American Antiquity 59(2): 238–263.

Xue, H.Q., R.G. Upchurch and P. Kwanyuen 2008 Relationships between oleic and linoleic acid content and seed colonization by Cercospora kikuchii and Diaporthe phaseolorum. Plant Disease 92(7): 1038–1042.

Yayli, N., F.A. Ayaz, M. Kucukislamoglu and A. Aytekin 2001 Volatile components of Arbutus unedo L. fruits by GC MS. Indian Journal of Chemistry 40B: 173–176.

Yeung, E.C. 1998 A beginners guide to the study of plant structure. In S.J. Karcher (Ed), Proceedings of the 19th Workshop/Conference of the Association for Biology Laboratory Education, pp.125–141. Tested Studies for Laboratory Teaching 19. Lafayette: Purdue University Press.

Zhang, J., X. Wang, O. Yu, J. Tang, X. Gu, X. Wan and C. Fang 2010 Metabolic profiling of strawberry (Fragaria× ananassa Duch.) during fruit development and maturation. Journal of Experimental Botany 62(3): 1103–1119.

Zhao, M.Y., and X.W. Yang 2008 Two new acylgluconic acids from the nearly ripe fruits of Evodia rutaecarpa. Journal of Asian Natural Products Research 10(8): 759–763.

Zulfiqar, A. 1998 Phytochemical studies on the chemical constituents of Silene conoidea and Stocksia Brahuica. Unpublished PhD thesis, Research Institute of Chemistry, University of Karachi, Pakistan.
